# Supplementary material for: Sero-prevalence of 19 infectious pathogens and associated factors among middle-aged and elderly Chinese adults: a cross-sectional study
Source: BMJ Open. 2022 May 9;12(5):e058353. doi: 10.1136/bmjopen-2021-058353 (PMC9086621; doi:10.1136/bmjopen-2021-058353)
Supplement: Supplementary data [file bmjopen-2021-058353supp001.pdf]

Sero-prevalence of 19 infectious pathogens and associated factors among middle-aged and elderly Chinese adults: a cross-sectional study

Supplementary Material

eAppendix

Study population

The study design was a prospective study. Participants who were aged 30 -79 years at enrolment, were selected for the China Kadoorie Biobank study of the causes of major chronic diseases that account for most deaths in the Chinese population. Overall, 515 681 people attended the baseline survey between June 2004 and July 2008, of whom 261 (0.05%) withdrew before completion, 2208 (0.4%) were found subsequently to have inadvertently attended the survey twice at different time points and 1 had major data errors. The estimated population response rate was ~30% (26–38% in the five rural areas and 16–50% in the five urban areas). All individuals who completed a questionnaire and provided consent for participants in the study were included in the present analyses. No participants with a prior history of cardiovascular disease or cancer were excluded.

Follow-up of CKB participants was through linkage to established mortality and morbidity registries, and to the nationwide health insurance system which records all hospitalised episodes. Ethics approval was obtained from the Oxford University, the China National CDC, and also from institutional research boards at the local CDCs in the 10 areas. All participants provided written informed consent.

While the China Kadoorie Biobank is not representative of the Chinese population, it collected data from a large number of participants from 5 rural and 5 urban areas that reflected a wide range of exposures and disease outcomes. The study participants did not receive any payment for their participation in the study.

Repeat measurements

For 100 (5%) of these participants, plasma samples collected at the two subsequent resurveys were also assayed to assess the consistency of sero-status (sero-conversion and sero-reversion rates) of measurements over time.

The agreement between multiple measurements of same individuals at three occasions (baseline, the 1st and the 2nd resurvey) of quantitative MFI antibody levels was evaluated using Intraclass Correlation Coefficients (ICCs).

Table S1. Characteristics of pathogens and pathogen-specific antigens included in the multiplex serology and suggested cut-offs to determine seropositivity

Pathogen	Transmissions [1]	Antigen	Full name, function	Criteria for seropositivity	Cut-off (MFI)*
**Hepatitis viruses**					
Hepatitis B virus (HBV)	Perinatal, blood, semen	HBVAc<br>HBVAe	core antigen<br>soluble nucleocapsid associated antigen	2 positives out of 2	1000<br>1000
Hepatitis C virus (HCV)	blood, semen	1aCore<br>1aNS3	structual antigen<br>protease and RNA helicase activity	2 positives out of 2	**300**<br>150
**Human papillomaviruses (HPV)**					
HPV-16	sexual contact	L1<br>E6<br>E7	major capsid protein<br>oncoproteins<br>oncoproteins		80<br>**100**<br>50
HPV-18	sexual contact	L1	major capsid protein		170
**Human herpesviruses (HHV)**					
HHV-1 (HSV-1/)	sores, saliva, and surface in or around mouth	gG	membrane glycoprotein		54
HHV-2 (HSV-2)	genital surfaces, skin, sores or fluids	mgG unique	membrane glycoprotein		180
Varicella zoster virus (VZV/HHV-3)	blisters, saliva or mucus	gE/gI	envelope glycoproteins		100
Epstein-Barr virus (EBV/HHV-4)	saliva, blood or semen	ZEBRA<br>EA-D<br>VCAp18<br>EBNA (peptid)	replication activator<br>replication (polymerase accessory subunit)<br>capsid protein<br>replication, latent viral infection	≥2 positives out of 4	74<br>110<br>2526<br>411
Human cytomegalovirus (CMV/HHV-5)	saliva, urine, blood, tears, semen or breast milk	pp150 Nter<br>pp52<br>pp28	tegument protein<br>DNA binding protein<br>capsid protein	≥2 positives out of 3	655<br>1101<br>200
HHV-6	saliva	IE1A<br>IE1B	potential transactivator<br>potential transactivator	≥1 positive out of 2	300<br>300
HHV-7	saliva	U14	potential tegument protein		**100**
**Human polyomaviruses (HPyV)**					
HPyV BK (BKV)	respiratory fluids or urine	VP1	major capsid protein		250
HPyV JC (JCV)	respiratory fluids or urine	VP1	major capsid protein		250
HPyV MCV (MCV)	respiratory fluids	VP1	major capsid protein		250
**Other viruses**					
Human immunodeficiency virus (HIV)	blood, semen, vaginal and rectal secretions, or breast milk	gag<br>env	structual antigen<br>structual antigen	2 positives out of 2	1000<br>150
Human T-lymphotropic virus (HTLV)	breast milk, blood,	gag<br>nv	structual antigen<br>structual antigen	2 positives out of 2	700<br>50
**Bacteria/Parasite**					
Helicobacter pylori (H. pylori)	saliva (oral-oral, gastro-oral and fecal-oral)	HyuA<br>GroEL<br>UreA<br>CagA-N/C<br>Catalase<br>HP1564<br>VacA-N/C<br>NapA<br>HP0305<br>HpaA<br>Cad<br>HcpC	hydantoin utilization protein A<br>chaperonin GroEL<br>urease alpha subunit<br>cytotoxin-associated antigen A, pathogenesis<br><br>hypothetical protein<br>vacuolating cytotoxin, pathogenesis<br>neutrophil-activating protein<br>hypothetical protein<br>neuraminyllactose-binding hemagglutinin homolog<br>cinnamyl alcohol dehydrogenase<br>conserved hypothetical secreted protein	≥4 positives out of 12	**280**<br>500<br>**300**<br>1500<br>**300**<br>150<br>385<br>120<br>**130**<br>200<br>100<br>150
Chlamydia trachomatis (C. trachomatis)	sexual contact	pGP3	virulence factor		**300**
Toxoplasma gondii (T. gondii)	undercooked meat, blood	sag1<br>p22	surface protein<br>surface protein	≥1 positive out of 2	50<br>50

MFI: Median Fluorescence Intensities
[1] The route of transmission of some pathogens is not completely understood, and the major route is listed

Yao P, *et al. BMJ Open* 2022; 12:e058353. doi: 10.1136/bmjopen-2021-058353

Table S2. Adjusted odds ratios of sero-positivity for selected pathogens by baseline characteristics

	HBV	HPV-16	HHV-2	VZV	HHV-6	HHV-7	BKV	JCV	MCV	*H. pylori*	*C. trachomatis*	*T. gondii*
Sex, female vs male	0.86 (0.72, 1.03)	**2.07 (1.36, 3.14)**	1.45 (1.04, 2.03)	0.76 (0.54, 1.06)	**1.92 (1.59, 2.31)**	**2.15 (1.67, 2.76)**	1.08 (0.78, 1.48)	0.82 (0.68, 1.00)	1.04 (0.85, 1.25)	0.93 (0.78, 1.12)	1.18 (0.98, 1.42)	1.31 (1.00, 1.71)
Age (reference group: ≤39 years)												
40-49	1.01 (0.77, 1.34)	0.73 (0.38, 1.41)	1.12 (0.66, 1.90)	0.84 (0.52, 1.37)	0.78 (0.58, 1.05)	0.99 (0.66, 1.48)	1.25 (0.78, 2.01)	1.03 (0.78, 1.36)	1.02 (0.77, 1.35)	1.21 (0.91, 1.59)	**1.43 (1.08, 1.91)**	1.33 (0.84, 2.11)
50-59	1.15 (0.87, 1.51)	1.51 (0.84, 2.73)	1.34 (0.80, 2.24)	1.08 (0.65, 1.78)	0.63 (0.47, 0.85)	0.92 (0.62, 1.37)	1.08 (0.68, 1.72)	**1.53 (1.15, 2.03)**	**1.54 (1.16, 2.04)**	**1.37 (1.04, 1.81)**	**2.18 (1.64, 2.90)**	**1.85 (1.18, 2.90)**
≥60	**1.50 (1.12, 2.00)**	**2.06 (1.14, 3.73)**	1.67 (0.99, 2.81)	1.09 (0.64, 1.85)	0.70 (0.52, 0.96)	0.94 (0.62, 1.42)	1.17 (0.71, 1.91)	**2.30 (1.69, 3.13)**	**2.01 (1.48, 2.72)**	1.29 (0.97, 1.73)	**3.51 (2.59, 4.74)**	**2.54 (1.62, 3.99)**
Region, urban vs rural	1.15 (0.96, 1.37)	1.12 (0.78, 1.62)	1.22 (0.89, 1.67)	0.74 (0.53, 1.02)	1.07 (0.89, 1.29)	1.04 (0.81, 1.34)	0.90 (0.66, 1.23)	1.17 (0.97, 1.41)	1.07 (0.89, 1.29)	**1.80 (1.50, 2.15)**	0.92 (0.77, 1.10)	1.14 (0.88, 1.47)
Education, ≥high school vs <high school	0.93 (0.72, 1.19)	**1.63 (1.01, 2.65)**	1.23 (0.81, 1.88)	0.87 (0.57, 1.32)	0.83 (0.64, 1.07)	1.09 (0.77, 1.54)	0.88 (0.58, 1.35)	0.9 (0.70, 1.17)	0.95 (0.73, 1.23)	1.09 (0.85, 1.41)	**0.70 (0.54, 0.90)**	1.16 (0.82, 1.65)
Household income, ≥20k vs <20k	1.03 (0.83, 1.27)	0.84 (0.56, 1.27)	0.89 (0.62, 1.28)	1.26 (0.87, 1.82)	0.89 (0.72, 1.11)	1.06 (0.78, 1.43)	0.95 (0.66, 1.37)	1.03 (0.82, 1.29)	0.98 (0.78, 1.23)	0.89 (0.71, 1.11)	**0.80 (0.64, 0.99)**	1.21 (0.90, 1.62)
Physical activity (high vs low)	1.27 (1.03, 1.56)	**0.50 (0.32, 0.78)**	0.82 (0.57, 1.19)	**0.65 (0.45, 0.94)**	0.91 (0.73, 1.12)	1.44 (1.08, 1.93)	0.88 (0.62, 1.26)	1.06 (0.86, 1.32)	0.99 (0.80, 1.23)	0.98 (0.79, 1.21)	0.88 (0.72, 1.09)	0.75 (0.56, 1.01)
Ever regular smoker (yes vs no)	1.15 (0.86, 1.55)	0.59 (0.31, 1.13)	0.88 (0.51, 1.51)	1.09 (0.63, 1.89)	1.00 (0.74, 1.34)	1.15 (0.79, 1.68)	0.93 (0.56, 1.55)	1.06 (0.77, 1.46)	**0.71 (0.51, 0.97)**	1.03 (0.76, 1.40)	0.92 (0.68, 1.25)	0.77 (0.50, 1.18)
Ever regular drinker (yes vs no)	1.08 (0.81, 1.42)	0.55 (0.27, 1.14)	0.84 (0.48, 1.47)	1.29 (0.75, 2.22)	1.01 (0.76, 1.33)	1.03 (0.72, 1.48)	1.10 (0.68, 1.78)	0.96 (0.72, 1.29)	1.06 (0.79, 1.43)	0.91 (0.69, 1.21)	1.01 (0.76, 1.34)	0.86 (0.56, 1.31)
Self-rated poor health (yes vs no)	0.90 (0.66, 1.23)	1.31 (0.75, 2.27)	1.11 (0.67, 1.82)	1.05 (0.61, 1.82)	0.88 (0.65, 1.19)	**0.60 (0.41, 0.88)**	0.97 (0.57, 1.63)	0.96 (0.69, 1.31)	0.96 (0.70, 1.32)	0.83 (0.61, 1.13)	0.98 (0.72, 1.34)	1.27 (0.85, 1.89)
Hepatitis B test (positive vs negative)	**11.51 (5.16, 25.7)**	0.57 (0.13, 2.40)	0.73 (0.26, 2.09)	0.90 (0.38, 2.13)	0.78 (0.46, 1.31)	1.97 (0.78, 5.02)	1.10 (0.43, 2.80)	1.11 (0.65, 1.90)	1.44 (0.83, 2.50)	1.14 (0.66, 1.97)	1.34 (0.79, 2.27)	0.77 (0.32, 1.82)
Diabetes (yes vs no)	1.20 (0.81, 1.77)	**2.69 (1.52, 4.77)**	1.31 (0.70, 2.45)	0.86 (0.44, 1.67)	0.86 (0.58, 1.28)	0.75 (0.45, 1.26)	1.25 (0.61, 2.55)	**0.52 (0.35, 0.78)**	1.22 (0.78, 1.91)	1.06 (0.71, 1.59)	**1.55 (1.03, 2.34)**	1.28 (0.78, 2.09)
History of peptic ulcer (yes vs no)	1.09 (0.68, 1.74)	0.19 (0.03, 1.38)	0.54 (0.19, 1.51)	1.23 (0.49, 3.11)	0.82 (0.51, 1.32)	1.87 (0.88, 3.98)	0.91 (0.41, 2.04)	0.89 (0.54, 1.47)	0.78 (0.48, 1.27)	**0.52 (0.32, 0.85)**	1.09 (0.67, 1.76)	0.80 (0.39, 1.64)
History of cirrhosis/chronic hepatitis (yes vs no)	**7.51 (2.55, 22.13)**	2.20 (0.62, 7.73)	0.46 (0.06, 3.49)	0.66 (0.19, 2.24)	0.86 (0.39, 1.92)	1.48 (0.44, 5.03)	2.21 (0.30, 16.47)	1.56 (0.64, 3.80)	0.88 (0.39, 1.97)	0.79 (0.35, 1.75)	0.57 (0.24, 1.37)	0.55 (0.13, 2.35)
Family history of cancer	1.13 (0.87, 1.47)	1.05 (0.62, 1.80)	0.92 (0.56, 1.51)	0.86 (0.56, 1.33)	0.94 (0.72, 1.23)	0.85 (0.59, 1.21)	1.38 (0.84, 2.26)	1.19 (0.91, 1.57)	0.99 (0.76, 1.31)	1.18 (0.91, 1.54)	0.82 (0.63, 1.07)	0.96 (0.66, 1.39)
BMI≥25 kg/m^2 (yes vs no)	1.09 (0.89, 1.32)	**1.51 (1.03, 2.22)**	1.05 (0.75, 1.49)	1.03 (0.73, 1.46)	0.93 (0.76, 1.14)	0.80 (0.61, 1.05)	1.06 (0.75, 1.48)	1.00 (0.82, 1.23)	1.12 (0.91, 1.37)	0.93 (0.76, 1.14)	1.01 (0.82, 1.23)	1.02 (0.77, 1.34)
Central obesity (yes vs no)1	1.03 (0.83, 1.27)	1.29 (0.86, 1.93)	1.00 (0.70, 1.45)	1.01 (0.70, 1.45)	0.98 (0.79, 1.22)	0.93 (0.68, 1.27)	1.02 (0.71, 1.47)	**0.78 (0.62, 0.97)**	1.24 (0.99, 1.56)	0.98 (0.79, 1.22)	0.94 (0.75, 1.16)	0.95 (0.71, 1.28)

Adjust for age, sex and region (where appropriate). Bold values denote statistical significance at the $p < 0.05$ level.
1 Central obesity is defined as a waist circumference greater or equal to 94 cm in men and 80 cm in women.

Table S3. Sero-conversion and sero-reversion rates estimated from 100 individuals with samples available at baseline, 1st resurvey and 2nd resurvey

Pathogen	Baseline - 1st resurvey		Baseline - 2nd resurvey		1st resurvey - 2nd resurvey	
	Sero-conversion (%)	Sero-reversion (%)	Sero-conversion (%)	Sero-reversion (%)	Sero-conversion (%)	Sero-reversion (%)
**Hepatitis viruses**						
HBV	2 (2.1)	2 (2.1)	2 (2.0)	2 (2.0)	4 (4.1)	4 (4.1)
HCV	0 (0)	1 (1.0)	0 (0)	1 (1.0)	0 (0)	0 (0)
**Human papillomaviruses (HPV)**						
HPV-16	1 (1.0)	4 (4.1)	1 (1.0)	4 (4.1)	3 (3.1)	3 (3.1)
HPV-18	0 (0)	2 (2.1)	1 (1.0)	1 (1.0)	1 (1.0)	1 (1.0)
**Human herpesviruses (HHV)**						
HHV-1	0 (0)	0 (0)	0 (0)	0 (0)	0 (0)	0 (0)
HHV-2	3 (3.1)	0 (0)	4 (4.1)	0 (0)	1 (1.0)	0 (0)
VZV	5 (5.2)	4 (4.1)	3 (3.1)	2 (2.0)	3 (3.1)	3 (3.1)
EBV	0 (0)	0 (0)	0 (0)	0 (0)	0 (0)	0 (0)
CMV	1 (1.0)	0 (0)	1 (1.0)	0 (0)	0 (0)	0 (0)
HHV-6	11 (11.3)	11 (11.3)	8 (8.2)	12 (12.2)	8 (8.2)	11 (11.2)
HHV-7	7 (7.2)	10 (10.3)	8 (8.2)	5 (5.1)	11 (11.2)	4 (4.1)
**Human polyomaviruses (HPyV)**						
BKV	2 (2.1)	7 (7.2)	4 (4.1)	4 (4.1)	8 (8.2)	2 (2.0)
JCV	2 (2.1)	3 (3.1)	2 (2.0)	3 (3.1)	3 (3.1)	3 (3.1)
MCV	2 (2.1)	5 (5.2)	3 (3.1)	6 (6.1)	2 (2.0)	3 (3.1)
**Other viruses**						
HIV	0 (0)	0 (0)	0 (0)	0 (0)	0 (0)	0 (0)
HTLV	0 (0)	0 (0)	0 (0)	0 (0)	0 (0)	0 (0)
**Bacteria/Parasite**						
H. pylori	2 (2.1)	4 (4.1)	3 (3.1)	12 (12.2)	3 (3.1)	10 (10.2)
C. trachomatis	3 (3.1)	2 (2.1)	4 (4.1)	1 (1.0)	2 (2.0)	0 (0)
T. gondii	4 (4.1)	8 (8.2)	3 (3.1)	6 (6.1)	7 (7.1)	6 (6.1)

Sero-conversion referred to the process of having "become pathogen positive", and sero-reversion is the opposite of seroconversion

Table S4. Age-standardized sero-prevalence (%) for infectious pathogens in CKB and UKB

Pathogens	CKB	UKB
**Hepatitis virus**		
HBV[1]	41.4	2.6
HCV[1]	0.6	0.3
**Human Papillomavirus (HPV)**		
HPV-16[1]	5.8	4.9
HPV-18[1]	3.2	3.0
**Human Herpesvirus (HHV)**		
HHV-1	93.4	68.6
HHV-2	8.3	16.8
VZV	87.1	92.2
EBV[1]	95.1	94.8
CMV	92.7	55.5
HHV-6	59.7	90.8
HHV-7	81.6	95.0
**Human Polyomavirus (HPyV)**		
BKV	86.9	95.9
JCV	61.5	62.4
MCV	60.9	67.3
**Other viruses**		
HIV[1]	0.04	0.2
HTLV[1]	0.1	1.4
**Bacteria/Parasite**		
H. pylori[1]	50.5	34.1
C. trachomatis	44.1	23.1
T. gondii	13.1	26.7
**Number of any pathogen +**	**9.3 (1.7)**	**8.4 (1.9)**
**Number of oncogenic pathogens +**	**2.1 (0.8)**	**1.4 (0.7)**

Sero-prevalence for each infectious pathogen in CKB and UKB was directly standardized to WHO World Standard Population (available from https://seer.cancer.gov/stdpopulations/world.who.html)

[1] Oncogenic pathogen

Yao P, *et al. BMJ Open* 2022; 12:e058353. doi: 10.1136/bmjopen-2021-058353

Figure S1. Locations of the 10 survey sites and number recruited.

Open circles indicate rural areas and solid circles indicate urban areas. Number recruited at baseline in each area is shown in brackets.

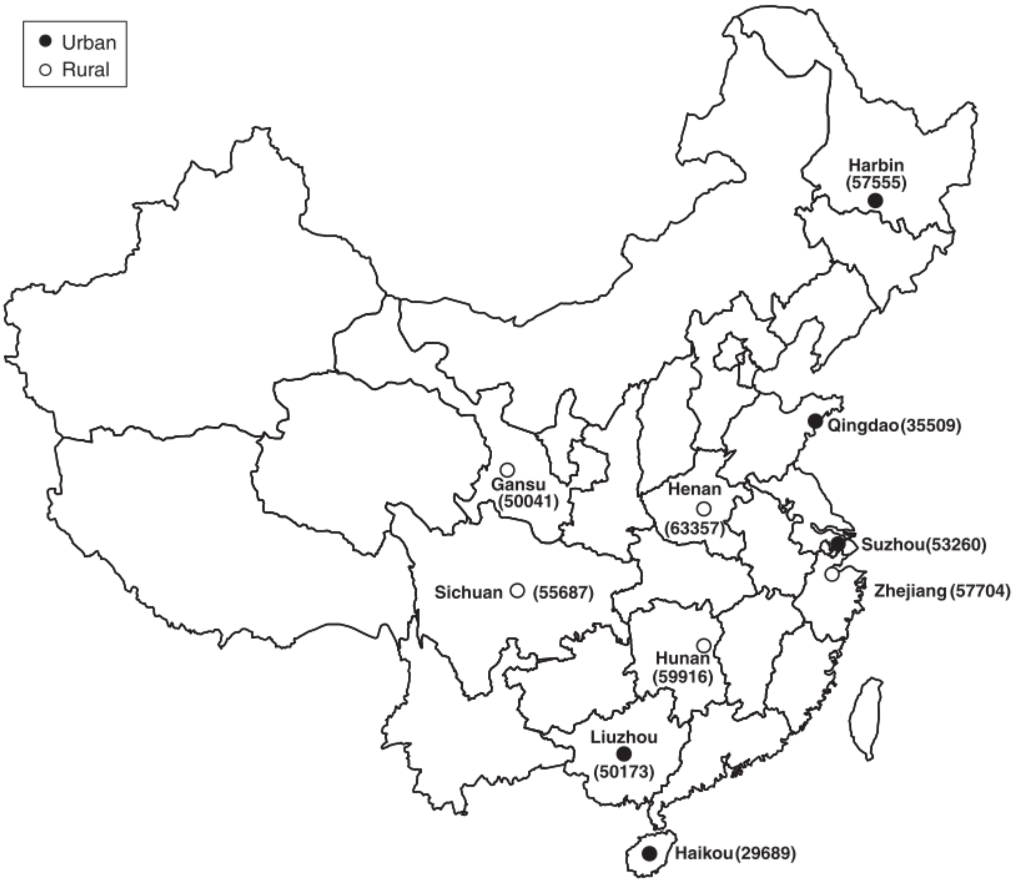

Figure S2. Distribution of median fluorescence intensity of 43 antigens

For each antigen, orange and blue represent sero-status of positive and negative, respectively.

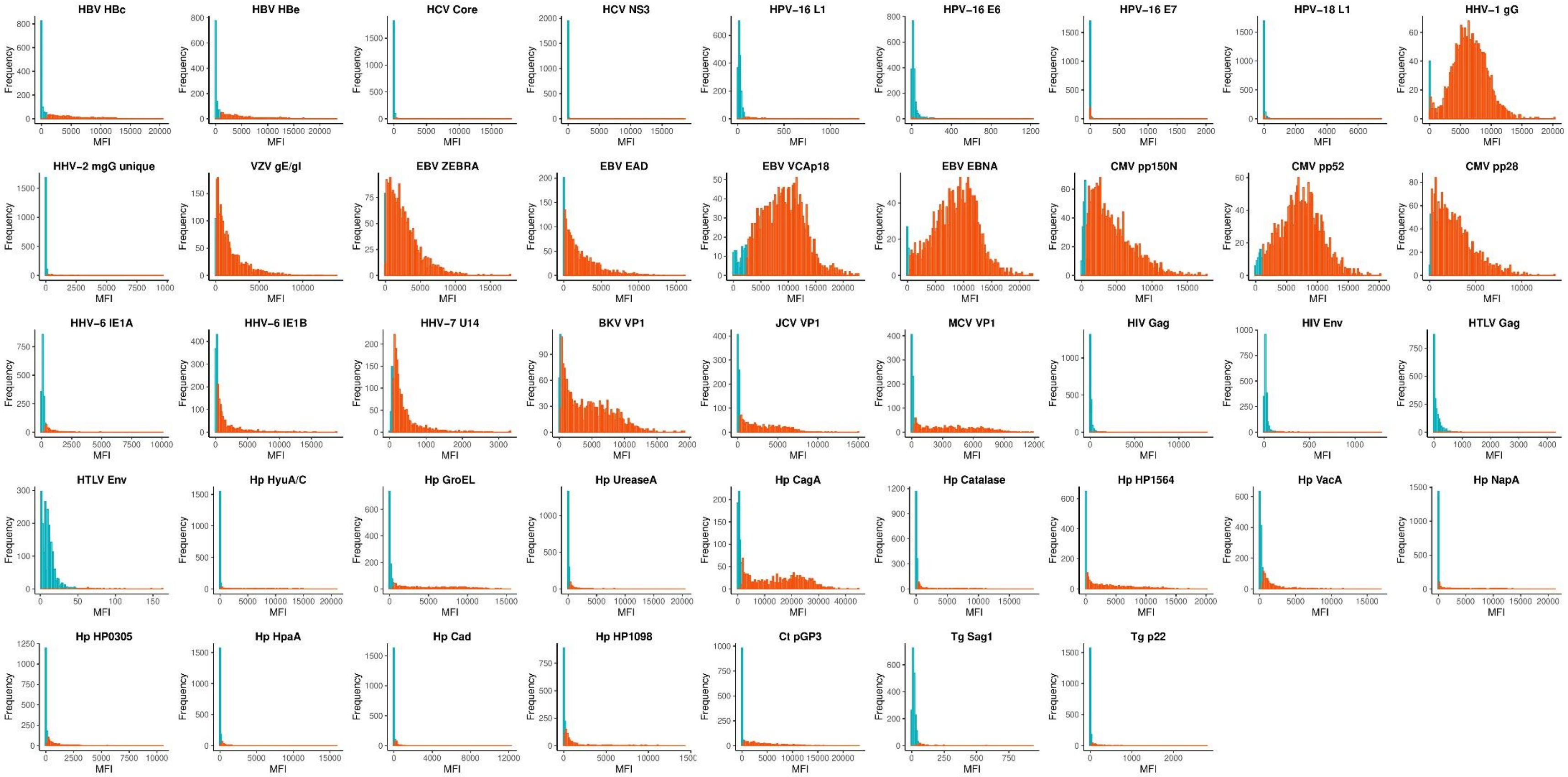

Yao P, *et al. BMJ Open* 2022; 12:e058353. doi: 10.1136/bmjopen-2021-058353

Figure S3. Correlation matrix of pathogens

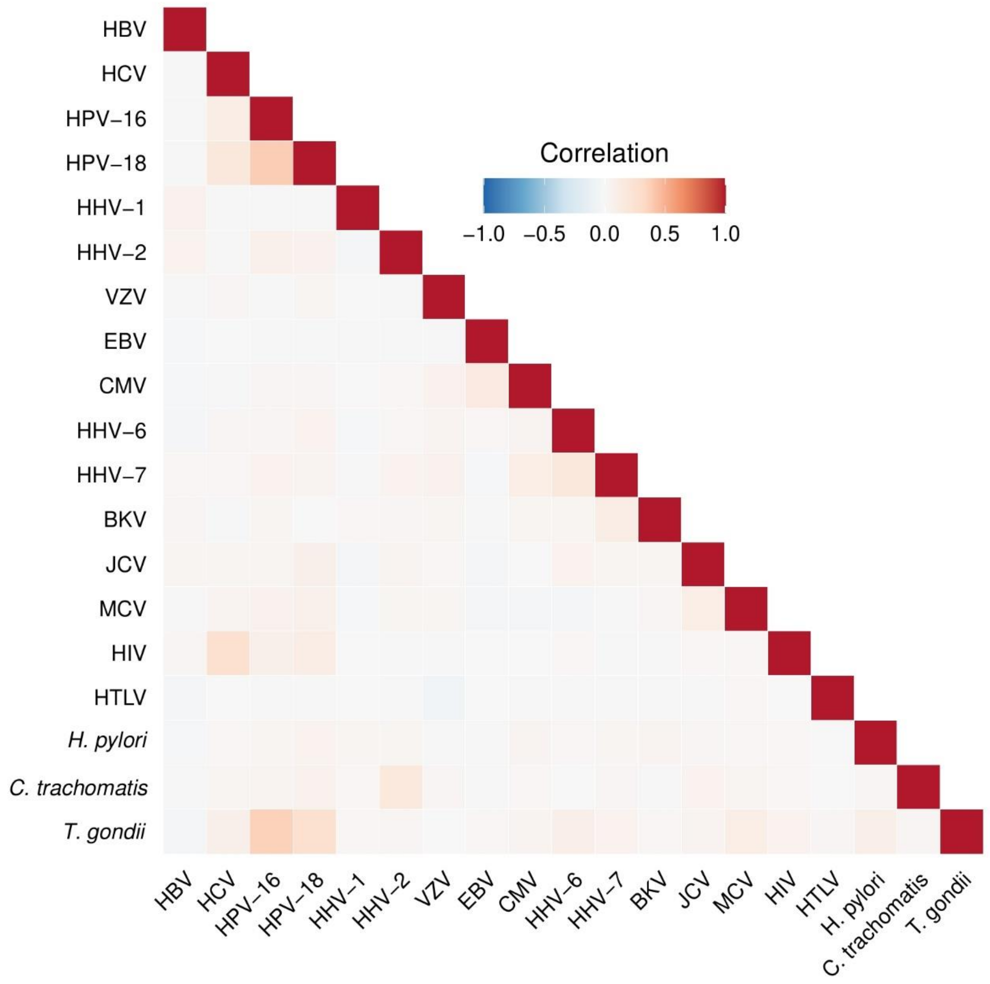

Figure S4. Correlation matrix of 43 antigens

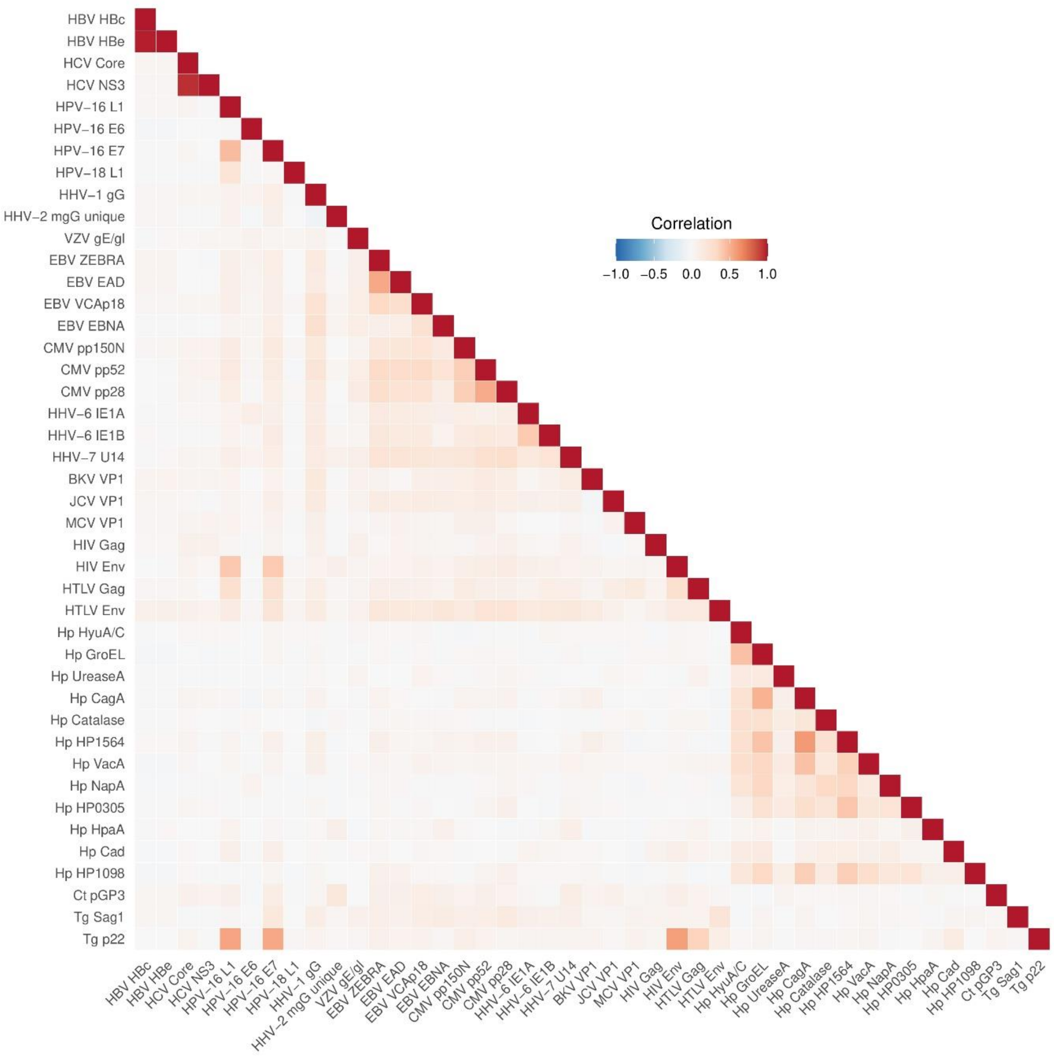

Figure S5. Intraclass correlation coefficient for MFI of 43 antigens between baseline and the 1st resurvey number of infections

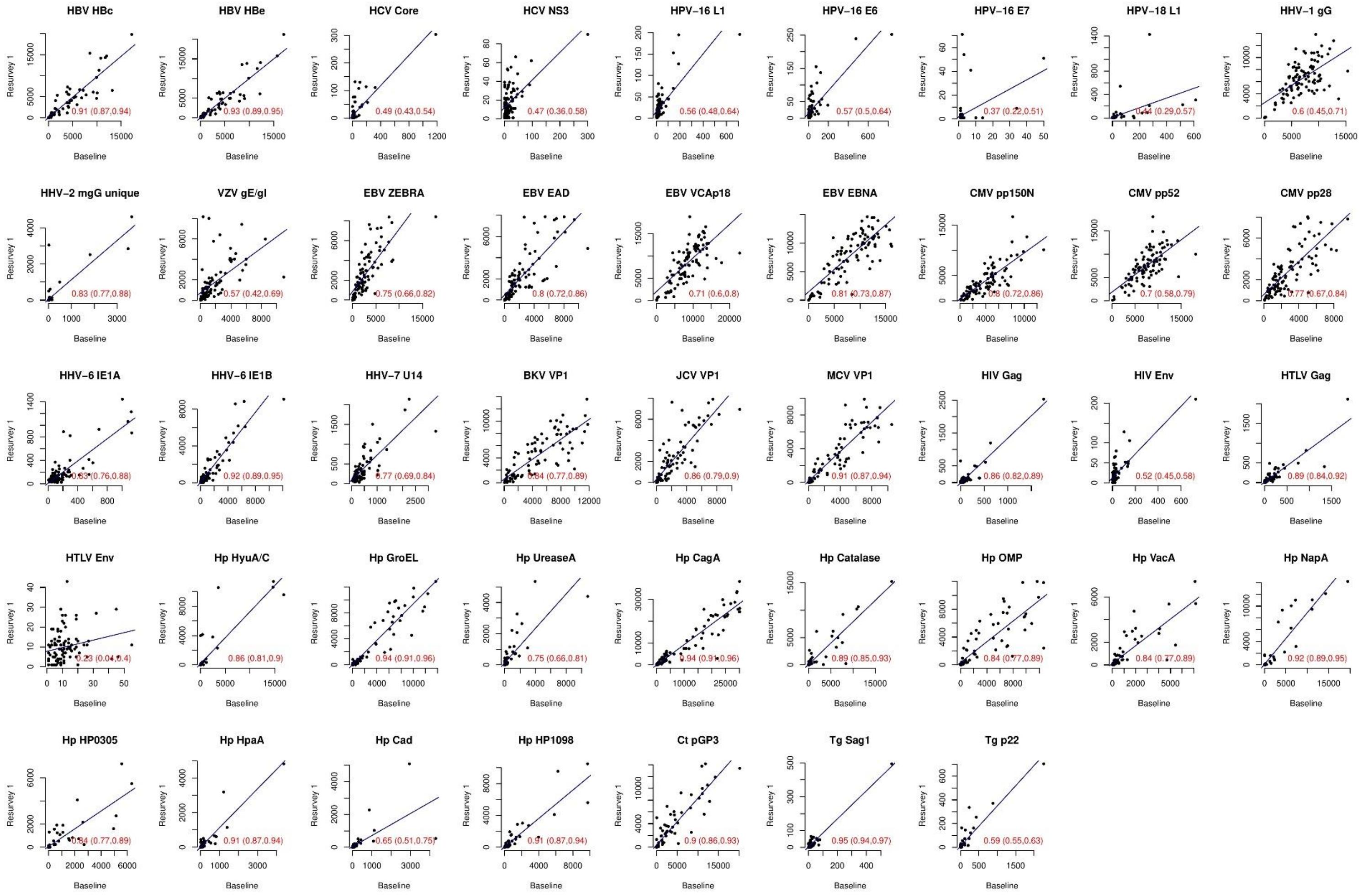

Figure S6. Intraclass correlation coefficient for MFI of 43 antigens between baseline and the 2nd resurvey

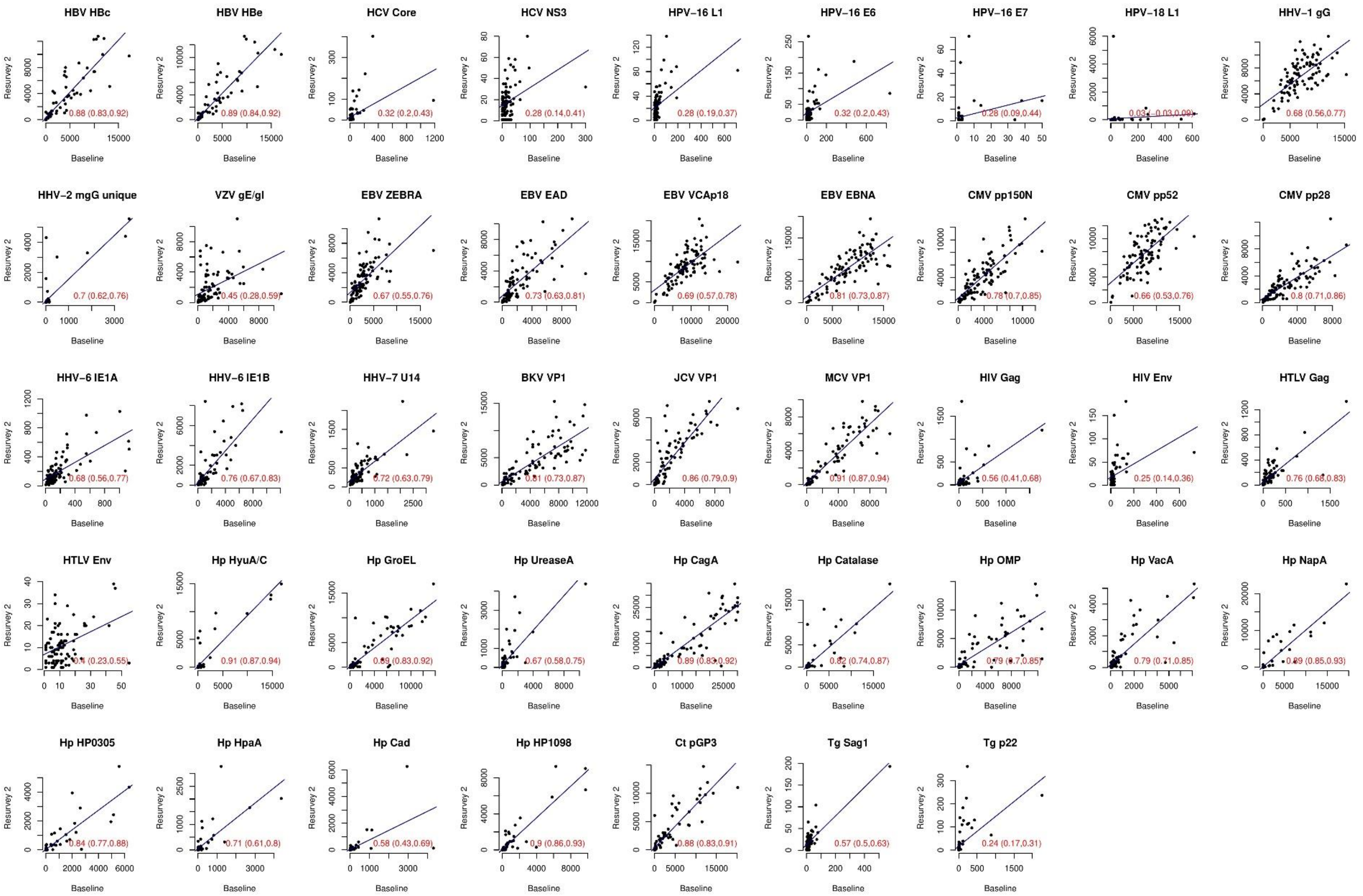

Figure S7. Intraclass correlation coefficient for MFI of 43 antigens between the 1st and the 2nd resurvey

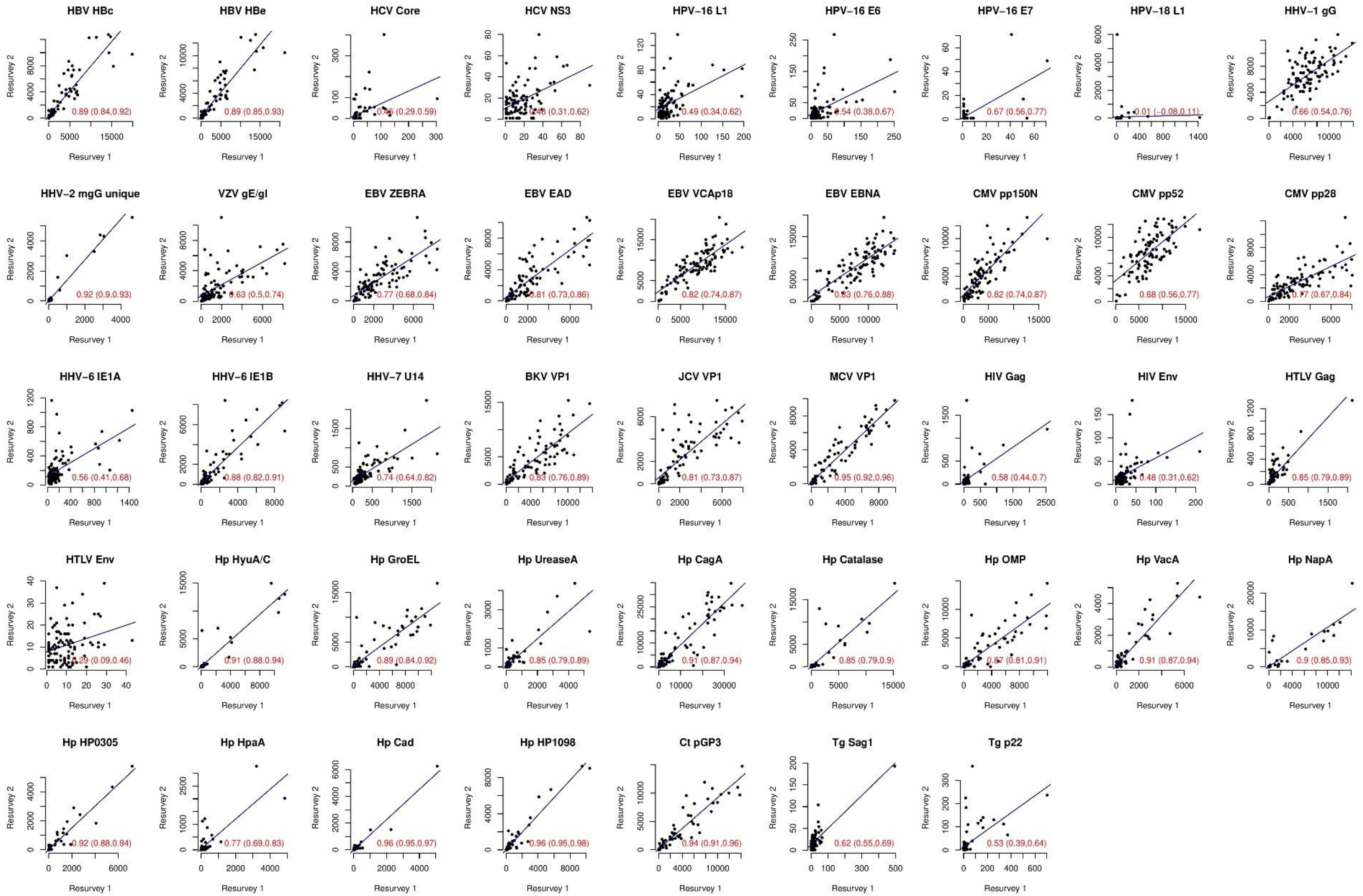

Yao P, *et al. BMJ Open* 2022; 12:e058353. doi: 10.1136/bmjopen-2021-058353
