# Supplementary material for: Sero-prevalence of 19 infectious pathogens and associated factors among middle-aged and elderly Chinese adults: a cross-sectional study
Source: BMJ Open. 2022 May 9;12(5):e058353. doi: 10.1136/bmjopen-2021-058353 (PMC9086621; doi:10.1136/bmjopen-2021-058353)
Supplement: Author's manuscript [file bmjopen-2021-058353.draft_revisions.pdf]

BMJ Open is committed to open peer review. As part of this commitment we make the peer review history of every article we publish publicly available.

When an article is published we post the peer reviewers' comments and the authors' responses online. We also post the versions of the paper that were used during peer review. These are the versions that the peer review comments apply to.

The versions of the paper that follow are the versions that were submitted during the peer review process. They are not the versions of record or the final published versions. They should not be cited or distributed as the published version of this manuscript.

BMJ Open is an open access journal and the full, final, typeset and author-corrected version of record of the manuscript is available on our site with no access controls, subscription charges or pay-per-view fees (http://bmjopen.bmj.com).

If you have any questions on BMJ Open's open peer review process please email info.bmjopen@bmj.com

BMJ Open

Sero-prevalence of 19 infectious pathogens and associated factors among adults from 10 urban and rural areas of China

Journal:	*BMJ Open*
Manuscript ID	bmjopen-2021-058353
Article Type:	Original research
Date Submitted by the Author:	13-Oct-2021
Complete List of Authors:	Yao, Pang; University of Oxford Nuffield Department of Population Health; University of Oxford Nuffield Department of Population Health, University of Oxford<br>Millwood, Iona; University of Oxford, Nuffield Department of Population Health; University of Oxford, Nuffield Department of Population Health<br>Kartsonaki, Christiana ; University of Oxford, Nuffield Department of Population Health<br>Mentzer, Alexander J; University of Oxford<br>Allen, Naomi; University of Oxford, Nuffield Department of Population Health<br>Jeske, Rima; Infections and Cancer Epidemiology Division, German Cancer Research Center<br>Butt, Julia; Division of Molecular Diagnostics of Oncogenic Infections, Infection, Inflammation and Cancer Program, German Cancer Research Center (DKFZ)<br>Guo, Yu; Chinese Academy of Medical Sciences<br>Chen, Yiping; University of Oxford, Nuffield Department of Population Health<br>Walters , Robin; University of Oxford, Nuffield Department of Population Health; University of Oxford, Nuffield Department of Population Health<br>Lv, Jun; Peking University Health Science Center, Epidemiology & Biostatistics<br>Yu, Canqing; Peking University Health Science Center, Epidemiology & Biostatistics<br>Plummer, Martyn; University of Warwick, Department of Statistics<br>de Martel, Catherine; International Agency for Research on Cancer<br>Clifford, Gary; International Agency for Research on Cancer<br>LI, Li-ming; Peking University School of Public Health, Epidemiology and Biostatistics<br>Waterboer, Tim; Division of Molecular Diagnostics of Oncogenic Infections, Infection, Inflammation and Cancer Program, German Cancer Research Center (DKFZ)<br>Yang, Ling; University of Oxford, Nuffield Department of Population Health; University of Oxford, Nuffield Department of Population Health<br>Chen, Zhengming; University of Oxford, Nuffield Department of Population Health
Keywords:	Epidemiology < INFECTIOUS DISEASES, PUBLIC HEALTH, INFECTIOUS DISEASES

SCHOLARONE™
Manuscripts

I, the Submitting Author has the right to grant and does grant on behalf of all authors of the Work (as defined in the below author licence), an exclusive licence and/or a non-exclusive licence for contributions from authors who are: i) UK Crown employees; ii) where BMJ has agreed a CC-BY licence shall apply, and/or iii) in accordance with the terms applicable for US Federal Government officers or employees acting as part of their official duties; on a worldwide, perpetual, irrevocable, royalty-free basis to BMJ Publishing Group Ltd ("BMJ") its licensees and where the relevant Journal is co-owned by BMJ to the co-owners of the Journal, to publish the Work in this journal and any other BMJ products and to exploit all rights, as set out in our licence.

The Submitting Author accepts and understands that any supply made under these terms is made by BMJ to the Submitting Author unless you are acting as an employee on behalf of your employer or a postgraduate student of an affiliated institution which is paying any applicable article publishing charge ("APC") for Open Access articles. Where the Submitting Author wishes to make the Work available on an Open Access basis (and intends to pay the relevant APC), the terms of reuse of such Open Access shall be governed by a Creative Commons licence – details of these licences and which Creative Commons licence will apply to this Work are set out in our licence referred to above.

Other than as permitted in any relevant BMJ Author's Self Archiving Policies, I confirm this Work has not been accepted for publication elsewhere, is not being considered for publication elsewhere and does not duplicate material already published. I confirm all authors consent to publication of this Work and authorise the granting of this licence.

For peer review only

Sero-prevalence of 19 infectious pathogens and associated factors among adults from 10 urban and rural areas of China

Pang Yao[1], Iona Y. Millwood[1,2,] Christiana Kartsonaki[1,2], Alexander J Mentzer[3], Naomi Allen[1], Rima Jeske[4], Julia Butt[4], Yu Guo[5], Yiping Chen[1,2], Robin G. Walters[1,2], Jun Lv[6], Canqing Yu[6], Martyn Plummer[7], Catherine de Martel[8], Gary M. Clifford[8], Liming Li[6], Tim Waterboer[4, #], Ling Yang[1,2, #], Zhengming Chen[1, #]

1 Clinical Trial Service Unit & Epidemiological Studies Unit (CTSU), Nuffield Department of Population Health, University of Oxford, Oxford, UK
2 Medical Research Council Population Health Research Unit (MRC PHRU), Nuffield Department of Population Health, University of Oxford, Oxford, UK
3 The Wellcome Centre for Human Genetics, University of Oxford, Oxford, UK
4 Infections and Cancer Epidemiology Division, German Cancer Research Center (DKFZ), Heidelberg, Germany
5 Fuwai Hospital Chinese Academy of Medical Sciences, National Center for Cardiovascular Diseases, Beijing, China
6 Department of Epidemiology and Biostatistics, School of Public Health, Peking University Health Science Centre, Beijing, China
7 Department of Statistics, University of Warwick, Coventry, UK
8 Early Detection, Prevention and Infections Branch, International Agency for Research on Cancer, Lyon, France
\# *co-senior authors*

Address for correspondence:

Ling Yang
MRC PHRU and CTSU
Nuffield Department of Population Health, Big Data Institute Building, University of Oxford,
Old Road Campus, Oxford, OX3 7LF, United Kingdom
Email: ling.yang@ndph.ox.ac.uk
Tel: +44 (0) 1865 743936

or

Iona Y Millwood
MRC PHRU and CTSU
Nuffield Department of Population Health, Big Data Institute Building, University of Oxford,
Old Road Campus, Oxford, OX3 7LF, United Kingdom
Email: iona.millwood@ndph.ox.ac.uk
Tel: +44 (0) 1865 743612

Word count: Abstract 261, Text 3624
2 Tables 3 Figures and 11 Supplementary Tables or Supplementary Figures
CKB Research Tracker No.: 2019-0052

13 October 2021

For peer review only - http://bmjopen.bmj.com/site/about/guidelines.xhtml

Abstract

Objectives: To systematically assess the sero-prevalence and associated factors of major infectious pathogens in China, where there are high incidence rates of certain infection-related cancers.

Design: Cross-sectional survey.

Methods: In the China Kadoorie Biobank of >512,000 adults recruited during 2004-2008, plasma samples from a random subcohort of 2000 participants were assayed, using a custom-designed multiplex serology panel, for antibody levels against 19 pathogens. The sero-prevalence of each pathogen was estimated and their associated factors were assessed using logistic regression.

Results: Of the 19 pathogens investigated, the mean number of sero-positive pathogens was 9.4 (SD 1.7), with 24.4% of participants being sero-positive for >10 pathogens. For individual pathogens, the sero-prevalence varied, being e.g. 0.05% for HIV, 6.4% for HPV-16, 53.5% for *H. pylori* and 99.8% for EBV. The sero-prevalence of HHV-6, HHV-7 and HPV-16 was higher in women than men. Several pathogens showed a decreasing trend in sero-prevalence by birth cohort, including HBV (51.6% vs 38.7% in those born <1940 vs >1970), HPV-16 (11.4% vs 5.4%), HHV-2 (15.1% vs 8.1%), *C. trachomatis* (65.6% vs 28.8%) and *T. gondii* (22.0% vs 9.0%). Across the 10 study areas, sero-prevalence varied 2 to 4-fold for HBV (22.5% to 60.7%), HPV-16 (3.4% to 10.9%), *H. pylori* (16.2% to 71.1%) and *C. trachomatis* (32.5% to 66.5%). Participants with chronic liver diseases had >7-fold higher sero-positivity for HBV (OR=7.51; 95% CI: 2.55-22.13).

Conclusions: Among Chinese adults, past and current infections with certain pathogens were common, and varied by area, sex and birth cohort. These infections may contribute to increased risk of certain cancers and other non-communicable chronic diseases.

For peer review only - http://bmjopen.bmj.com/site/about/guidelines.xhtml

Strengths and limitations of this study

The present study is the simultaneous assessment of antibodies against multiple pathogens, using a validated and custom designed multiplex serology platform.

The coverage of ten different areas in China enabled investigation of geographic variations in the sero-prevalence of pathogens, in addition to associations with socioeconomic and lifestyle factors.

The broad age range of participants also allowed assessment of differences in the pathogens' sero-prevalence across the different birth cohorts and/or age groups.

The multiplex serology measurements are designed to detect cumulative (i.e. past and present) exposure in a high-throughput setting and may be less sensitive or specific than assays used in clinical diagnosis.

The information collected about possible determinants or correlates for certain infections was limited, particularly in relation to sexual behaviours.

Data availability statement

No data are available.

For peer review only - http://bmjopen.bmj.com/site/about/guidelines.xhtml

Introduction

Chronic infections with certain pathogens, including viruses, bacteria, and parasites, have been associated with increased risks of several non-communicable chronic diseases (NCDs), especially certain cancers.[1] In 2018, an estimated 2.2 million cancer cases worldwide were attributed to infections, mainly *Helicobacter pylori* (*H. pylori*; 810,000 cases), human papillomavirus (HPV; 690,000), hepatitis B virus (HBV; 360,000) and hepatitis C virus (HCV; 160,000).[1] The incidence rates of infection-related cancers were highest in eastern Asia, particularly China, owing to the high prevalence of *H. pylori* and HBV.[1] Apart from cancer, a growing body of evidence also suggests that inflammation induced by persistent pathogen infection may be associated with risks of cardiovascular disease (cytomegalovirus),[2] multiple sclerosis (Epstein-Barr virus [EBV])[3] and Alzheimer's disease (human herpesviruses [HHV] 6 and 7).[4] Moreover, there is also evidence suggesting that pathogen burden (i.e., the number of different infectious pathogens an individual has been exposed to) may contribute to atherosclerosis and risk of cardiovascular disease.[2]

The prevalence of pathogens tends to vary greatly between and within populations and is often associated with disparities in socioeconomic status, household crowding, breastfeeding practices, food-production practices and environmental factors such as climate.[5] Although many sero-epidemiological studies of major infectious pathogens have been undertaken,[6-9] they have tended to cover a single pathogen,[6-8] or involve populations in high-income countries.[9] In China, although several nationwide surveys have previously reported on the prevalence of some major pathogens (e.g. HBV, HCV),[10-12] there is limited contemporary data about the sero-prevalence of many other pathogens and their relevance for major NCDs beyond certain cancers.

For peer review only - http://bmjopen.bmj.com/site/about/guidelines.xhtml

Moreover, few studies in China or elsewhere have systematically examined the sero-prevalence of multiple pathogens in the same population, which could provide important data about pathogen burden and the joint effects of different pathogens on disease aetiology.

The present study examined the sero-prevalence of 19 infectious pathogens and investigated certain demographic and environmental factors associated with sero-positivity among 2000 adults from the China Kadoorie Biobank (CKB) study that covered 10 geographically diverse urban and rural areas in China.

Materials and Methods

Study population

Details of the CKB study design, methods, and participants have been previously reported.[13] The 512,715 participants aged 30-79 years were enrolled from 10 (5 urban, 5 rural) geographically diverse regions in China (**Supplementary Figure 1**). Ethics approval was obtained from the Oxford University, the China National CDC, and also from institutional research boards at the local CDCs in the 10 areas.[13] All participants provided written informed consent. The present study population was a subcohort of 2000 participants from a case-subcohort study of gastric and oesophageal cancers in CKB. This subcohort was selected using simple random sampling from those alive with no history of cancer 2 years after entering the study who had an available plasma sample collected at baseline and had genotyping data available as part of a population-based subset.

Patient and public involvement

No patients or population groups were involved.

For peer review only - http://bmjopen.bmj.com/site/about/guidelines.xhtml

Blood sample collection and processing

At baseline, a 10 ml non-fasting venous blood sample was collected from each participant into an EDTA vacutainer (with time of last meal recorded). Samples were separated into plasma and buffy coat aliquots, and frozen within 24 hours of collection (mean delay ~10 hours). Two aliquots of plasma from each blood sample were shipped to Oxford, UK for long-term storage in liquid nitrogen following the formal approval of regulatory agencies in China.

Multiplex serology

The multiplex serology panel used in the present study was based on that developed for the UK Biobank,[9] with certain relevant modifications. More *H. pylori* antigens were included in the CKB panel and a few pathogens were removed taking their prevalence into account.[9] The CKB panel measured antibody levels against 43 antigens from 19 infectious pathogens (see details in Supplementary Table 1) including hepatitis B (HBV) and hepatitis C (HCV) viruses; human papillomaviruses 16 (HPV-16) and 18 (HPV-18); herpes simplex viruses 1 and 2 (HHV-1 and HHV-2); varicella zoster virus (VZV); Epstein-Barr virus (EBV); human cytomegalovirus (CMV); human herpesviruses 6 and 7 (HHV-6 and HHV-7); BK (BKV), JC (JCV) and Merkel cell (MCV) polyomaviruses; human immunodeficiency virus (HIV); human T lymphotropic virus (HTLV); *Helicobacter pylori (H. pylori)*; *Chlamydia trachomatis (C. trachomatis)*; and *Toxoplasma gondii (T. gondii)*. Of these 19 pathogens, 8 (HBV, HCV, HIV, HTLV, *H. pylori*, HPV-16, HPV-18 and EBV) are classified as Group I human carcinogens by the IARC.

The quantitative multiplex antibody detection approach was based on a glutathione S-transferase (GST) capture enzyme-linked immunosorbent assay combined with

For peer review only - http://bmjopen.bmj.com/site/about/guidelines.xhtml

For peer review only

fluorescent-bead technology, with various validation procedures.[14] Antigens of infectious pathogens were bacterially expressed as GST-X-tag fusion proteins and coupled to spectrally distinguishable glutathione-casein covered beads. The median fluorescence intensity (MFI) of at least 100 beads per antigen was measured, and thus a sample was defined sero-positive for specific antibody against a corresponding antigen if the respective MFI value exceeded the antigen-specific cut-off. To define overall sero-positivity for a pathogen, antigen-specific sero-positivity results were subsequently combined using published algorithms (see details in Supplementary Table 1 and Supplementary Figure 2).

Statistical analysis

Sero-prevalence estimates and 95% confidence intervals (CIs) for each antigen and pathogen were calculated. For pathogens, sero-prevalence was also estimated in subgroups defined by sex, birth cohort (each year) and study area (10). Locally weighted scatterplot smoothing (LOESS) curves were used to visualise the relationship between birth cohort and sero-prevalence of each pathogen, and proportion of individuals with >10 infectious pathogens, or >2 oncogenic pathogens. Logistic regression was used to estimate odds ratios (ORs) by pathogen sero-positivity for various baseline characteristics, adjusting for age, sex and area. Pathogens with extreme sero-prevalence (i.e., <5% or >95%) were excluded from the association analyses. Continuous MFI values were used to compute Pearson correlation coefficients between each of the antigens. Spearman's correlation coefficients were calculated between pathogens. The analysis was done using R version 3.6.2.

For peer review only - http://bmjopen.bmj.com/site/about/guidelines.xhtml

Results

Characteristics of the study population

Among the subcohort of 1986 participants (14 samples were excluded due to technical errors) included in the main analyses, the mean (SD) age was 51.8 (10.8) years, 61.7% were women, and 50.3% were urban residents, which, along with many other baseline characteristics, were similar to those in the overall CKB cohort (**Table 1**).

Sero-prevalence of pathogens

Of the 19 pathogens investigated, the sero-prevalence estimates ranged from 0.05% to 99.8% for individual pathogens (**Table 2**). Within specific families of pathogens, the sero-prevalences also varied greatly. For the hepatitis viruses, the sero-prevalence was 43.8% for HBV and 0.7% for HCV. Among papillomaviruses, HPV-16 sero-prevalence was higher (6.4%) than HPV-18 (3.4%). The sero-prevalence of herpesviruses was 98.0% for HHV-1, 8.9% for HHV-2, 91.5% for VZV, 99.8% for EBV, 97.3% for CMV, 62.0% for HHV-6, and 85.5% for HHV-7. Among the polyomaviruses, BKV had the highest sero-prevalence (91.1%), followed by JCV (65.6%) and MCV (64.9%). For HIV and HTLV, however, only samples from 1 (0.05%) and 2 (0.1%) participants tested positive, respectively. The sero-prevalences of *H. pylori, C. trachomatis,* and *T. gondii* were 53.5%, 47.6%, and 14.2% respectively.

No differences by sex were observed for most pathogens but, compared with men, women had a higher sero-prevalence of HPV-16 (7.9% vs 4.1%), HHV-6 (68.0% vs 52.3%) and HHV-7 (89.3% vs 79.5%). A decreasing trend in sero-prevalence by birth

For peer review only

For peer review only - http://bmjopen.bmj.com/site/about/guidelines.xhtml

cohort was seen for HBV (51.6% in those born before 1940 to 38.7% in those born after 1970), HPV-16 (11.4% to 5.4%), HHV-2 (15.1% to 8.1%), JCV (75.5% to 61.3%), MCV (73.6% to 62.2%), *C. trachomatis* (65.6% to 28.8%), and *T. gondii* (22.0% to 9.0%), while no clear trends were evident for other pathogens, except HHV-6 which showed an upward trend (**Figure 1**). Across the 10 study areas, there were at least 2-fold differences in sero-prevalence for HBV (22.5% to 60.7%), HPV-16 (3.4% to 10.9%), HHV-2 (4.3% to 17.8%), *H. pylori* (16.2% to 71.1%), *C. trachomatis* (32.5% to 66.5%) and *T. gondii* (8.0% to 18.4%) (**Figure 2**).

Among the 1986 participants and 19 pathogens tested, all participants were sero-positive for at least 1 pathogen and 24.4% were sero-positive for more than 10 pathogens, with the mean number being 9.4 (SD, 1.7) (**Figure 3**). The mean number of oncogenic pathogens for which a participant was sero-positive was 2.1 (SD, 0.8) out of 8 oncogenic pathogens. Overall, there was a clear decreasing trend by birth cohort in the proportions of participants sero-positive for >10 pathogens, or >2 oncogenic pathogens (**Figure 3**).

Factors associated with pathogen sero-positivity

The associations between sero-positivity of three major oncogenic pathogens (*H. pylori*, HPV-16, and HBV) and demographic and lifestyle factors are shown in **Supplementary Table 2**. Sero-positivity for HBV was more common in older participants (P_{trend} =0.05), those with cirrhosis or chronic hepatitis (adjusted OR=7.51, 2.55-22.13), and those who were hepatitis B surface antigen (HBsAg; measured using a point-of-care test) positive (11.51, 5.16-25.7) at baseline. The factors significantly associated with HPV-16 infection were older age (P_{trend} =0.02), being female (2.07, 1.36-3.14), higher levels of education (1.63, 1.01-2.65) or low levels of

For peer review only - http://bmjopen.bmj.com/site/about/guidelines.xhtml

physical activity (0.50, 0.32-0.78), having diabetes (2.69, 1.52-4.77) and obesity/overweight status (1.51, 1.03-2.22). For *H. pylori*, urban residence was associated with higher sero-prevalence (1.80, 1.50-2.15), and the converse was observed for a history of peptic ulcer (0.52, 0.32-0.85).

For the human herpesvirus family, women were more likely to be sero-positive for HHV-2 (1.45, 1.04-2.03), HHV-6 (1.92, 1.59-2.31), and HHV-7 (2.15, 1.67-2.76) than men. Higher physical activity levels were associated with lower likelihood of VZV sero-positivity while participants who reported self-rated poor health were less likely to be HHV-7 sero-positive. For JCV, diabetes and central obesity were associated with lower likelihood of sero-positivity, while for *C. trachomatis*, higher levels of education and income were associated with lower, and diabetes was associated with higher sero-prevalence.

Correlation between different pathogens

Correlations between the 19 different pathogens were generally low (**Supplementary Figure 3**). However, for certain pathogens, there were high correlations between their different antigens, e.g. HBc and HBe (r=0.99) for HBV; core antigen and NS3 (r=0.95) for HCV, and HP1564 and CagA (r=0.60) for *H. pylori* (**Supplementary Figure 4**). For certain different pathogens, moderate correlations were also observed between certain antigens, such as HPV-16 L1 and *T. gondii* p22 (r=0.56).

Discussion

In this large study of multiple pathogens in the general adult Chinese population, there were large differences in the sero-prevalence of different pathogens, both overall and by sex, birth cohort and areas. Moreover, certain socioeconomic factors

For peer review only - http://bmjopen.bmj.com/site/about/guidelines.xhtml

For peer review only

(e.g. levels of education and income), lifestyle factors (e.g. physical activity) and medical history (e.g., having cirrhosis or chronic hepatitis) were also shown to be associated with sero-prevalence of many pathogens. Among the study population and the 19 pathogens tested, the mean number of sero-positive pathogens was more than 9.

The present study shows there is a substantial burden of past and current infections with various types of pathogens among Chinese adults. Compared with data from a similar study in the UK population involving about 10,000 adults (56% women),[9] certain infections were more prevalent in Chinese adults, particularly HBV, HHV-1, CMV, *H. pylori* and *C. trachomatis*, while others were less common, including HHV-2, HHV-6, HHV-7 and *T. gondii* (**Supplementary Table 4**). Overall, the mean number of sero-positive pathogens was slightly greater in Chinese than in UK adults (any pathogen 9.4 vs 8.4; oncogenic pathogens: 2.1 vs 1.4), suggesting that the overall burden of pathogen infections might be generally higher in Chinese populations.

HBV and HCV

For HBV, we measured antibody responses for HBc and HBe which indicate past and current natural HBV infection, respectively, and the rate was approximately 44% in the present study, much higher than the 3% reported in the UK Biobank.[9] Our sero-prevalence estimate was, however, lower than recent data (54.7%) from a much smaller study (214 controls in a nested case-control study, samples obtained from 1996-2000) in China and Singapore using the same antigens (HBc and HBe) measured using multiplex serology.[16] Several Chinese nationwide surveys indicated that the prevalence of chronic HBV infection among adults measured by hepatitis B surface antigen (HBsAg) had decreased steadily over the past two decades, from

For peer review only - http://bmjopen.bmj.com/site/about/guidelines.xhtml

10% in 1992,[10] to 7% in 2006,[11] and to 6% in 2010-2012.[12] In line with previous findings,[10-12] we also observed a declining sero-prevalence of HBV across birth cohorts, which was not influenced by the universal HBV vaccination program that was implemented about 20 years ago and restricted to new-borns. Our finding of a sero-prevalence of HCV (0.7%) is consistent with recent report from China using a similar assay (0.9%) based on a smaller sample size (n=214),[16] and higher than the 0.3% sero-prevalence in the UK Biobank.[9] Our study showed that participants with reported prior cirrhosis or chronic hepatitis had >7-fold higher risk of HBV sero-positivity. This is not surprising, since both HBV and HCV can lead to progressive liver cirrhosis, fibrosis, and/or hepatocellular carcinoma and thus, have been classified as group I human carcinogens for hepatocellular carcinoma.

HPV-16 and HPV-18

The two most common HPVs, HPV-16 and HPV-18 are sexually transmitted oncogenic viruses, which account for 70-75% of all cervical cancers and 40-60% of cervical cancer precursors.[17] In addition to sex differences, HPV sero-prevalence may be affected by differences in socioeconomic conditions, cultural diversity, assay methods and survey period. In the present study, the sero-prevalences of HPV-16 (overall 6.4%, urban 7.0%, rural 5.9%) and HPV-18 (overall 3.4%, urban 3.5%, rural 3.2%) were comparable to those reported in Chinese living in Taiwan (n=1702; 7.6% and 3.9%, respectively) measured using HPV virus-like particle ELISA,[18] but somewhat lower than those reported in another study of 3790 (median age of 44 years, 58% women) rural Chinese from Central China (9.9% and 8.9%, respectively) measured using multiplex serology.[19] A recent meta-analysis involving 24 Chinese provinces and autonomous regions indicated that HPV-16 and HPV-18 were more

For peer review only - http://bmjopen.bmj.com/site/about/guidelines.xhtml

prevalent in Central and Northwest China than the rest of the country.[20] The increasing trend between HPV-16 sero-prevalence and age was consistent with previous findings,[7 18] which may reflect the fact that cumulative exposure measured by HPV sero-positivity is higher among older individuals and that antibodies to HPV-16 tend to be persistent.[7] As in the present study, many previous studies have consistently reported a higher sero-prevalence of HPV-16 in women than men.[18] It is possible that men are either not as susceptible to HPV-16 infection or more frequently able to clear the infection spontaneously without developing a systemic antibody response. Previous meta-analyses have reported that the overall HPV viral DNA prevalence among Chinese women ranged from 13.1% to 18.8%.[20] Targeted HPV vaccination and scale-up of cervical cancer screening for women are priorities in curbing the HPV epidemic in China.

Human herpesviruses

Herpesviruses are a large family of enveloped DNA viruses that are ubiquitous in humans and characteristically establish lifelong latent infection within host cells and subsequently reactivate under certain conditions. Eight herpesvirus types are known to infect humans (HHV-1 to HHV-8), and both primary infection and reactivation can lead to a wide spectrum of clinical manifestations, such as encephalitis, congenital defects and cancer. EBV is causally associated with various types of lymphoma and nasopharyngeal cancer, and has been classified as Group I human carcinogen by IARC.[21] This study shows that the age-standardized sero-prevalence of HHV-1 and CMV (HHV-5) is much higher in China (93.4% and 92.7%) than the UK (68.6% and 55.5%) or the US (48.1% and 50.4%), while HHV-2 is lower in China (8.3%) than the UK (16.8%) or the US (12.1%).[9] Similar ethnic differences were also observed within

For peer review only

For peer review only - http://bmjopen.bmj.com/site/about/guidelines.xhtml

the UKB population,[9] with Asians (77.9% and 92.0%; n=236) having a higher sero-prevalence of HHV-1 and CMV than white participants (69.1% and 56.5%; n=9140). Moreover, we also observed 4-fold (4.3~17.8%) geographical differences for HHV-2 across the 10 areas in this study. Limited studies conducted in China have demonstrated HHV-2 sero-prevalence as high as 13.2% among 2141 rural residents (5-60 years) of Zhejiang province in 2006,[22] and as low as 3.4% among a general population of 8074 residents (18-49 years) of Shandong province in 2016, with rates typically higher in women than men.[8] HHV-1, CMV and EBV are ubiquitous herpesviruses that usually are transmitted by contact with infected saliva or breastfeeding, whereas HHV-2 is almost always transmitted through sexual contacts.[23] Ethnic and geographical differences in sero-prevalences might be explained by differences in behaviour that facilitate transmission.[24] For example, a multi-ethnic population-based prospective cohort study of 4464 Dutch children reported that lifestyle factors including low family net household income, low maternal educational level and crowding could explain up to 48% of the ethnic differences in HHV-1 sero-prevalences, and up to 39% of the ethnic differences in EBV sero-prevalences.[24]

Human polyomaviruses

Polyomaviruses are potentially tumorigenic in humans. The observed sero-prevalences of BKV (91.1%), JCV (65.6%), and MCV (64.9%) in the present study was similar to those reported previously among Chinese using multiplex serology (206 controls with mean age of 58 years: BKV 91.3%, JCV 73.3% and MCV 67.0%; 5548 participants with mean age of 61 years: MCV 61.0%).[25 26] Limited data exist on the individual characteristics that relate to polyomaviruses sero-positivity in Chinese

For peer review only - http://bmjopen.bmj.com/site/about/guidelines.xhtml

populations. An increasing sero-prevalence of JCV and MCV with age was found among Chinese or US populations, and may be explained by a combination of continuous transmission of the pathogen throughout life and persistent antibody responses.

H. pylori

Previous studies have reported a substantial regional variation in *H. pylori* prevalence globally with China having a relatively high prevalence. In a meta-analysis of 670,572 participants from 98 Chinese studies during 1983-2018, using different methods to measure *H. pylori*, 49.6% overall (ranging from 20.6% to 81.8%) were sero-positive for *H. pylori.*[27] The present study also observed a significantly higher age-standardized sero-prevalence in China than UK (50.5% vs. 34.1%) using multiplex serology (**Supplementary Table 3**). Within China, regional differences in *H. pylori* sero-prevalence have not been previously explored and we found substantial variation across the 10 study areas, which may be attributed partly to levels of socio-economic development and sanitation, as *H. pylori* is commonly transmitted person-to-person by saliva, bouts of gastroenteritis within households and/or spread by fecal contamination of food or water.[28 29] There is a good evidence that a combination of untreated water, crowded conditions, and poor hygiene contributes to higher *H. pylori* prevalence in developing countries.[27 30] However, we were not able to explore these factors comprehensively due to the limited information available, but, as in many other populations, we observed a declining or plateaued sero-prevalence of *H. pylori* by birth cohort.[31] Sero-positivity for *H. pylori* was less common in participants with a history of peptic ulcer, which may reflect reverse causation as individuals with peptic

For peer review only - http://bmjopen.bmj.com/site/about/guidelines.xhtml

ulcer would be treated by antibiotics to eradicate *H. pylori*, leading to lower sero-prevalence.

C. trachomatis

C. trachomatis is the most commonly diagnosed bacterial sexually transmitted infection (STI) worldwide. Since *C. trachomatis* has not yet been included as a reportable STI in the national STI surveillance program in China, detailed epidemiological data is lacking. Previous population-based studies reported a *C. trachomatis* current infection prevalence of 2.1-4.1% in Chinese,[6] while in the present study which measured current and past infection status prevalence was 47.6% overall, which was higher in older participants likely due to cumulative exposure of the infection. Although both HHV-2 and *C. trachomatis* are sexually transmitted, there was a 5-fold difference in sero-prevalence between these two pathogens in CKB (8.3% vs 44.1%), which was not evident in the UKB population (16.8% vs 23.1%). This is unexplained and warrants further investigation. Individuals with higher socioeconomic status were less likely to be infected, which was also reported in a national stratified probability sample of 3426 Chinese (aged 20 to 64 years, 49.3% women) in 1999-2000.[6]

T. gondii

T. gondii is a zoonotic parasite infecting all warm-blooded animals. Usually, infected humans are asymptomatic, but in pregnant women the infection may lead to abortion, stillbirth or other serious consequences in newborns. Based on two national serological surveys in 15 provinces in China, the ELISA-based sero-prevalence of *T. gondii* increased from 5.2% in 1988-1992 to 7.9% in 2001-2004.[32 33] A recent serological survey involving 2634 healthy individuals in China showed a 12.5% anti-

For peer review only - http://bmjopen.bmj.com/site/about/guidelines.xhtml

T. gondii IgG positive rate,[34] which is comparable to our estimate (14.2%). Consistent with previous findings in China, we found the sero-prevalence was highest in Liuzhou (18.4%), Guangxi province, where the consumption of raw or undercooked meat is popular among local residents, which is an important factor in the prevalence of food-borne parasitic diseases.[35]

Strengths & Limitations

The main strength of the present study is the simultaneous assessment of antibodies against multiple pathogens, using a validated and custom designed multiplex serology platform. Moreover, the coverage of ten different areas in China enabled investigation of geographic variations in the sero-prevalence of pathogens, in addition to associations with socioeconomic and lifestyle factors. Furthermore, the broad age range of participants also allowed assessment of differences in the pathogens' sero-prevalence across the different birth cohorts and/or age groups. Nevertheless, the study also has limitations. First, the multiplex serology measurements are designed to detect cumulative (i.e. past and present) exposure in a high-throughput setting and may be less sensitive or specific than assays used in clinical diagnosis. Second, the information collected about possible determinants or correlates for certain infections was limited, particularly in relation to sexual behaviours. Third, the number of participants tested did not allow us to explore prospectively the associations of the sero-prevalence of multiple pathogens with the incidence of cancers and other NCDs.

Conclusions

In summary, among Chinese adults, the burden of pathogen infections was high and varied by area, sex, birth cohort and certain socioeconomic and lifestyle factors.

For peer review only

While some infections may represent past or transient infections with little or no major health consequences, others may represent chronic and persistent infections, which may lead to increased risk of certain cancers and many other NCDs. The development of a custom designed multiplex panel and its application in CKB should provide a reliable basis for future well-powered and prospective investigations of the associations between chronic infections and consequent disease outcomes in the Chinese population.

For peer review only

For peer review only - http://bmjopen.bmj.com/site/about/guidelines.xhtml

Contributors

PY, IM, CK, LY, ZC had full access to the study data. RJ, JB and TW conducted the assay measurement in the DKFZ Laboratory. All authors were involved in study design, analysis of data, interpretation, and writing of the report, and had final responsibility for the decision to submit for publication.

Declaration of interests

We declare that we have no conflict of interest.

Data sharing

Anonymised baseline, resurvey, and cause-specific mortality and morbidity data are available for access through a formal application in CKB website (www.ckbiobank.org). The application will then be reviewed by a Data Access Committee. Further details about access policy and procedures can be found online at www.ckbiobank.org.

Acknowledgments

The present study was funded by CRUK PRC-Project Award (C56488/A24504). The China Kadoorie Biobank (CKB) baseline survey was supported by the Kadoorie Charitable Foundation, Hong Kong. CKB long-term follow-up was supported by grants from the UK Wellcome Trust (212946/Z/18/Z, 202922/Z/16/Z, 104085/Z/14/Z, 088158/Z/09/Z), National Natural Science Foundation of China (91843302), and National Key Research and Development Program of China (2016YFC 0900500, 0900501, 0900504, 1303904). The UK Medical Research Council (MC_UU_00017/1, MC_UU_12026/2 MC_U137686851), Cancer Research UK (C16077/A29186; C500/A16896) and British Heart Foundation (CH/1996001/9454), provide core funding for the project. The chief acknowledgment is to the participants, the project staff, and the China National Centre for Disease Control and Prevention (CDC) and its regional offices for access to death and disease registries. The Chinese National Health Insurance scheme provides electronic linkage to all hospitalised individuals.

For peer review only - http://bmjopen.bmj.com/site/about/guidelines.xhtml

References

1. de Martel C, Georges D, Bray F, et al. Global burden of cancer attributable to infections in 2018: a worldwide incidence analysis. *The Lancet Global Health* 2020;8(2):e180-e90. doi: 10.1016/S2214-109X(19)30488-7
2. Smieja M, Gnarpe J, Lonn E, et al. Multiple infections and subsequent cardiovascular events in the Heart Outcomes Prevention Evaluation (HOPE) study. *Circulation* 2003;107(2):251-57. doi: doi:10.1161/01.CIR.0000044940.65226.1F
3. Giovannoni G, Cutter GR, Lunemann J, et al. Infectious causes of multiple sclerosis. *Lancet Neurol* 2006;5(10):887-94. doi: 10.1016/s1474-4422(06)70577-4 [published Online First: 2006/09/22]
4. Readhead B, Haure-Mirande JV, Funk CC, et al. Multiscale analysis of independent Alzheimer's cohorts finds disruption of molecular, genetic, and clinical networks by Human Herpesvirus. *Neuron* 2018;99(1):64-82.e7. doi: 10.1016/j.neuron.2018.05.023 [published Online First: 2018/06/26]
5. Rubicz R, Leach CT, Kraig E, et al. Seroprevalence of 13 common pathogens in a rapidly growing U.S. minority population: Mexican Americans from San Antonio, TX. *BMC Res Notes* 2011;4:433-33. doi: 10.1186/1756-0500-4-433
6. Parish WL, Laumann EO, Cohen MS, et al. Population-based study of Chlamydial infection in China: a hidden epidemic. *JAMA* 2003;289(10):1265-73. doi: 10.1001/jama.289.10.1265
7. Ji J, Sun H-K, Smith JS, et al. Seroprevalence of Human Papillomavirus types 6, 11, 16 and 18 in Chinese women. *BMC Infect Dis* 2012;12(1):137. doi: 10.1186/1471-2334-12-137
8. Huai P, Li F, Li Z, et al. Seroprevalence and associated factors of HSV-2 infection among general population in Shandong Province, China. *BMC Infect Dis* 2019;19(1):382. doi: 10.1186/s12879-019-3995-2
9. Mentzer AJ, Brenner N, Allen N, et al. Identification of host-pathogen-disease relationships using a scalable Multiplex Serology platform in UK Biobank. *medRxiv* 2019:19004960. doi: 10.1101/19004960
10. Xia G-L, Liu C-B, Cao H-L, et al. Prevalence of hepatitis B and C virus infections in the general Chinese population. Results from a nationwide cross-sectional seroepidemiologic study of hepatitis A, B, C, D, and E virus infections in China, 1992. *International Hepatology Communications* 1996;5(1):62-73. doi: https://doi.org/10.1016/S0928-4346(96)82012-3
11. Liang X, Bi S, Yang W, et al. Epidemiological serosurvey of Hepatitis B in China—Declining HBV prevalence due to Hepatitis B vaccination. *Vaccine* 2009;27(47):6550-57. doi: https://doi.org/10.1016/j.vaccine.2009.08.048
12. Liu J, Zhang S, Wang Q, et al. Seroepidemiology of hepatitis B virus infection in 2 million men aged 21–49 years in rural China: a population-based, cross-sectional study. *The Lancet Infectious Diseases* 2016;16(1):80-86. doi: https://doi.org/10.1016/S1473-3099(15)00218-2
13. Chen Z, Chen J, Collins R, et al. China Kadoorie Biobank of 0.5 million people: survey methods, baseline characteristics and long-term follow-up. *Int J Epidemiol* 2011;40(6):1652-66. doi: 10.1093/ije/dyr120
14. Waterboer T, Sehr P, Michael KM, et al. Multiplex Human Papillomavirus serology based on in situ–purified glutathione S-transferase fusion proteins. *Clinical Chemistry* 2005;51(10):1845-53. doi: 10.1373/clinchem.2005.052381
15. Brenner N, Mentzer AJ, Butt J, et al. Validation of Multiplex Serology for human hepatitis viruses B and C, human T-lymphotropic virus 1 and Toxoplasma gondii. *PLoS One* 2019;14(1):e0210407. doi: 10.1371/journal.pone.0210407
16. Bassig BA, Willhauck-Fleckenstein M, Shu X-O, et al. Serologic markers of viral infection and risk of non-Hodgkin lymphoma: A pooled study of three prospective cohorts in China and Singapore. *Int J Cancer* 2018;143(3):570-79. doi: 10.1002/ijc.31385
17. Arbyn M, Weiderpass E, Bruni L, et al. Estimates of incidence and mortality of cervical cancer in 2018: a worldwide analysis. *The Lancet Global Health* 2020;8(2):e191-e203. doi: 10.1016/S2214-109X(19)30482-6

For peer review only - http://bmjopen.bmj.com/site/about/guidelines.xhtml

18. Chen C-J, Viscidi RP, Chuang C-H, et al. Seroprevalence of human papillomavirus types 16 and 18 in the general population in Taiwan: Implication for optimal age of human papillomavirus vaccination. *J Clin Virol* 2007;38(2):126-30. doi: https://doi.org/10.1016/j.jcv.2006.12.004

19. Liu F, Deng Q, Zhang C, et al. Human papillomavirus DNA positivity and seropositivity in rural Chinese men and women: a population-based cross-sectional study. *Sci Rep* 2016;6(1):26343. doi: 10.1038/srep26343

20. Ma X, Wang Q, Ong JJ, et al. Prevalence of human papillomavirus by geographical regions, sexual orientation and HIV status in China: a systematic review and meta-analysis. *Sexually Transmitted Infections* 2018;94(6):434-42. doi: 10.1136/sextrans-2017-053412

21. Niedobitek G. The Epstein-Barr virus: a group 1 carcinogen? *Virchows Arch* 1999;435(2):79-86. doi: 10.1007/s004280050402 [published Online First: 1999/12/22]

22. Lin H, He N, Su M, et al. Herpes simplex virus infections among rural residents in eastern China. *BMC Infect Dis* 2011;11(1):69. doi: 10.1186/1471-2334-11-69

23. Grinde B. Herpesviruses: latency and reactivation - viral strategies and host response. *J Oral Microbiol* 2013;5:10.3402/jom.v5i0.22766. doi: 10.3402/jom.v5i0.22766

24. Jansen MAE, van den Heuvel D, Bouthoorn SH, et al. Determinants of ethnic differences in Cytomegalovirus, Epstein-Barr Virus, and Herpes Simplex Virus type 1 seroprevalence in childhood. *The Journal of Pediatrics* 2016;170:126-34.e6. doi: https://doi.org/10.1016/j.jpeds.2015.11.014

25. Malhotra J, Waterboer T, Pawlita M, et al. Serum biomarkers of polyomavirus infection and risk of lung cancer in never smokers. *Br J Cancer* 2016;115(9):1131-39. doi: 10.1038/bjc.2016.285

26. Zhang C, Liu F, He Z, et al. Seroprevalence of Merkel Cell Polyomavirus in the general rural population of Anyang, China. *PLoS One* 2014;9(9):e106430. doi: 10.1371/journal.pone.0106430

27. Li M, Sun Y, Yang J, et al. Time trends and other sources of variation in Helicobacter pylori infection in mainland China: A systematic review and meta-analysis. *Helicobacter* 2020;25(5):e12729. doi: https://doi.org/10.1111/hel.12729

28. Klein PD, Opekun AR, Smith EO, et al. Water source as risk factor for Helicobacter pylori infection in Peruvian children. *The Lancet* 1991;337(8756):1503-06. doi: https://doi.org/10.1016/0140-6736(91)93196-G

29. Perry S, de la Luz Sanchez M, Yang S, et al. Gastroenteritis and transmission of Helicobacter pylori infection in households. *Emerg Infect Dis* 2006;12(11):1701-8. doi: 10.3201/eid1211.060086 [published Online First: 2007/02/08]

30. Hooi JKY, Lai WY, Ng WK, et al. Global prevalence of Helicobacter pylori infection: systematic review and meta-analysis. *Gastroenterology* 2017;153(2):420-29. doi: 10.1053/j.gastro.2017.04.022 [published Online First: 2017/05/01]

31. Nagy P, Johansson S, Molloy-Bland M. Systematic review of time trends in the prevalence of Helicobacter pylori infection in China and the USA. *Gut Pathog* 2016;8:8-8. doi: 10.1186/s13099-016-0091-7

32. Yu S, Xu L, Jiang Z, et al. Report on the first nationwide survey of the distribution of human parasites in China, regional distribution of parasite species. *Chinese Journal of Parasitology and Parasitic Diseases* 1994;12(4):241-47.

33. Zhou P, Chen N, Zhang R-L, et al. Food-borne parasitic zoonoses in China: perspective for control. *Trends Parasitol* 2008;24(4):190-96.

34. Xiao Y, Yin J, Jiang N, et al. Seroepidemiology of human Toxoplasma gondii infection in China. *BMC Infect Dis* 2010;10(1):4.

35. Diseases COotNSotIHP. A national survey on current status of the important parasitic diseases in human population. *Chinese Journal of Parasitology and Parasitic Diseases* 2005;23(5 Suppl):332.

For peer review only

Figure legends:

Figure 1. Sero-prevalence of multiple pathogens by year of birth

Dots and grey lines represent sero-prevalence estimates and 95% CI for every single birth year. The blue line represents estimates for these sero-prevalences derived from LOESS method, and the shaded areas show the 95% CI. Due to few participants that were born before 1932 or after 1971, those who were born between 1927-1931, or 1971-1976 were combined, separately.

Figure 2. Sero-prevalence of multiple pathogens across 10 study areas

The grey bar represents urban participants and white bar represents rural participants.

Figure 3. Distribution of number of infections for (a, b) any of 19 pathogens; and (c, d) oncogenic pathogens, overall and by year of birth

Numbers with different infections ([a] and [c]), and sero-prevalence for >10 pathogens (b), or >2 pathogens (d) by birth years were presented. Pathogens of HBV, HCV, HIV, HTLV, *H. pylori*, HPV (16, 18) and EBV are classified as Group I human carcinogen by the IARC. The dashed lines represent the mean numbers of seropositive pathogens among the study participants, which were 9.4 (SD 1.7) for any of 19 pathogens, and 2.1 (SD 0.8) for any of 8 oncogenic pathogens. Proportion of individuals with >10 infectious pathogens, or with >2 oncogenic pathogens was presented by birth cohort. Due to few participants that were born before 1932 or after 1971, those who were born between 1927-1931, or 1971-1976 were combined, separately. The blue line represents estimate for these sero-prevalence derived from LOESS method, and the shaded areas show the 95% CI.

For peer review only

Table 1. Baseline characteristics of the study subcohort and overall CKB cohort

Characteristics	Subcohort (n=1,986)	CKB (n=453,157)
**Demographic and lifestyle factors**		
Age, years	51.8 (10.8)	51.7 (10.6)
Birth year, %		
≤1939	12.0	11.0
1940-1949	20.0	20.6
1950-1959	33.2	31.9
1960-1969	29.2	30.8
≥1970	5.6	5.7
Female, %	61.7	60.2
Urban residents, %	50.3	45.4
Education ≥high school, %	21.9	21.5
Household income (>20000 yuan/year), %	42.4	43.2
Living alone, %	3.6	2.8
Ever regular smoker in men, %	73.9	74.0
Ever regular smoker in women, %	3.6	3.1
Ever regular drinker in men, %	38.4	36.9
Ever regular drinker in women, %	3.2	2.4
Physical activity, MET h/day	21.1 (14.2)	21.3 (13.9)
**Medical history and health status, %**		
Poor self-rated health	10.2	10.0
HBsAg positive [1]	3.2	3.1
Diabetes [2]	5.7	5.6
History of peptic ulcer	3.9	3.9
History of cirrhosis/chronic hepatitis	1.3	1.2
Family history of cancer	14.8	14.2
**Anthropometry, mean (SD)**		
BMI, kg/m^2	23.8 (3.5)	23.6 (3.4)
Waist circumference, cm	80.1 (9.9)	80.1 (9.7)
SBP, mmHg	130 (21)	130 (21)
Random plasma glucose, mmol/L	5.8 (2.3)	6.0 (2.3)

The results are presented as means (SD) or percentages. Abbreviations: MET-h, metabolic equivalent of task-hours; SBP, systolic blood pressure; HBsAg, Hepatitis B surface antigen;

[1] Hepatitis B surface antigen (HBsAg) was tested using an on-site rapid test strip at baseline

[2] Includes those with a (self-reported) previous medical diagnosis of diabetes and those detected by blood glucose tests at baseline

For peer review only

For peer review only - http://bmjopen.bmj.com/site/about/guidelines.xhtml

Table 2. Sero-prevalence estimates (95% CI) for infectious pathogens in men and women

Pathogens	Men (n=761)	Women (n=1225)	All (n=1986)
**Hepatitis viruses**			
HBV	46.1 (42.5-49.7)	42.4 (39.6-45.2)	43.8 (41.6-46.0)
HCV	0.5 (0.1-1.3)	0.8 (0.4-1.5)	0.7 (0.4-1.2)
**Human papillomaviruses (HPV)**			
HPV-16	4.1 (2.8-5.7)	7.9 (6.5-9.6)	6.4 (5.4-7.6)
HPV-18	2.8 (1.7-4.2)	3.8 (2.8-5.0)	3.4 (2.6-4.3)
**Human herpesviruses (HHV)**			
HHV-1	97.2 (95.8-98.3)	98.4 (97.6-99.1)	98.0 (97.3-98.6)
HHV-2	7.1 (5.4-9.2)	10.0 (8.3-11.8)	8.9 (7.6-10.2)
VZV	92.9 (90.8-94.6)	90.7 (88.9-92.3)	91.5 (90.2-92.7)
EBV	99.9 (99.3-100.0)	99.8 (99.4-100.0)	99.8 (99.6-100.0)
CMV	95.9 (94.3-97.2)	98.1 (97.2-98.8)	97.3 (96.5-98.0)
HHV-6	52.3 (48.7-55.9)	68.0 (65.3-70.6)	62.0 (59.8-64.1)
HHV-7	79.5 (76.5-82.3)	89.3 (87.4-91.0)	85.5 (83.9-87.1)
**Human polyomaviruses (HPyV)**			
BKV	90.8 (88.5-92.8)	91.3 (89.6-92.9)	91.1 (89.8-92.4)
JCV	68.5 (65.0-71.8)	63.8 (61.1-66.5)	65.6 (63.5-67.7)
MCV	64.7 (61.1-68.1)	65.1 (62.3-67.7)	64.9 (62.8-67.0)
**Other viruses**			
HIV	0	0.1 (0.0-0.5)	0.1 (0.0-0.3)
HTLV	0.1 (0.0-0.7)	0.1 (0.0-0.5)	0.1 (0.0-0.4)
**Bacteria/Parasite**			
H. pylori	54.3 (50.7-57.9)	53.0 (50.1-55.8)	53.5 (51.3-55.7)
C. trachomatis	45.7 (42.1-49.3)	48.8 (46.0-51.7)	47.6 (45.4-49.9)
T. gondii	12.4 (10.1-14.9)	15.3 (13.4-17.5)	14.2 (12.7-15.8)

For peer review only

Figure 1. Sero-prevalence of multiple pathogens by year of birth

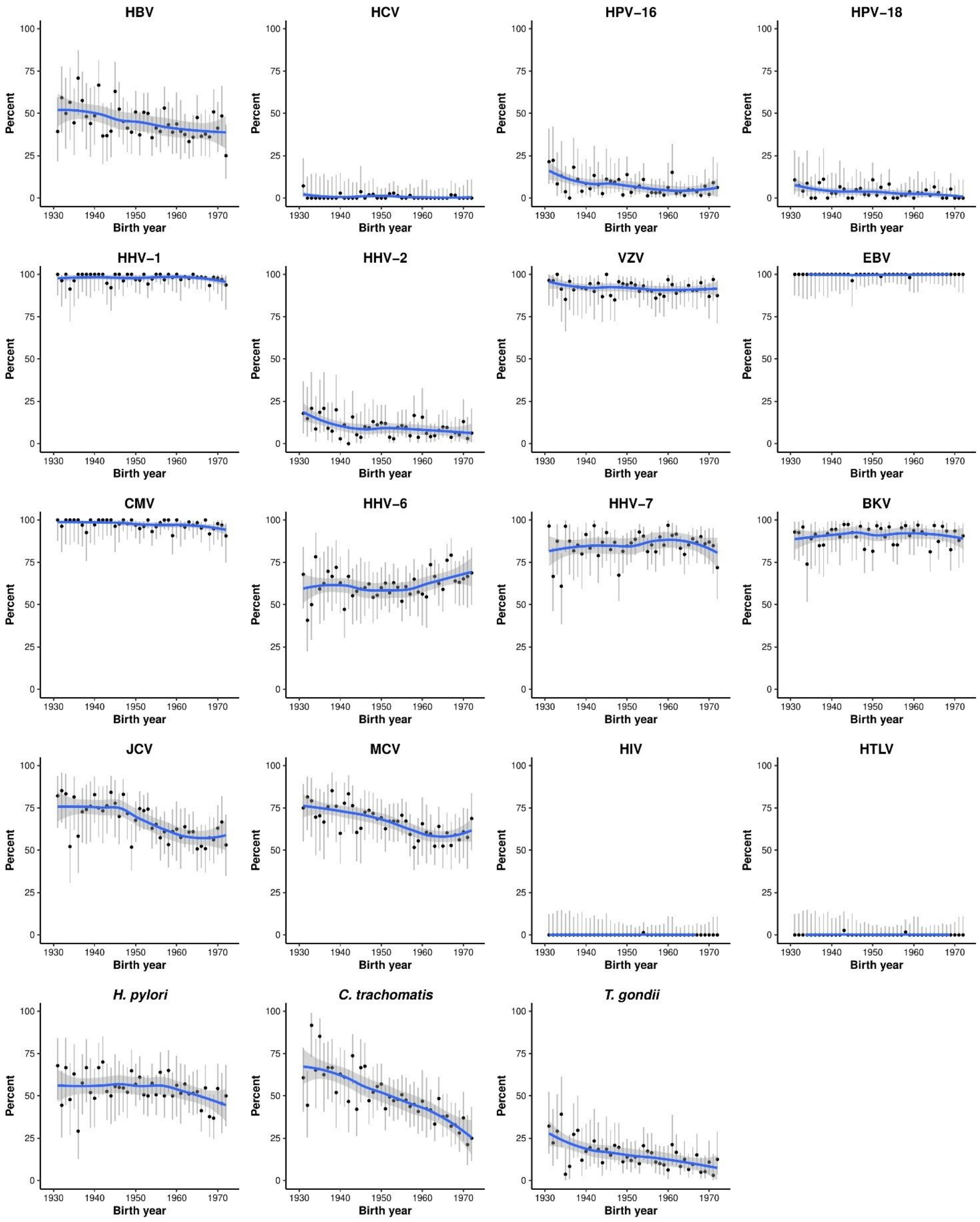

For peer review only - http://bmjopen.bmj.com/site/about/guidelines.xhtml

Figure 2. Sero-prevalence of multiple pathogens across 10 study areas

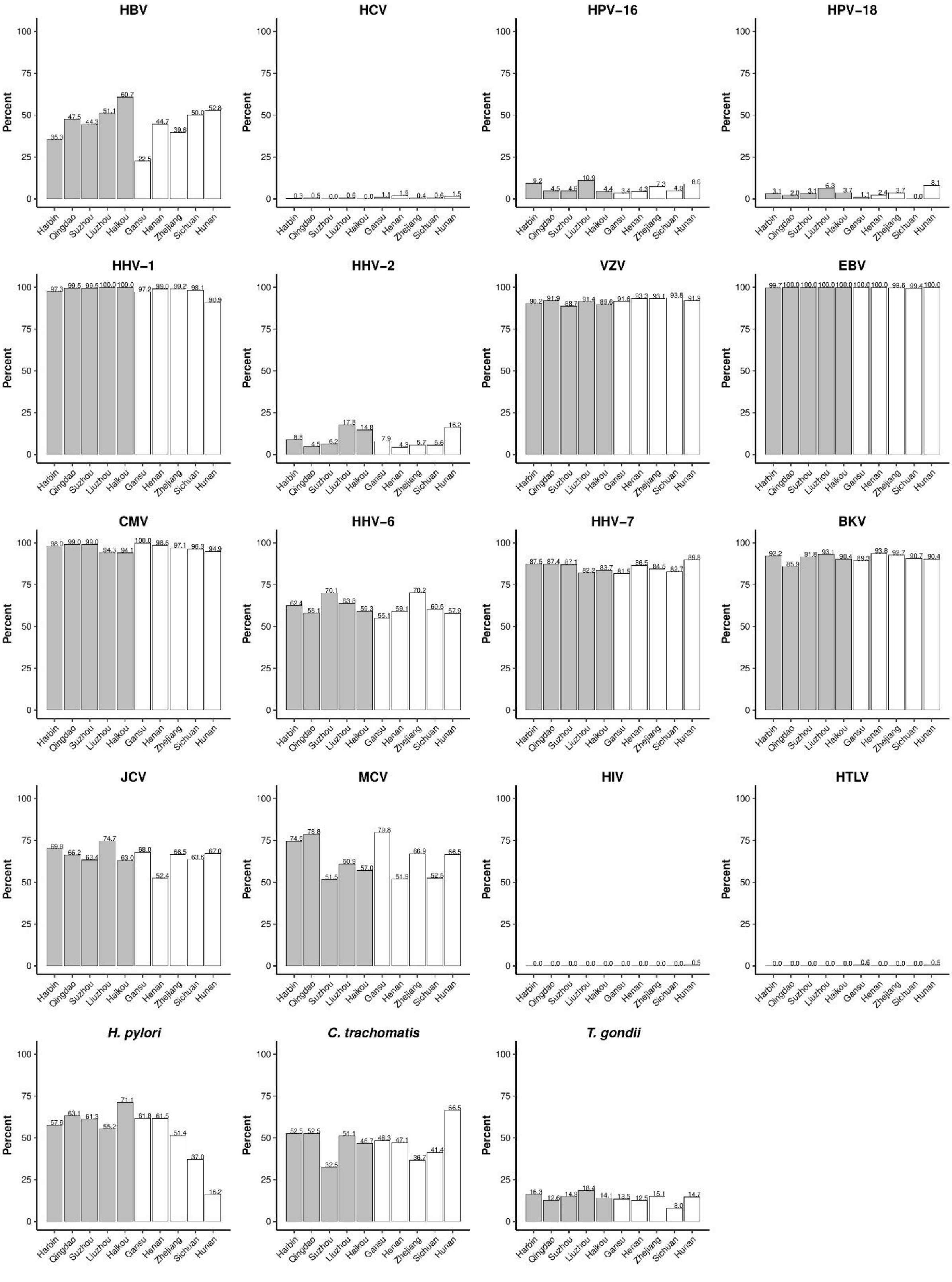

Figure 3. Distribution of number of infections for (a) any of 19 pathogens; and (b) oncogenic pathogens, overall and by year of birth

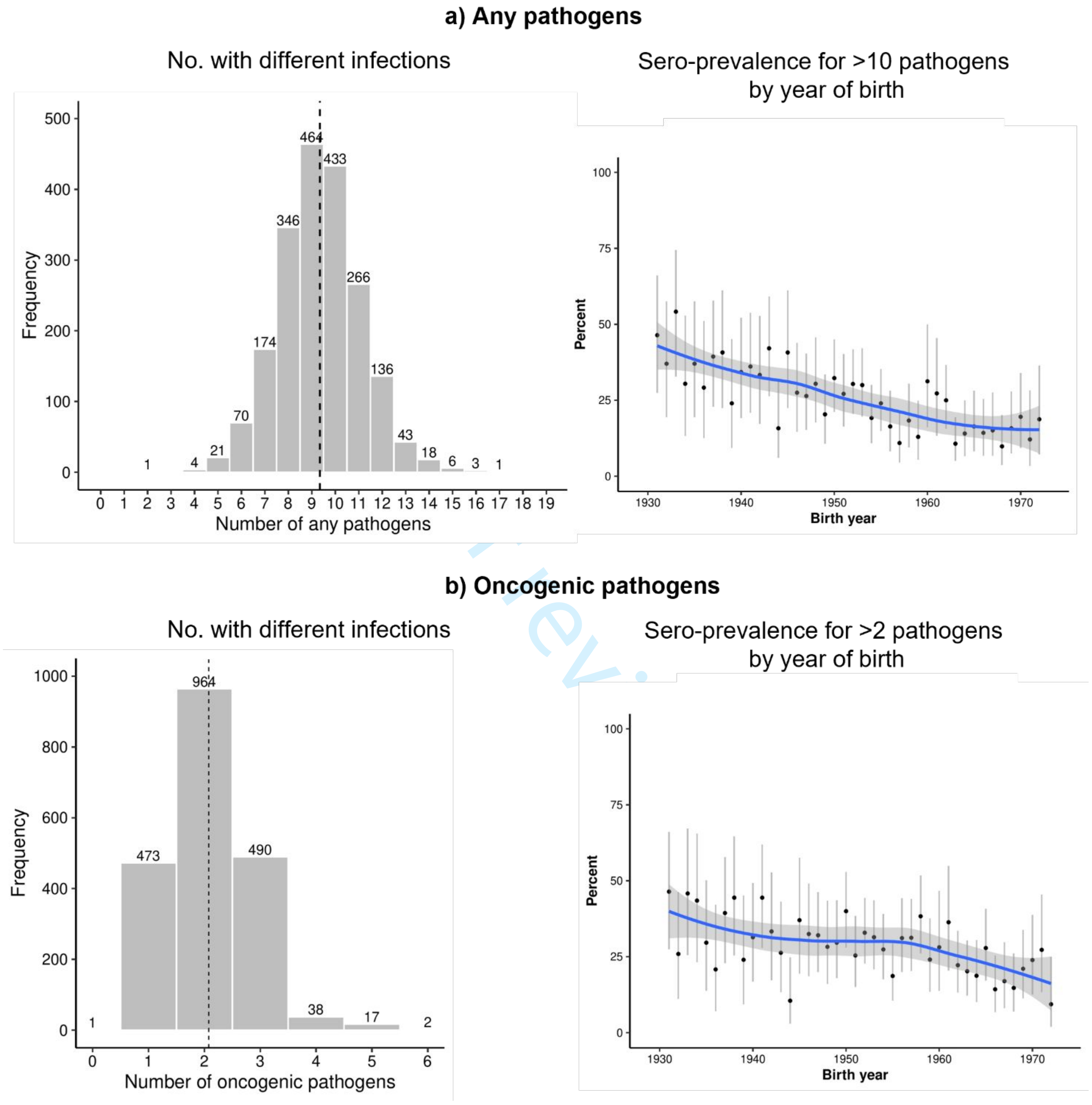

Sero-prevalence of 19 infectious pathogens and associated factors among adults from 10 urban and rural areas of China

Supplementary Material

For peer review only

For peer review only - http://bmjopen.bmj.com/site/about/guidelines.xhtml

eAppendix

Study population

Details of the CKB study design, methods, and participants have been previously reported. The 512,715 participants aged 30-79 years were enrolled from 10 (5 urban, 5 rural) geographically diverse regions in China (**Supplementary Figure 1**). At baseline (June 2004-July 2008) and at two subsequent resurveys in a 5% subset (in 2008 and 2013, respectively), detailed data were collected on socio-demographic characteristics, smoking, alcohol consumption, diet, physical activity, personal and family medical history, and physical measurements. Follow-up of CKB participants was through linkage to established mortality and morbidity registries, and to the nationwide health insurance system which records all hospitalised episodes. Ethics approval was obtained from the Oxford University, the China National CDC, and also from institutional research boards at the local CDCs in the 10 areas. All participants provided written informed consent.

Repeat measurements

For 100 (5%) of these participants, plasma samples collected at the two subsequent resurveys were also assayed to assess the consistency of sero-status (sero-conversion and sero-reversion rates) of measurements over time.

The agreement between multiple measurements of same individuals at three occasions (baseline, the 1st and the 2nd resurvey) of quantitative MFI antibody levels was evaluated using Intraclass Correlation Coefficients (ICCs).

The MFI values were moderately to highly correlate between the baseline survey, and the 1st and 2nd resurveys (**Supplementary Figure 5-7**). For example, the mean (SD)

For peer review only - http://bmjopen.bmj.com/site/about/guidelines.xhtml

ICC for 43 antigens was 0.76 (0.18) between baseline and the 1st resurvey (mean SD duration of 2.5 [0.7] years). The apparent sero-conversion and sero-reversion rates were generally low (ranging from 0%-11.3%) across surveys (**Supplementary Table 3**).

For peer review only

For peer review only - http://bmjopen.bmj.com/site/about/guidelines.xhtml

Table S1. Characteristics of pathogens and pathogen-specific antigens included in the multiplex serology and suggested cut-offs to determine seropositivity

Pathogen	Transmissions [1]	Antigen	Full name, function	Criteria for seropositivity	Cut-off (MFI)*
**Hepatitis viruses**					
Hepatitis B virus (HBV)	Perinatal, blood, semen	HBVAc	core antigen	2 positives out of 2	1000
		HBVAe	soluble nucleocapsid associated antigen		1000
Hepatitis C virus (HCV)	blood, semen	1aCore	structual antigen	2 positives out of 2	**300**
		1aNS3	protease and RNA helicase activity		150
**Human papillomaviruses (HPV)**					
HPV-16	sexual contact	L1	major capsid protein		80
		E6	oncoproteins		**100**
		E7	oncoproteins		50
HPV-18	sexual contact	L1	major capsid protein		170
**Human herpesviruses (HHV)**					
HHV-1 (HSV-1/)	sores, saliva, and surface in or around mouth	gG	membrane glycoprotein		54
HHV-2 (HSV-2)	genital surfaces, skin, sores or fluids	mgG unique	membrane glycoprotein		180
Varicella zoster virus (VZV/HHV-3)	blisters, saliva or mucus	gE/gI	envelope glycoproteins		100
Epstein-Barr virus (EBV/HHV-4)	saliva, blood or semen	ZEBRA	replication activator	≥2 positives out of 4	74
		EA-D	replication (polymerase accessory subunit)		110
		VCAp18	capsid protein		2526
		EBNA (peptid)	replication, latent viral infection		411
Human cytomegalovirus (CMV/HHV-5)	saliva, urine, blood, tears, semen or breast milk	pp150 Nter	tegument protein	≥2 positives out of 3	655
		pp52	DNA binding protein		1101
		pp28	capsid protein		200
HHV-6	saliva	IE1A	potential transactivator	≥1 positive out of 2	300
		IE1B	potential transactivator		300
HHV-7	saliva	U14	potential tegument protein		**100**
**Human polyomaviruses (HPyV)**					
HPyV BK (BKV)	respiratory fluids or urine	VP1	major capsid protein		250
HPyV JC (JCV)	respiratory fluids or urine	VP1	major capsid protein		250
HPyV MCV (MCV)	respiratory fluids	VP1	major capsid protein		250
**Other viruses**					
Human immunodeficiency virus (HIV)	blood, semen, vaginal and rectal secretions, or breast milk	gag	structural antigen	2 positives out of 2	1000
		env	structural antigen		150
Human T-lymphotropic virus (HTLV)	breast milk, blood,	gag	structural antigen	2 positives out of 2	700
		nv	structural antigen		50
**Bacteria/Parasite**					
Helicobacter pylori (H. pylori)	saliva (oral-oral, gastro-oral and fecal-oral)	HyuA	hydantoin utilization protein A	≥4 positives out of 12	**280**
		GroEL	chaperonin GroEL		500
		UreA	urease alpha subunit		**300**
		CagA-N/C	cytotoxin-associated antigen A, pathogenesis		1500
		Catalase			**300**
		HP1564	hypothetical protein		150
		VacA-N/C	vacuolating cytotoxin, pathogenesis		385
		NapA	neutrophil-activating protein		120
		HP0305	hypothetical protein		**130**
		HpaA	neuraminyllactose-binding hemagglutinin homolog		200
		Cad	cinnamyl alcohol dehydrogenase		100
		HcpC	conserved hypothetical secreted protein		150
Chlamydia trachomatis (C. trachomatis)	sexual contact	pGP3	virulence factor		**300**
Toxoplasma gondii (T. gondii)	undercooked meat, blood	sag1	surface protein	≥1 positive out of 2	50
		p22	surface protein		50

MFI: Median Fluorescence Intensities

[1] The route of transmission of some pathogens is not completely understood, and the major route is listed

For peer review only

Table S2. Adjusted odds ratios of sero-positivity for selected pathogens by baseline characteristics

	HBV	HPV-16	HHV-2	VZV	HHV-6	HHV-7	BKV	JCV	MCV	*H. pylori*	*C. trachomatis*	*T. gondii*
Sex, female vs male	0.86 (0.72, 1.03)	**2.07 (1.36, 3.14)**	**1.45 (1.04, 2.03)**	0.76 (0.54, 1.06)	**1.92 (1.59, 2.31)**	**2.15 (1.67, 2.76)**	1.08 (0.78, 1.48)	0.82 (0.68, 1.00)	1.04 (0.85, 1.25)	0.93 (0.78, 1.12)	1.18 (0.98, 1.42)	1.31 (1.00, 1.71)
Age (reference group: ≤39 years)												
40-49	1.01 (0.77, 1.34)	0.73 (0.38, 1.41)	1.12 (0.66, 1.90)	0.84 (0.52, 1.37)	0.78 (0.58, 1.05)	0.99 (0.66, 1.48)	1.25 (0.78, 2.01)	1.03 (0.78, 1.36)	1.02 (0.77, 1.35)	1.21 (0.91, 1.59)	**1.43 (1.08, 1.91)**	1.33 (0.84, 2.11)
50-59	1.15 (0.87, 1.51)	1.51 (0.84, 2.73)	1.34 (0.80, 2.24)	1.08 (0.65, 1.78)	0.63 (0.47, 0.85)	0.92 (0.62, 1.37)	1.08 (0.68, 1.72)	**1.53 (1.15, 2.03)**	**1.54 (1.16, 2.04)**	**1.37 (1.04, 1.81)**	**2.18 (1.64, 2.90)**	**1.85 (1.18, 2.90)**
≥60	**1.50 (1.12, 2.00)**	**2.06 (1.14, 3.73)**	1.67 (0.99, 2.81)	1.09 (0.64, 1.85)	0.70 (0.52, 0.96)	0.94 (0.62, 1.42)	1.17 (0.71, 1.91)	**2.30 (1.69, 3.13)**	**2.01 (1.48, 2.72)**	1.29 (0.97, 1.73)	**3.51 (2.59, 4.74)**	**2.54 (1.62, 3.99)**
Region, urban vs rural	1.15 (0.96, 1.37)	1.12 (0.78, 1.62)	1.22 (0.89, 1.67)	0.74 (0.53, 1.02)	1.07 (0.89, 1.29)	1.04 (0.81, 1.34)	0.90 (0.66, 1.23)	1.17 (0.97, 1.41)	1.07 (0.89, 1.29)	**1.80 (1.50, 2.15)**	0.92 (0.77, 1.10)	1.14 (0.88, 1.47)
Education, ≥high school vs <high school	0.93 (0.72, 1.19)	**1.63 (1.01, 2.65)**	1.23 (0.81, 1.88)	0.87 (0.57, 1.32)	0.83 (0.64, 1.07)	1.09 (0.77, 1.54)	0.88 (0.58, 1.35)	0.9 (0.70, 1.17)	0.95 (0.73, 1.23)	1.09 (0.85, 1.41)	**0.70 (0.54, 0.90)**	1.16 (0.82, 1.65)
Household income, ≥20k vs <20k	1.03 (0.83, 1.27)	0.84 (0.56, 1.27)	0.89 (0.62, 1.28)	1.26 (0.87, 1.82)	0.89 (0.72, 1.11)	1.06 (0.78, 1.43)	0.95 (0.66, 1.37)	1.03 (0.82, 1.29)	0.98 (0.78, 1.23)	0.89 (0.71, 1.11)	**0.80 (0.64, 0.99)**	1.21 (0.90, 1.62)
Physical activity (high vs low)	1.27 (1.03, 1.56)	**0.50 (0.32, 0.78)**	0.82 (0.57, 1.19)	**0.65 (0.45, 0.94)**	0.91 (0.73, 1.12)	1.44 (1.08, 1.93)	0.88 (0.62, 1.26)	1.06 (0.86, 1.32)	0.99 (0.80, 1.23)	0.98 (0.79, 1.21)	0.88 (0.72, 1.09)	0.75 (0.56, 1.01)
Ever regular smoker (yes vs no)	1.15 (0.86, 1.55)	0.59 (0.31, 1.13)	0.88 (0.51, 1.51)	1.09 (0.63, 1.89)	1.00 (0.74, 1.34)	1.15 (0.79, 1.68)	0.93 (0.56, 1.55)	1.06 (0.77, 1.46)	**0.71 (0.51, 0.97)**	1.03 (0.76, 1.40)	0.92 (0.68, 1.25)	0.77 (0.50, 1.18)
Ever regular drinker (yes vs no)	1.08 (0.81, 1.42)	0.55 (0.27, 1.14)	0.84 (0.48, 1.47)	1.29 (0.75, 2.22)	1.01 (0.76, 1.33)	1.03 (0.72, 1.48)	1.10 (0.68, 1.78)	0.96 (0.72, 1.29)	1.06 (0.79, 1.43)	0.91 (0.69, 1.21)	1.01 (0.76, 1.34)	0.86 (0.56, 1.31)
Self-rated poor health (yes vs no)	0.90 (0.66, 1.23)	1.31 (0.75, 2.27)	1.11 (0.67, 1.82)	1.05 (0.61, 1.82)	0.88 (0.65, 1.19)	**0.60 (0.41, 0.88)**	0.97 (0.57, 1.63)	0.96 (0.69, 1.31)	0.96 (0.70, 1.32)	0.83 (0.61, 1.13)	0.98 (0.72, 1.34)	1.27 (0.85, 1.89)
Hepatitis B test (positive vs negative)	**11.51 (5.16, 25.7)**	0.57 (0.13, 2.40)	0.73 (0.26, 2.09)	0.90 (0.38, 2.13)	0.78 (0.46, 1.31)	1.97 (0.78, 5.02)	1.10 (0.43, 2.80)	1.11 (0.65, 1.90)	1.44 (0.83, 2.50)	1.14 (0.66, 1.97)	1.34 (0.79, 2.27)	0.77 (0.32, 1.82)
Diabetes (yes vs no)	1.20 (0.81, 1.77)	**2.69 (1.52, 4.77)**	1.31 (0.70, 2.45)	0.86 (0.44, 1.67)	0.86 (0.58, 1.28)	0.75 (0.45, 1.26)	1.25 (0.61, 2.55)	**0.52 (0.35, 0.78)**	1.22 (0.78, 1.91)	1.06 (0.71, 1.59)	**1.55 (1.03, 2.34)**	1.28 (0.78, 2.09)
History of peptic ulcer (yes vs no)	1.09 (0.68, 1.74)	0.19 (0.03, 1.38)	0.54 (0.19, 1.51)	1.23 (0.49, 3.11)	0.82 (0.51, 1.32)	1.87 (0.88, 3.98)	0.91 (0.41, 2.04)	0.89 (0.54, 1.47)	0.78 (0.48, 1.27)	**0.52 (0.32, 0.85)**	1.09 (0.67, 1.76)	0.80 (0.39, 1.64)
History of cirrhosis/chronic hepatitis (yes vs no)	**7.51 (2.55, 22.13)**	2.20 (0.62, 7.73)	0.46 (0.06, 3.49)	0.66 (0.19, 2.24)	0.86 (0.39, 1.92)	1.48 (0.44, 5.03)	2.21 (0.30, 16.47)	1.56 (0.64, 3.80)	0.88 (0.39, 1.97)	0.79 (0.35, 1.75)	0.57 (0.24, 1.37)	0.55 (0.13, 2.35)
Family history of cancer	1.13 (0.87, 1.47)	1.05 (0.62, 1.80)	0.92 (0.56, 1.51)	0.86 (0.56, 1.33)	0.94 (0.72, 1.23)	0.85 (0.59, 1.21)	1.38 (0.84, 2.26)	1.19 (0.91, 1.57)	0.99 (0.76, 1.31)	1.18 (0.91, 1.54)	0.82 (0.63, 1.07)	0.96 (0.66, 1.39)
BMI≥25 kg/m² (yes vs no)	1.09 (0.89, 1.32)	**1.51 (1.03, 2.22)**	1.05 (0.75, 1.49)	1.03 (0.73, 1.46)	0.93 (0.76, 1.14)	0.80 (0.61, 1.05)	1.06 (0.75, 1.48)	1.00 (0.82, 1.23)	1.12 (0.91, 1.37)	0.93 (0.76, 1.14)	1.01 (0.82, 1.23)	1.02 (0.77, 1.34)
Central obesity (yes vs no)1	1.03 (0.83, 1.27)	1.29 (0.86, 1.93)	1.00 (0.70, 1.45)	1.01 (0.70, 1.45)	0.98 (0.79, 1.22)	0.93 (0.68, 1.27)	1.02 (0.71, 1.47)	**0.78 (0.62, 0.97)**	1.24 (0.99, 1.56)	0.98 (0.79, 1.22)	0.94 (0.75, 1.16)	0.95 (0.71, 1.28)

Adjust for age, sex and region (where appropriate). Bold values denote statistical significance at the $p < 0.05$ level.
1 Central obesity is defined as a waist circumference greater or equal to 94 cm in men and 80 cm in women.

For peer review only

For peer review only - http://bmjopen.bmj.com/site/about/guidelines.xhtml

Table S3. Sero-conversion and sero-reversion rates estimated from 100 individuals with samples available at baseline, 1st resurvey and 2nd resurvey

Pathogen	Baseline - 1st resurvey		Baseline - 2nd resurvey		1st resurvey - 2nd resurvey	
	Sero-conversion (%)	Sero-reversion (%)	Sero-conversion (%)	Sero-reversion (%)	Sero-conversion (%)	Sero-reversion (%)
**Hepatitis viruses**						
HBV	2 (2.1)	2 (2.1)	2 (2.0)	2 (2.0)	4 (4.1)	4 (4.1)
HCV	0 (0)	1 (1.0)	0 (0)	1 (1.0)	0 (0)	0 (0)
**Human papillomaviruses (HPV)**						
HPV-16	1 (1.0)	4 (4.1)	1 (1.0)	4 (4.1)	3 (3.1)	3 (3.1)
HPV-18	0 (0)	2 (2.1)	1 (1.0)	1 (1.0)	1 (1.0)	1 (1.0)
**Human herpesviruses (HHV)**						
HHV-1	0 (0)	0 (0)	0 (0)	0 (0)	0 (0)	0 (0)
HHV-2	3 (3.1)	0 (0)	4 (4.1)	0 (0)	1 (1.0)	0 (0)
VZV	5 (5.2)	4 (4.1)	3 (3.1)	2 (2.0)	3 (3.1)	3 (3.1)
EBV	0 (0)	0 (0)	0 (0)	0 (0)	0 (0)	0 (0)
CMV	1 (1.0)	0 (0)	1 (1.0)	0 (0)	0 (0)	0 (0)
HHV-6	11 (11.3)	11 (11.3)	8 (8.2)	12 (12.2)	8 (8.2)	11 (11.2)
HHV-7	7 (7.2)	10 (10.3)	8 (8.2)	5 (5.1)	11 (11.2)	4 (4.1)
**Human polyomaviruses (HPyV)**						
BKV	2 (2.1)	7 (7.2)	4 (4.1)	4 (4.1)	8 (8.2)	2 (2.0)
JCV	2 (2.1)	3 (3.1)	2 (2.0)	3 (3.1)	3 (3.1)	3 (3.1)
MCV	2 (2.1)	5 (5.2)	3 (3.1)	6 (6.1)	2 (2.0)	3 (3.1)
**Other viruses**						
HIV	0 (0)	0 (0)	0 (0)	0 (0)	0 (0)	0 (0)
HTLV	0 (0)	0 (0)	0 (0)	0 (0)	0 (0)	0 (0)
**Bacteria/Parasite**						
H. pylori	2 (2.1)	4 (4.1)	3 (3.1)	12 (12.2)	3 (3.1)	10 (10.2)
C. trachomatis	3 (3.1)	2 (2.1)	4 (4.1)	1 (1.0)	2 (2.0)	0 (0)
T. gondii	4 (4.1)	8 (8.2)	3 (3.1)	6 (6.1)	7 (7.1)	6 (6.1)

Sero-conversion referred to the process of having "become pathogen positive", and sero-reversion is the opposite of seroconversion

For peer review only - http://bmjopen.bmj.com/site/about/guidelines.xhtml

For peer review only

Table S4. Age-standardized sero-prevalence (%) for infectious pathogens in CKB and UKB

Pathogens	CKB	UKB
**Hepatitis virus**		
HBV[1]	41.4	2.6
HCV[1]	0.6	0.3
**Human Papillomavirus (HPV)**		
HPV-16[1]	5.8	4.9
HPV-18[1]	3.2	3.0
**Human Herpesvirus (HHV)**		
HHV-1	93.4	68.6
HHV-2	8.3	16.8
VZV	87.1	92.2
EBV[1]	95.1	94.8
CMV	92.7	55.5
HHV-6	59.7	90.8
HHV-7	81.6	95.0
**Human Polyomavirus (HPyV)**		
BKV	86.9	95.9
JCV	61.5	62.4
MCV	60.9	67.3
**Other viruses**		
HIV[1]	0.04	0.2
HTLV[1]	0.1	1.4
**Bacteria/Parasite**		
H. pylori[1]	50.5	34.1
C. trachomatis	44.1	23.1
T. gondii	13.1	26.7
**Number of any pathogen +**	**9.3 (1.7)**	**8.4 (1.9)**
**Number of oncogenic pathogens +**	**2.1 (0.8)**	**1.4 (0.7)**

Sero-prevalence for each infectious pathogen in CKB and UKB was directly standardized to WHO World Standard Population (available from https://seer.cancer.gov/stdpopulations/world.who.html)
[1] Oncogenic pathogen

For peer review only - http://bmjopen.bmj.com/site/about/guidelines.xhtml

Figure S1. Locations of the 10 survey sites and number recruited.

Open circles indicate rural areas and solid circles indicate urban areas. Number recruited at baseline in each area is shown in brackets.

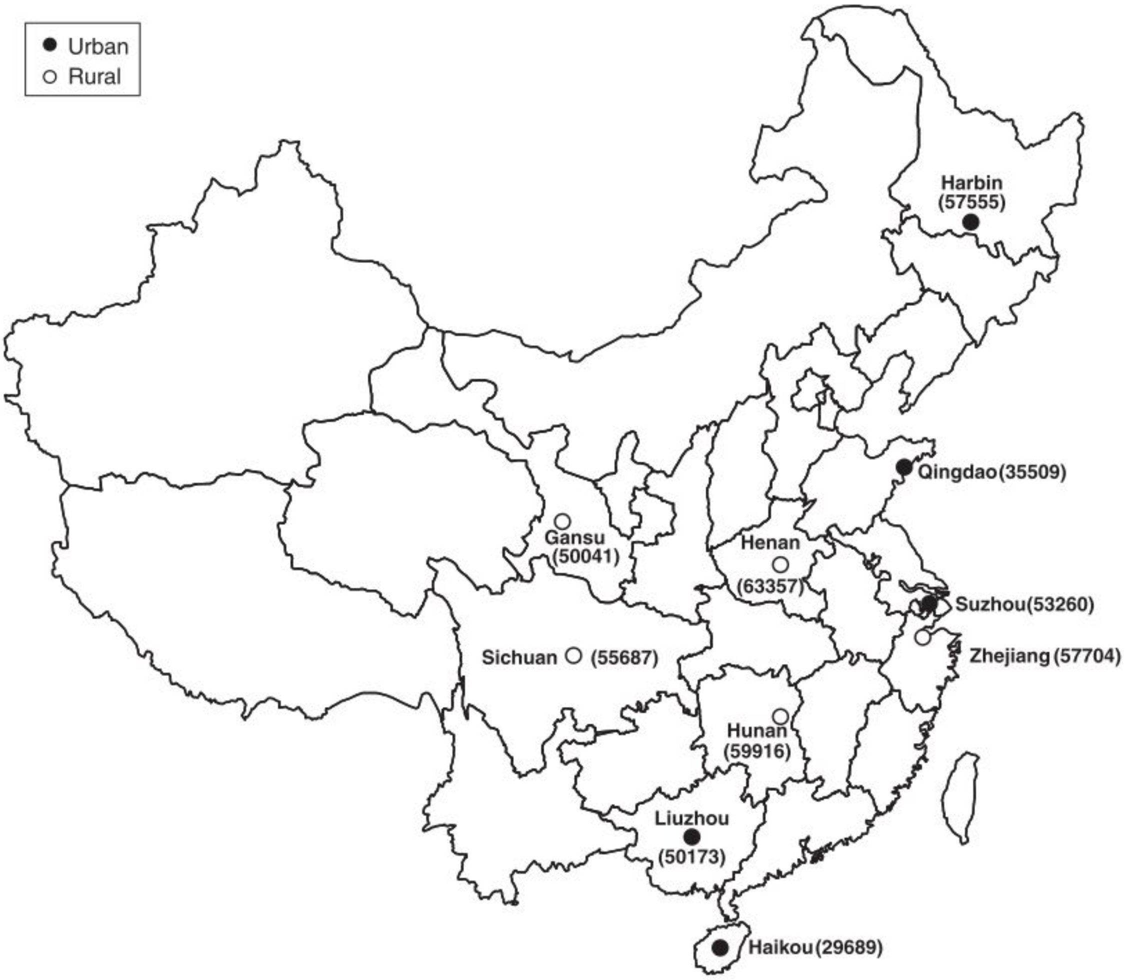

only

For peer review only - http://bmjopen.bmj.com/site/about/guidelines.xhtml

Figure S2. Distribution of median fluorescence intensity of 43 antigens

For each antigen, orange and blue represent sero-status of positive and negative, respectively.

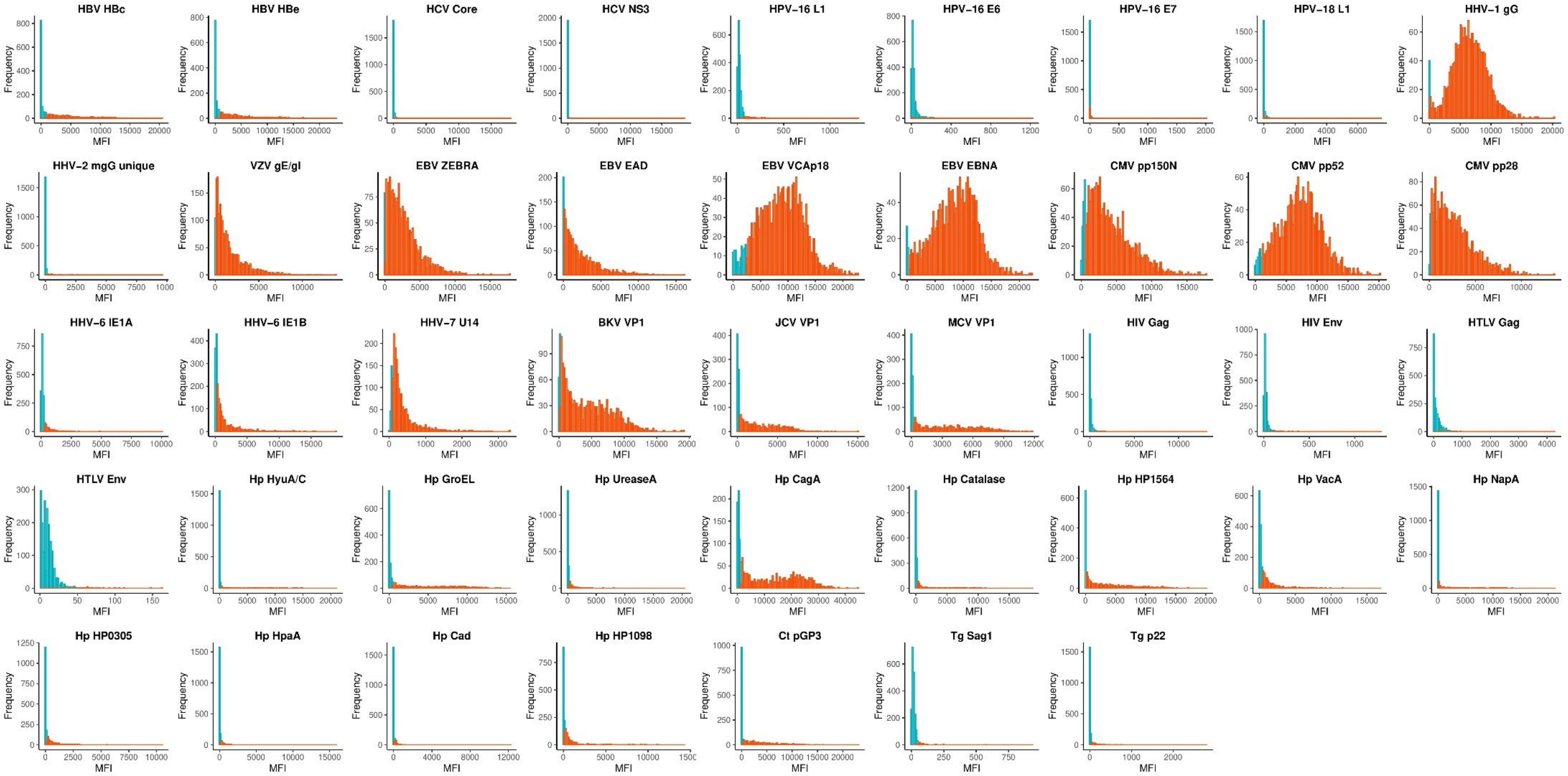

Figure S3. Correlation matrix of pathogens

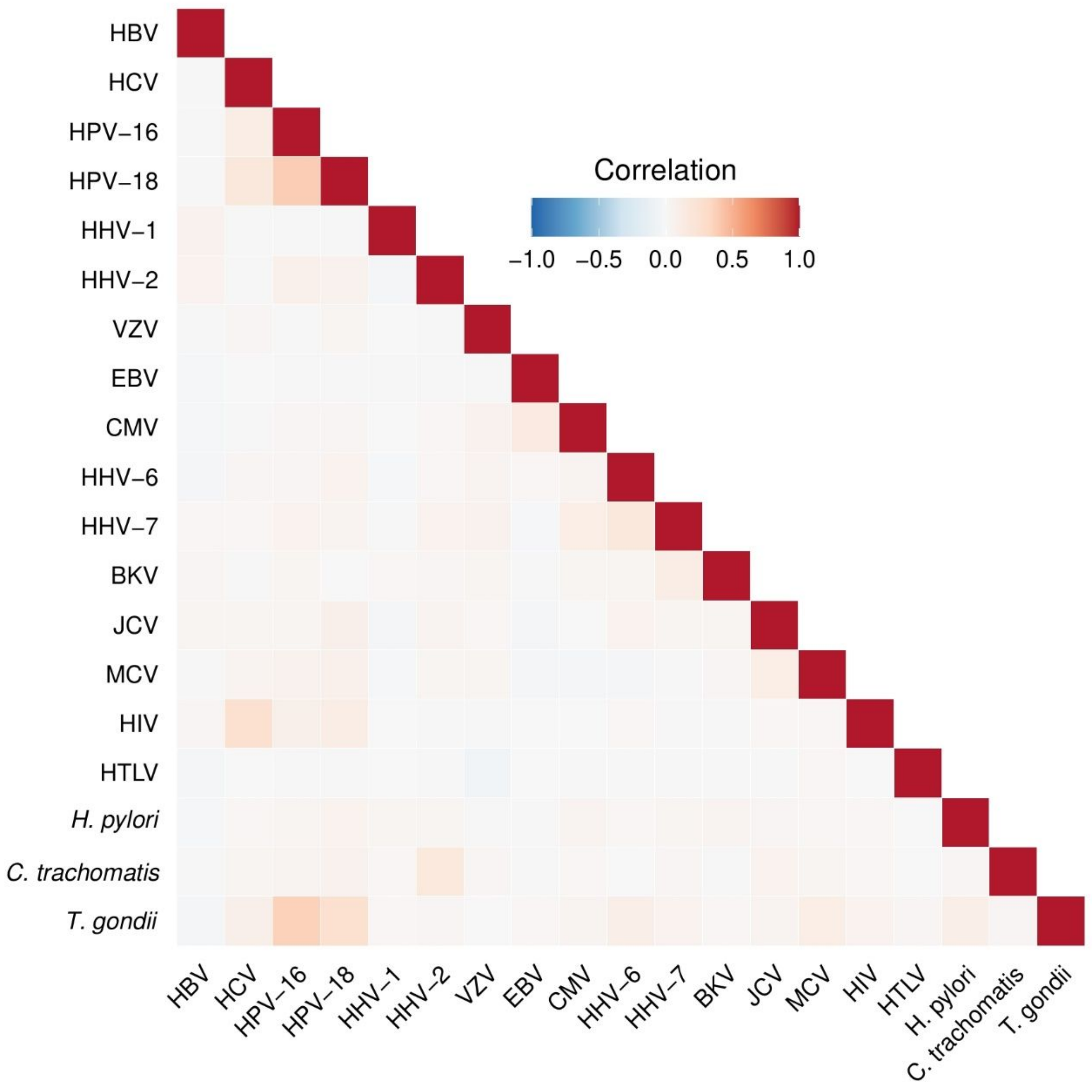

Figure S4. Correlation matrix of 43 antigens

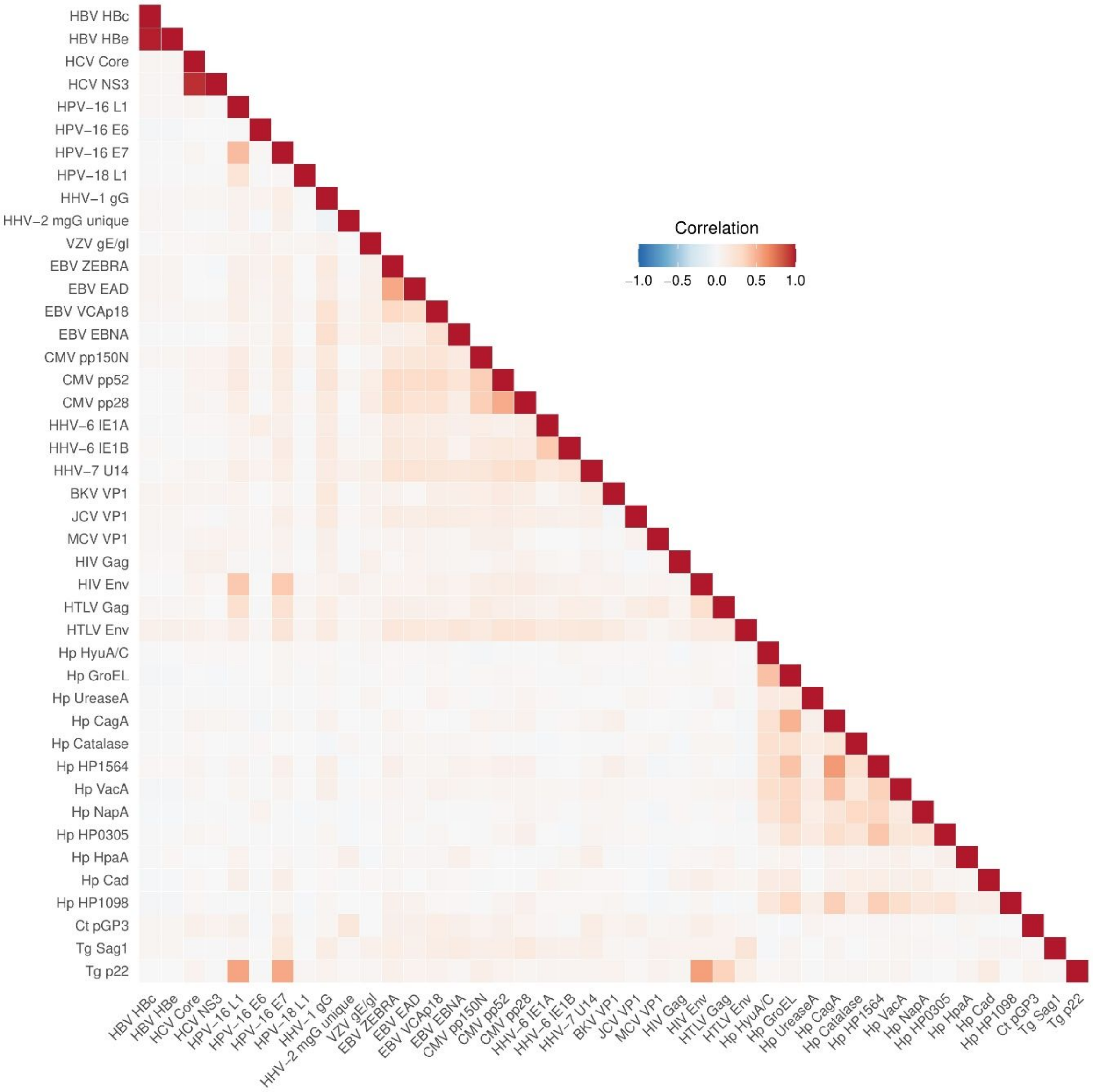

For peer review only - http://bmjopen.bmj.com/site/about/guidelines.xhtml

Figure S5. Intraclass correlation coefficient for MFI of 43 antigens between baseline and the 1st resurvey number of infections

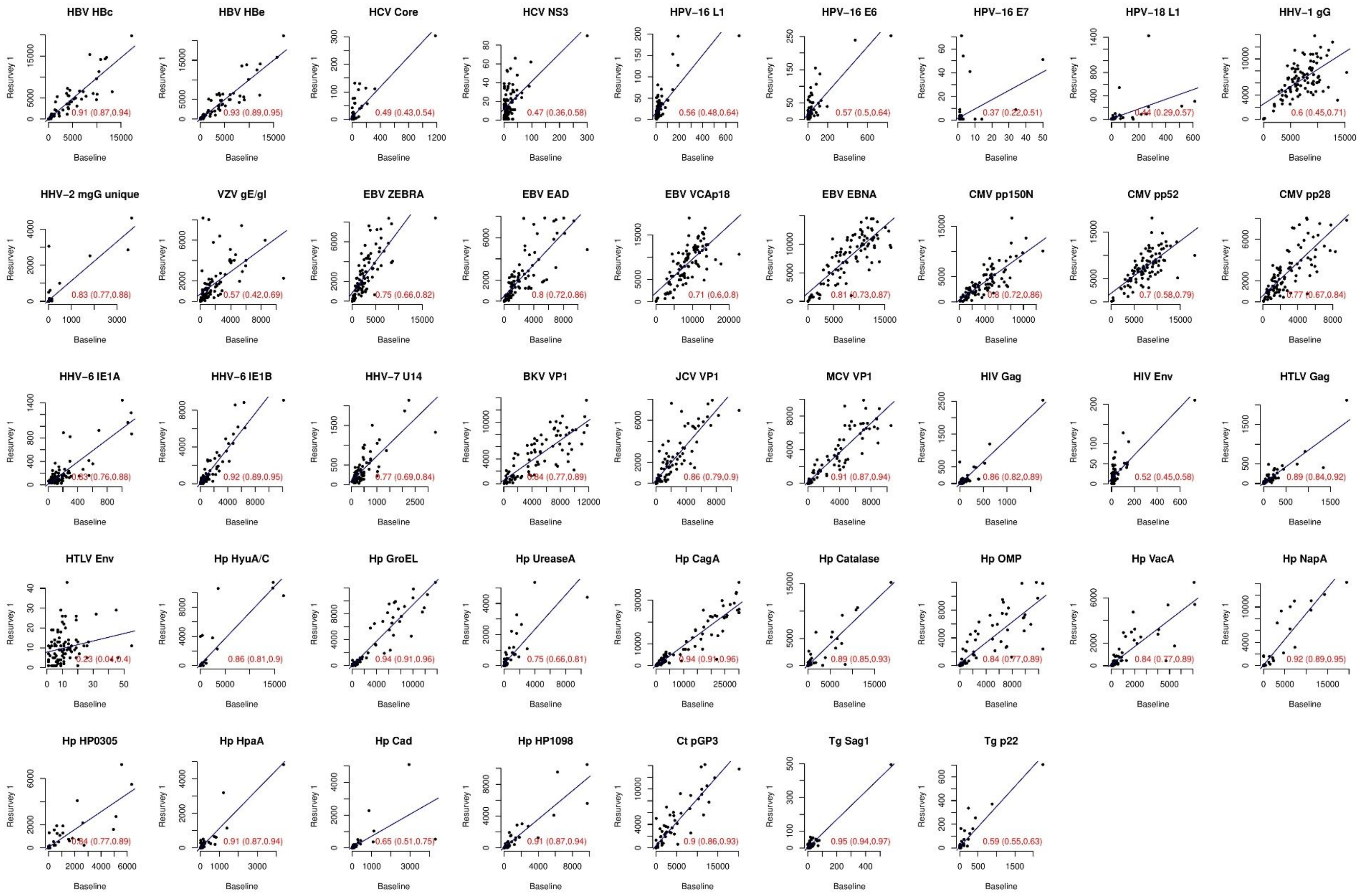

Figure S6. Intraclass correlation coefficient for MFI of 43 antigens between baseline and the 2nd resurvey

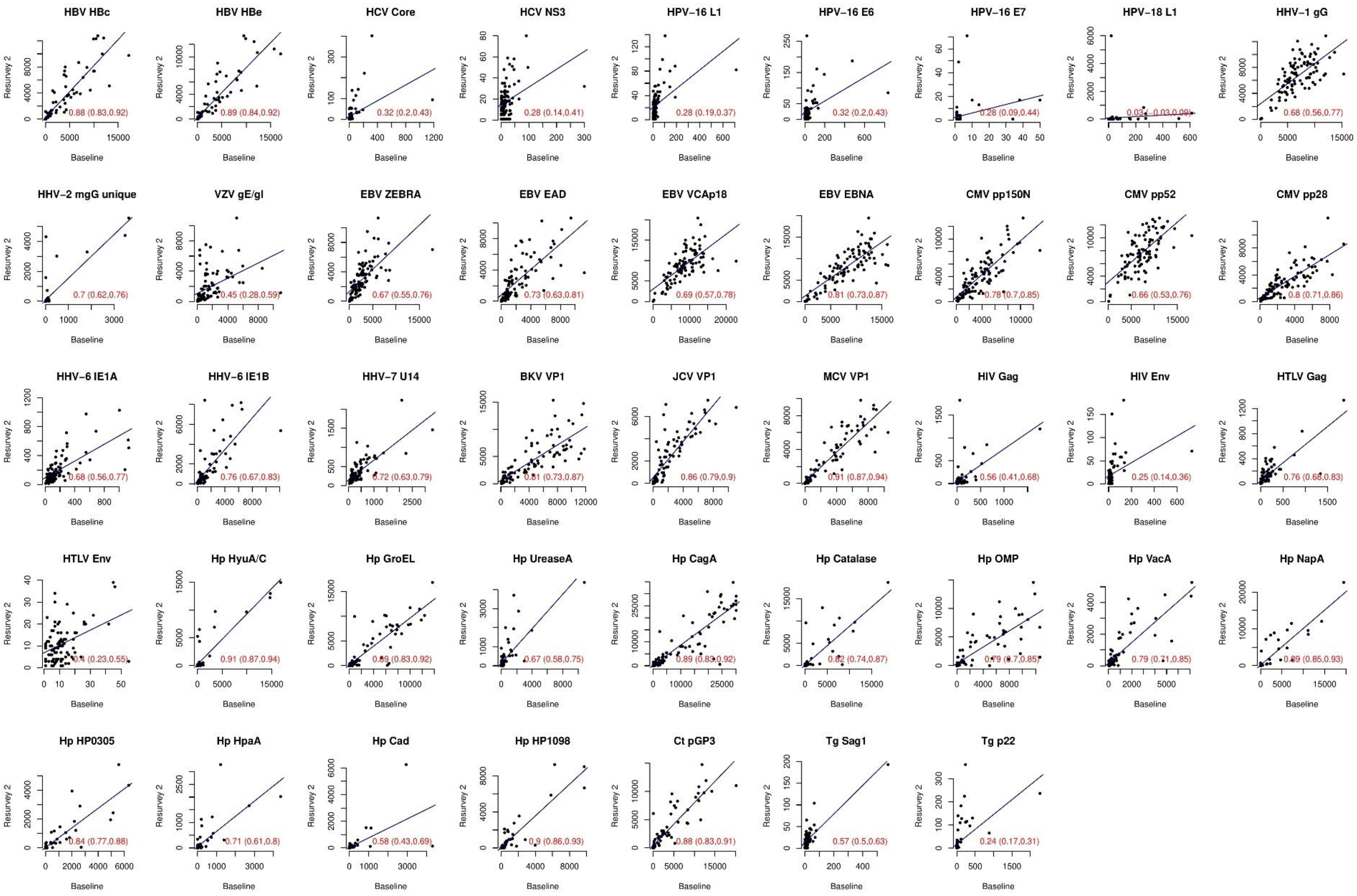

Figure S7. Intraclass correlation coefficient for MFI of 43 antigens between the 1st and the 2nd resurvey

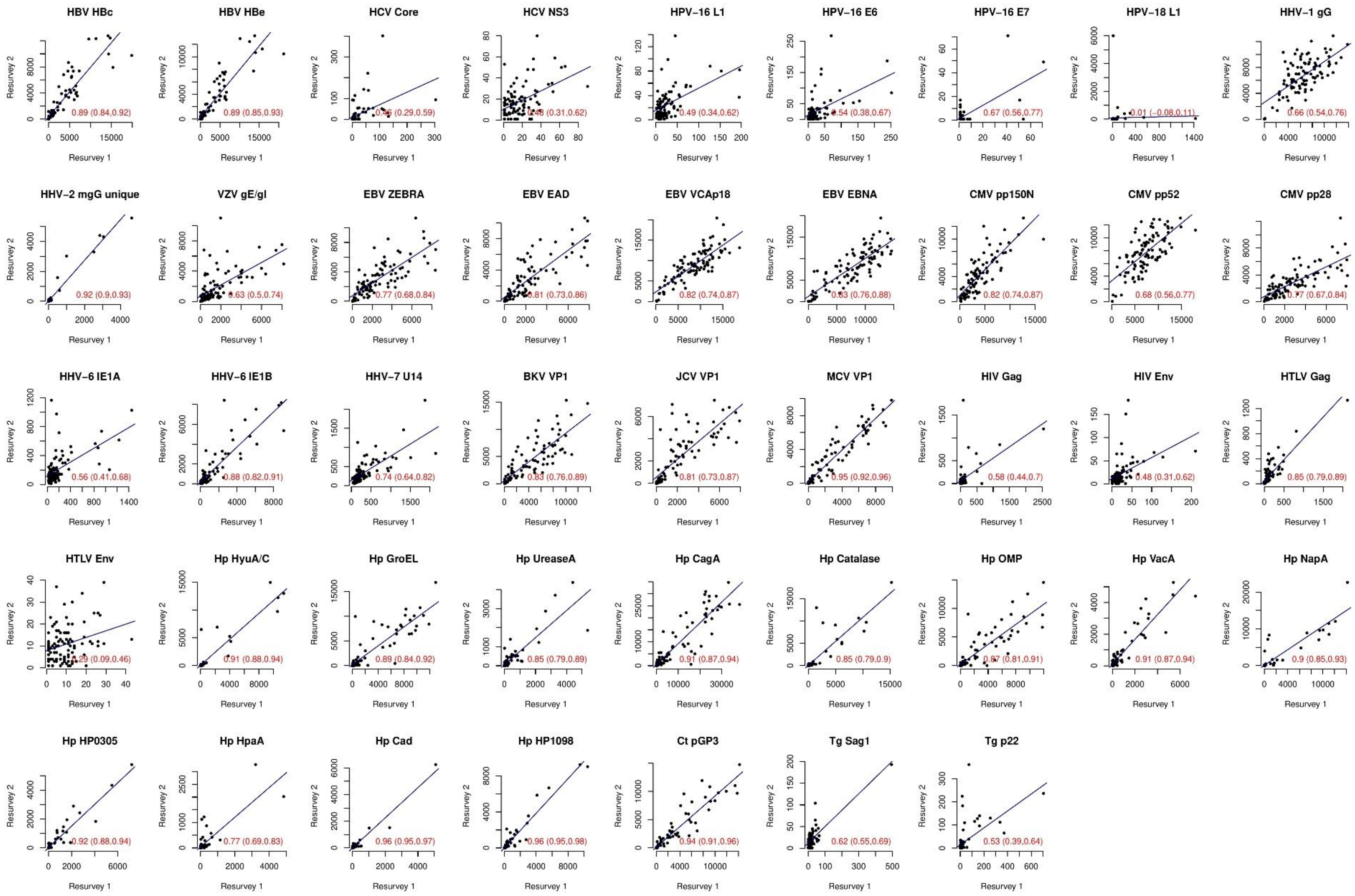

STROBE Statement—Checklist of items that should be included in reports of ***cross-sectional studies***

	Item No	Recommendation	Page No
**Title and abstract**	1	(*a*) Indicate the study's design with a commonly used term in the title or the abstract	1
		(*b*) Provide in the abstract an informative and balanced summary of what was done and what was found	1
**Introduction**			
Background/rationale	2	Explain the scientific background and rationale for the investigation being reported	4-5
Objectives	3	State specific objectives, including any prespecified hypotheses	5
**Methods**			
Study design	4	Present key elements of study design early in the paper	5
Setting	5	Describe the setting, locations, and relevant dates, including periods of recruitment, exposure, follow-up, and data collection	5
Participants	6	(*a*) Give the eligibility criteria, and the sources and methods of selection of participants	5
Variables	7	Clearly define all outcomes, exposures, predictors, potential confounders, and effect modifiers. Give diagnostic criteria, if applicable	5
Data sources/ measurement	8*	For each variable of interest, give sources of data and details of methods of assessment (measurement). Describe comparability of assessment methods if there is more than one group	5-6
Bias	9	Describe any efforts to address potential sources of bias	5-6
Study size	10	Explain how the study size was arrived at	5
Quantitative variables	11	Explain how quantitative variables were handled in the analyses. If applicable, describe which groupings were chosen and why	7
Statistical methods	12	(*a*) Describe all statistical methods, including those used to control for confounding	7
		(*b*) Describe any methods used to examine subgroups and interactions	7
		(*c*) Explain how missing data were addressed	
		(*d*) If applicable, describe analytical methods taking account of sampling strategy	7
		(*e*) Describe any sensitivity analyses	
**Results**			
Participants	13*	(a) Report numbers of individuals at each stage of study—eg numbers potentially eligible, examined for eligibility, confirmed eligible, included in the study, completing follow-up, and analysed	8
		(b) Give reasons for non-participation at each stage	
		(c) Consider use of a flow diagram	
Descriptive data	14*	(a) Give characteristics of study participants (eg demographic, clinical, social) and information on exposures and potential confounders	8
		(b) Indicate number of participants with missing data for each variable of interest	
Outcome data	15*	Report numbers of outcome events or summary measures	8-10
Main results	16	(*a*) Give unadjusted estimates and, if applicable, confounder-adjusted estimates and their precision (eg, 95% confidence interval). Make clear which confounders were adjusted for and why they were included	10

For peer review only

		(*b*) Report category boundaries when continuous variables were categorized	
		(*c*) If relevant, consider translating estimates of relative risk into absolute risk for a meaningful time period	
Other analyses	17	Report other analyses done—eg analyses of subgroups and interactions, and sensitivity analyses	10
**Discussion**			
Key results	18	Summarise key results with reference to study objectives	10-11
Limitations	19	Discuss limitations of the study, taking into account sources of potential bias or imprecision. Discuss both direction and magnitude of any potential bias	18
Interpretation	20	Give a cautious overall interpretation of results considering objectives, limitations, multiplicity of analyses, results from similar studies, and other relevant evidence	11-18
Generalisability	21	Discuss the generalisability (external validity) of the study results	11-18
**Other information**			
Funding	22	Give the source of funding and the role of the funders for the present study and, if applicable, for the original study on which the present article is based	19

*Give information separately for exposed and unexposed groups.

Note: An Explanation and Elaboration article discusses each checklist item and gives methodological background and published examples of transparent reporting. The STROBE checklist is best used in conjunction with this article (freely available on the Web sites of PLoS Medicine at http://www.plosmedicine.org/, Annals of Internal Medicine at http://www.annals.org/, and Epidemiology at http://www.epidem.com/). Information on the STROBE Initiative is available at www.strobe-statement.org.

For peer review only

BMJ Open

Sero-prevalence of 19 infectious pathogens and associated factors among middle-aged and elderly Chinese adults: a cross-sectional study

Journal:	*BMJ Open*
Manuscript ID	bmjopen-2021-058353.R1
Article Type:	Original research
Date Submitted by the Author:	21-Feb-2022
Complete List of Authors:	Yao, Pang; University of Oxford Nuffield Department of Population Health; University of Oxford Nuffield Department of Population Health, University of Oxford<br>Millwood, Iona; University of Oxford, Nuffield Department of Population Health; University of Oxford, Nuffield Department of Population Health<br>Kartsonaki, Christiana ; University of Oxford, Nuffield Department of Population Health<br>Mentzer, Alexander J; University of Oxford<br>Allen, Naomi; University of Oxford, Nuffield Department of Population Health<br>Jeske, Rima; Infections and Cancer Epidemiology Division, German Cancer Research Center<br>Butt, Julia; Division of Molecular Diagnostics of Oncogenic Infections, Infection, Inflammation and Cancer Program, German Cancer Research Center (DKFZ)<br>Guo, Yu; Chinese Academy of Medical Sciences<br>Chen, Yiping; University of Oxford, Nuffield Department of Population Health<br>Walters , Robin; University of Oxford, Nuffield Department of Population Health; University of Oxford, Nuffield Department of Population Health<br>Lv, Jun; Peking University Health Science Center, Epidemiology & Biostatistics<br>Yu, Canqing; Peking University Health Science Center, Epidemiology & Biostatistics<br>Plummer, Martyn; University of Warwick, Department of Statistics<br>de Martel, Catherine; International Agency for Research on Cancer<br>Clifford, Gary; International Agency for Research on Cancer<br>LI, Li-ming; Peking University School of Public Health, Epidemiology and Biostatistics<br>Waterboer, Tim; Division of Molecular Diagnostics of Oncogenic Infections, Infection, Inflammation and Cancer Program, German Cancer Research Center (DKFZ)<br>Yang, Ling; University of Oxford, Nuffield Department of Population Health; University of Oxford, Nuffield Department of Population Health<br>Chen, Zhengming; University of Oxford, Nuffield Department of Population Health
**Primary Subject Heading**:	Epidemiology

Secondary Subject Heading:	Infectious diseases
Keywords:	Epidemiology < INFECTIOUS DISEASES, PUBLIC HEALTH, INFECTIOUS DISEASES

SCHOLARONE™ Manuscripts

I, the Submitting Author has the right to grant and does grant on behalf of all authors of the Work (as defined in the below author licence), an exclusive licence and/or a non-exclusive licence for contributions from authors who are: i) UK Crown employees; ii) where BMJ has agreed a CC-BY licence shall apply, and/or iii) in accordance with the terms applicable for US Federal Government officers or employees acting as part of their official duties; on a worldwide, perpetual, irrevocable, royalty-free basis to BMJ Publishing Group Ltd ("BMJ") its licensees and where the relevant Journal is co-owned by BMJ to the co-owners of the Journal, to publish the Work in this journal and any other BMJ products and to exploit all rights, as set out in our licence.

The Submitting Author accepts and understands that any supply made under these terms is made by BMJ to the Submitting Author unless you are acting as an employee on behalf of your employer or a postgraduate student of an affiliated institution which is paying any applicable article publishing charge ("APC") for Open Access articles. Where the Submitting Author wishes to make the Work available on an Open Access basis (and intends to pay the relevant APC), the terms of reuse of such Open Access shall be governed by a Creative Commons licence – details of these licences and which Creative Commons licence will apply to this Work are set out in our licence referred to above.

Other than as permitted in any relevant BMJ Author's Self Archiving Policies, I confirm this Work has not been accepted for publication elsewhere, is not being considered for publication elsewhere and does not duplicate material already published. I confirm all authors consent to publication of this Work and authorise the granting of this licence.

For peer review only

Sero-prevalence of 19 infectious pathogens and associated factors among middle-aged and elderly Chinese adults: a cross-sectional study

Pang Yao[1], Iona Y. Millwood[1,2,] Christiana Kartsonaki[1,2], Alexander J Mentzer[3], Naomi Allen[1], Rima Jeske[4], Julia Butt[4], Yu Guo[5], Yiping Chen[1,2], Robin G. Walters[1,2], Jun Lv[6], Canqing Yu[6], Martyn Plummer[7], Catherine de Martel[8], Gary M. Clifford[8], Liming Li[6], Tim Waterboer[4, #], Ling Yang[1,2, #], Zhengming Chen[1, #]

[1] Clinical Trial Service Unit & Epidemiological Studies Unit (CTSU), Nuffield Department of Population Health, University of Oxford, Oxford, UK
[2] Medical Research Council Population Health Research Unit (MRC PHRU), Nuffield Department of Population Health, University of Oxford, Oxford, UK
[3] The Wellcome Centre for Human Genetics, University of Oxford, Oxford, UK
[4] Infections and Cancer Epidemiology Division, German Cancer Research Center (DKFZ), Heidelberg, Germany
[5] Fuwai Hospital Chinese Academy of Medical Sciences, National Center for Cardiovascular Diseases, Beijing, China
[6] Department of Epidemiology and Biostatistics, School of Public Health, Peking University Health Science Centre, Beijing, China
[7] Department of Statistics, University of Warwick, Coventry, UK
[8] Early Detection, Prevention and Infections Branch, International Agency for Research on Cancer, Lyon, France
[#] *co-senior authors*

Address for correspondence:

Ling Yang
MRC PHRU and CTSU
Nuffield Department of Population Health, Big Data Institute Building, University of Oxford,
Old Road Campus, Oxford, OX3 7LF, United Kingdom
Email: ling.yang@ndph.ox.ac.uk
Tel: +44 (0) 1865 743936

or

Iona Y Millwood
MRC PHRU and CTSU
Nuffield Department of Population Health, Big Data Institute Building, University of Oxford,
Old Road Campus, Oxford, OX3 7LF, United Kingdom
Email: iona.millwood@ndph.ox.ac.uk
Tel: +44 (0) 1865 743612

Word count: Abstract 247, Text 3849
2 Tables 3 Figures and 11 Supplementary Tables or Supplementary Figures

CKB Research Tracker No.: 2019-0052

17 February 2022

For peer review only

For peer review only - http://bmjopen.bmj.com/site/about/guidelines.xhtml

Abstract

Objectives: To systematically assess the sero-prevalence and associated factors of major infectious pathogens in China, where there are high incidence rates of certain infection-related cancers.

Design: Cross-sectional study.

Setting: 10 (5 urban, 5 rural) geographically diverse areas in China.

Participants: A subcohort of 2000 participants from the China Kadoorie Biobank.

Primary measures: Sero-prevalence of 19 pathogens using a custom-designed multiplex serology panel and associated factors.

Results: Of the 19 pathogens investigated, the mean number of sero-positive pathogens was 9.4 (SD 1.7), with 24.4% of participants being sero-positive for >10 pathogens. For individual pathogens, the sero-prevalence varied, being e.g. 0.05% for HIV, 6.4% for HPV-16, 53.5% for *H. pylori* and 99.8% for EBV. The sero-prevalence of HHV-6, HHV-7 and HPV-16 was higher in women than men. Several pathogens showed a decreasing trend in sero-prevalence by birth cohort, including HBV (51.6% vs 38.7% in those born <1940 vs >1970), HPV-16 (11.4% vs 5.4%), HHV-2 (15.1% vs 8.1%), *C. trachomatis* (65.6% vs 28.8%) and *T. gondii* (22.0% vs 9.0%). Across the 10 study areas, sero-prevalence varied 2 to 4-fold for HBV (22.5% to 60.7%), HPV-16 (3.4% to 10.9%), *H. pylori* (16.2% to 71.1%) and *C. trachomatis* (32.5% to 66.5%). Participants with chronic liver diseases had >7-fold higher sero-positivity for HBV (OR=7.51; 95% CI: 2.55-22.13).

Conclusions: Among Chinese adults, past and current infections with certain pathogens were common, and varied by area, sex and birth cohort. These infections may contribute to the burden of certain cancers and other non-communicable chronic diseases.

For peer review only - http://bmjopen.bmj.com/site/about/guidelines.xhtml

Strengths and limitations of this study

The present study simultaneously assessed antibodies against multiple pathogens, using a validated and custom designed multiplex serology platform.

The broad age range of participants also allowed assessment of differences in the pathogens' sero-prevalence across the different birth cohorts and/or age groups.

The multiplex serology measurements are designed to detect cumulative (i.e. past and present) exposure in a high-throughput setting and may partially explain the high sero-prevalence of several pathogens compared with previous estimates.

The information collected about possible determinants or correlates for certain infections was limited, particularly in relation to sexual behaviours.

Data availability statement

No data are currently available.

For peer review only

Introduction

Chronic infections with certain pathogens, including viruses, bacteria, and parasites, have been associated with increased risks of several non-communicable chronic diseases (NCDs), especially certain cancers.[1] In 2018, an estimated 2.2 million cancer cases worldwide were attributed to infections, mainly *Helicobacter pylori* (*H. pylori*; 810,000 cases), human papillomavirus (HPV; 690,000), hepatitis B virus (HBV; 360,000) and hepatitis C virus (HCV; 160,000).[1] The incidence rates of infection-related cancers were highest in eastern Asia, particularly China, owing to the high prevalence of *H. pylori* and HBV.[1] Apart from cancer, a growing body of evidence also suggests that inflammation induced by persistent pathogen infection may be associated with risks of cardiovascular disease (cytomegalovirus),[2] multiple sclerosis (Epstein-Barr virus [EBV])[3] and Alzheimer's disease (human herpesviruses [HHV] 6 and 7).[4] Moreover, there is also evidence suggesting that pathogen burden (i.e., the number of different infectious pathogens an individual has been exposed to) may contribute to atherosclerosis and risk of cardiovascular disease.[2]

The prevalence of pathogens tends to vary greatly between and within populations and is often associated with disparities in socioeconomic status, household crowding, breastfeeding practices, food-production practices and environmental factors such as climate.[5] Although many sero-epidemiological studies of major infectious pathogens have been undertaken,[6-9] they have tended to cover a single pathogen,[6-8] or involve populations in high-income countries.[9] In China, although several nationwide surveys have previously reported on the prevalence of some major pathogens (e.g. HBV, HCV),[10-12] there is limited contemporary data about the sero-prevalence of many other pathogens and their relevance for major NCDs beyond certain cancers.

For peer review only - http://bmjopen.bmj.com/site/about/guidelines.xhtml

Moreover, few studies in China or elsewhere have systematically examined the sero-prevalence of multiple pathogens in the same population, which could provide important data about pathogen burden and the joint effects of different pathogens on disease aetiology.

The present study examined the sero-prevalence of 19 infectious pathogens and investigated certain demographic factors associated with sero-positivity among 2000 adults from the China Kadoorie Biobank (CKB) study that covered 10 geographically diverse urban and rural areas in China.

Materials and Methods

Study population

Details of the CKB study design, methods, and participants have been previously reported.[13] The 512,715 participants aged 30-79 years were enrolled from 10 (5 urban, 5 rural) geographically diverse regions in China (**Supplementary Figure 1**). At baseline (June 2004-July 2008) and at two subsequent resurveys in a 5% subset (in 2008 and 2013, respectively), detailed data were collected on socio-demographic characteristics, smoking, alcohol consumption, diet, physical activity, personal and family medical history, and physical measurements. Follow-up of CKB participants was through linkage to established mortality and morbidity registries, and to the nationwide health insurance system which records all hospitalised episodes. Ethics approval was obtained from the Oxford University, the China National CDC, and also from institutional research boards at the local CDCs in the 10 areas.[13] All participants provided written informed consent. The present study population was a subcohort of 2000 participants from a case-subcohort study of gastric and oesophageal cancers in CKB. This subcohort was selected using simple random sampling from those alive

For peer review only

with no history of cancer 2 years after entering the study who had an available plasma sample collected at baseline and had genotyping data available as part of a population-based subset.

Patient and public involvement

No patients or population groups were involved.

Blood sample collection and processing

At baseline, a 10 ml non-fasting venous blood sample was collected from each participant into an EDTA vacutainer (with time of last meal recorded). Samples were separated into plasma and buffy coat aliquots, and frozen within 24 hours of collection (mean delay ~10 hours). Two aliquots of plasma from each blood sample were shipped to Oxford, UK for long-term storage in liquid nitrogen following the formal approval of regulatory agencies in China.

Multiplex serology

The multiplex serology panel used in the present study was based on that developed for the UK Biobank,[9] with certain relevant modifications. More *H. pylori* antigens were included in the CKB panel and a few pathogens were removed taking their prevalence into account.[9] The CKB panel measured antibody levels against 43 antigens from 19 infectious pathogens (see details in Supplementary Table 1) including hepatitis B (HBV) and hepatitis C (HCV) viruses; human papillomaviruses 16 (HPV-16) and 18 (HPV-18); herpes simplex viruses 1 and 2 (HHV-1 and HHV-2); varicella zoster virus (VZV); Epstein-Barr virus (EBV); human cytomegalovirus (CMV); human herpesviruses 6 and 7 (HHV-6 and HHV-7); BK (BKV), JC (JCV) and Merkel cell (MCV) polyomaviruses; human immunodeficiency virus (HIV); human T

For peer review only - http://bmjopen.bmj.com/site/about/guidelines.xhtml

lymphotropic virus (HTLV); *Helicobacter pylori (H. pylori)*; *Chlamydia trachomatis (C. trachomatis)*; and *Toxoplasma gondii (T. gondii)*. Of these 19 pathogens, 8 (HBV, HCV, HIV, HTLV, *H. pylori*, HPV-16, HPV-18 and EBV) are classified as Group I human carcinogens by the IARC.

The quantitative multiplex antibody detection approach was based on a glutathione S-transferase (GST) capture enzyme-linked immunosorbent assay combined with fluorescent-bead technology, with various validation procedures.[14] Antigens of infectious pathogens were bacterially expressed as GST-X-tag fusion proteins and coupled to spectrally distinguishable glutathione-casein covered beads. The median fluorescence intensity (MFI) of at least 100 beads per antigen was measured, and thus a sample was defined sero-positive for specific antibody against a corresponding antigen if the respective MFI value exceeded the antigen-specific cut-off. Antigen-specific cut-offs for *H. pylori* antigens were applied as described previously,[15] and were quality assured by the visual inflection point method.[16] To define overall sero-positivity for a pathogen, antigen-specific sero-positivity results were subsequently combined using published algorithms (see details in Supplementary Table 1 and Supplementary Figure 2).[9]

For 100 (5%) of these participants, plasma samples collected at the two subsequent resurveys were also assayed to assess the consistency of sero-status (sero-conversion and sero-reversion rates) measurements over a period of time.

Statistical analysis

Sero-prevalence estimates and 95% confidence intervals (CIs) for each antigen and pathogen were calculated. For pathogens, sero-prevalence was also estimated in subgroups defined by sex, birth cohort (each year) and study area (10). Locally

For peer review only - http://bmjopen.bmj.com/site/about/guidelines.xhtml

weighted scatterplot smoothing (LOESS) curves were used to visualise the relationship between birth cohort and sero-prevalence of each pathogen, and proportion of individuals with >10 infectious pathogens, or >2 oncogenic pathogens. Logistic regression was used to estimate odds ratios (ORs) by pathogen sero-positivity for various baseline characteristics, adjusting for age, sex and area. Pathogens with extreme sero-prevalence (i.e., <5% or >95%) were excluded from the association analyses. Continuous MFI values were used to compute Pearson correlation coefficients between each of the antigens. Spearman's correlation coefficients were calculated between pathogens. The analysis was done using R version 3.6.2.

Results

Characteristics of the study population

Among the subcohort of 1986 participants (14 samples were excluded due to technical errors) included in the main analyses, the mean (SD) age was 51.8 (10.8) years, 61.7% were women, and 50.3% were urban residents, which, along with many other baseline characteristics, were similar to those in the overall CKB cohort (**Table 1**).

Sero-prevalence of pathogens

Of the 19 pathogens investigated, the sero-prevalence estimates ranged from 0.05% to 99.8% for individual pathogens (**Table 2**). Within specific families of pathogens, the sero-prevalences also varied greatly. For the hepatitis viruses, the sero-prevalence was 43.8% for HBV and 0.7% for HCV. Among papillomaviruses, HPV-16 sero-prevalence was higher (6.4%) than HPV-18 (3.4%). The sero-prevalence of

For peer review only - http://bmjopen.bmj.com/site/about/guidelines.xhtml

herpesviruses was 98.0% for HHV-1, 8.9% for HHV-2, 91.5% for VZV, 99.8% for EBV, 97.3% for CMV, 62.0% for HHV-6, and 85.5% for HHV-7. Among the polyomaviruses, BKV had the highest sero-prevalence (91.1%), followed by JCV (65.6%) and MCV (64.9%). For HIV and HTLV, however, only samples from 1 (0.05%) and 2 (0.1%) participants tested positive, respectively. The sero-prevalences of *H. pylori, C. trachomatis,* and *T. gondii* were 53.5%, 47.6%, and 14.2% respectively.

No differences by sex were observed for most pathogens but, compared with men, women had a higher sero-prevalence of HPV-16 (7.9% vs 4.1%), HHV-6 (68.0% vs 52.3%) and HHV-7 (89.3% vs 79.5%). A decreasing trend in sero-prevalence by birth cohort was seen for HBV (51.6% in those born before 1940 to 38.7% in those born after 1970), HPV-16 (11.4% to 5.4%), HHV-2 (15.1% to 8.1%), JCV (75.5% to 61.3%), MCV (73.6% to 62.2%), *C. trachomatis* (65.6% to 28.8%), and *T. gondii* (22.0% to 9.0%), while no clear trends were evident for other pathogens, except HHV-6 which showed an upward trend (**Figure 1**). Across the 10 study areas, there were at least 2-fold differences in sero-prevalence for HBV (22.5% to 60.7%), HPV-16 (3.4% to 10.9%), HHV-2 (4.3% to 17.8%), *H. pylori* (16.2% to 71.1%), *C. trachomatis* (32.5% to 66.5%) and *T. gondii* (8.0% to 18.4%) (**Figure 2**).

Among the 1986 participants and 19 pathogens tested, all participants were sero-positive for at least 1 pathogen and 24.4% were sero-positive for more than 10 pathogens, with the mean number being 9.4 (SD, 1.7) (**Figure 3**). The mean number of oncogenic pathogens for which a participant was sero-positive was 2.1 (SD, 0.8) out of 8 oncogenic pathogens. Overall, there was a clear decreasing trend by birth

For peer review only - http://bmjopen.bmj.com/site/about/guidelines.xhtml

cohort in the proportions of participants sero-positive for >10 pathogens, or >2 oncogenic pathogens (**Figure 3**).

Factors associated with pathogen sero-positivity

The associations between sero-positivity of three major oncogenic pathogens (*H. pylori*, HPV-16, and HBV) and demographic and lifestyle factors are shown in **Supplementary Table 2**. Sero-positivity for HBV was more common in older participants (P_{trend} =0.05), those with cirrhosis or chronic hepatitis (adjusted OR=7.51, 2.55-22.13), and those who were hepatitis B surface antigen (HBsAg; measured using a point-of-care test) positive (11.51, 5.16-25.7) at baseline. The factors significantly associated with HPV-16 infection were older age (P_{trend} =0.02), being female (2.07, 1.36-3.14), higher levels of education (1.63, 1.01-2.65) or low levels of physical activity (0.50, 0.32-0.78), having diabetes (2.69, 1.52-4.77) and obesity/overweight status (1.51, 1.03-2.22). For *H. pylori*, urban residence was associated with higher sero-prevalence (1.80, 1.50-2.15), and the converse was observed for a history of peptic ulcer (0.52, 0.32-0.85).

For the human herpesvirus family, women were more likely to be sero-positive for HHV-2 (1.45, 1.04-2.03), HHV-6 (1.92, 1.59-2.31), and HHV-7 (2.15, 1.67-2.76) than men. Higher physical activity levels were associated with lower likelihood of VZV sero-positivity while participants who reported self-rated poor health were less likely to be HHV-7 sero-positive. For JCV, diabetes and central obesity were associated with lower likelihood of sero-positivity, while for *C. trachomatis*, higher levels of education and income were associated with lower, and diabetes was associated with higher sero-prevalence.

Correlation between different pathogens

For peer review only - http://bmjopen.bmj.com/site/about/guidelines.xhtml

Correlations between the 19 different pathogens were generally low (**Supplementary Figure 3**). However, for certain pathogens, there were high correlations between their different antigens, e.g. HBc and HBe (r=0.99; p<0.001) for HBV; core antigen and NS3 (r=0.95; p<0.001) for HCV, and HP1564 and CagA (r=0.60; p<0.001) for *H. pylori* (**Supplementary Figure 4**). For certain different pathogens, moderate correlations were also observed between certain antigens, such as HPV-16 L1 and *T. gondii* p22 (r=0.56; p<0.001).

Repeat measurements

The MFI values were moderately to highly correlated between the baseline survey, and the 1st and 2nd resurveys (**Supplementary Figure 5-7**). Overall, the mean (SD) ICC for 43 antigens was 0.76 (0.18) between baseline and the 1st resurvey (mean SD duration of 2.5 [0.7] years). The apparent sero-conversion and sero-reversion rates were generally low (ranging from 0%-11.3% for different pathogens) across surveys (**Supplementary Table 3**).

Discussion

In this large study of multiple pathogens in the general adult Chinese population, there were large differences in the sero-prevalence of different pathogens, both overall and by sex, birth cohort and areas. Moreover, certain socioeconomic factors (e.g. levels of education and income), lifestyle factors (e.g. physical activity) and medical history (e.g., having cirrhosis or chronic hepatitis) were also shown to be associated with sero-prevalence of many pathogens. Among the study population and the 19 pathogens tested, the mean number of sero-positive pathogens was more than 9.

For peer review only

For peer review only - http://bmjopen.bmj.com/site/about/guidelines.xhtml

The present study shows there is a substantial burden of past and current infections with various types of pathogens among Chinese adults. Compared with data from a similar study in the UK population involving about 10,000 adults (56% women),[9] certain infections were more prevalent in Chinese adults, particularly HBV, HHV-1, CMV, *H. pylori* and *C. trachomatis*, while others were less common, including HHV-2, HHV-6, HHV-7 and *T. gondii* (**Supplementary Table 4**). Overall, the mean number of sero-positive pathogens was slightly greater in Chinese than in UK adults (any pathogen 9.4 vs 8.4; oncogenic pathogens: 2.1 vs 1.4), suggesting that the overall burden of pathogen infections might be generally higher in Chinese populations.

HBV and HCV

For HBV, we measured antibody responses for HBc and HBe which indicate past and current natural HBV infection, respectively, and the rate was approximately 44% in the present study, much higher than the 3% reported in the UK Biobank.[9] Our sero-prevalence estimate was, however, lower than recent data (54.7%) from a much smaller study (214 controls in a nested case-control study, samples obtained from 1996-2000) in China and Singapore using the same antigens (HBc and HBe) measured using multiplex serology.[17] Several Chinese nationwide surveys indicated that the prevalence of chronic HBV infection among adults measured by hepatitis B surface antigen (HBsAg) had decreased steadily over the past two decades, from 10% in 1992,[10] to 7% in 2006,[11] and to 6% in 2010-2012.[12] In line with previous findings,[10-12] we also observed a declining sero-prevalence of HBV across birth cohorts, which was not influenced by the universal HBV vaccination program that was implemented about 20 years ago and restricted to new-borns. Our finding of a sero-prevalence of HCV (0.7%) is consistent with recent report from China using a

For peer review only - http://bmjopen.bmj.com/site/about/guidelines.xhtml

similar assay (0.9%) based on a smaller sample size (n=214),[17] and higher than the 0.3% sero-prevalence in the UK Biobank.[9] Our study showed that HBV sero-positivity was >7-fold higher in participants who had reported prior cirrhosis or chronic hepatitis than in their counterparties. This is not surprising, since both HBV and HCV can lead to progressive liver cirrhosis, fibrosis, and/or hepatocellular carcinoma and thus, have been classified as group I human carcinogens for hepatocellular carcinoma.

HPV-16 and HPV-18

The two most common HPVs, HPV-16 and HPV-18 are sexually transmitted oncogenic viruses, which account for 70-75% of all cervical cancers and 40-60% of cervical cancer precursors.[18] In addition to sex differences, HPV sero-prevalence may be affected by differences in socioeconomic conditions, cultural diversity, assay methods and survey period. In the present study, the sero-prevalences of HPV-16 (overall 6.4%, urban 7.0%, rural 5.9%) and HPV-18 (overall 3.4%, urban 3.5%, rural 3.2%) were comparable to those reported in Chinese living in Taiwan (n=1702; 7.6% and 3.9%, respectively) measured using HPV virus-like particle ELISA,[19] but somewhat lower than those reported in another study of 3790 (median age of 44 years, 58% women) rural Chinese from Central China (9.9% and 8.9%, respectively) measured using multiplex serology.[20] A recent meta-analysis involving 24 Chinese provinces and autonomous regions indicated that HPV-16 and HPV-18 were more prevalent in Central and Northwest China than the rest of the country.[21] The increasing trend between HPV-16 sero-prevalence and age was consistent with previous findings,[7 19] which may reflect the fact that cumulative exposure measured by HPV sero-positivity is higher among older individuals and that antibodies to HPV-16 tend to be persistent.[7] As in the present study, many previous studies have

For peer review only

consistently reported a higher sero-prevalence of HPV-16 in women than men.[19] It is possible that men are either not as susceptible to HPV-16 infection or more frequently able to clear the infection spontaneously without developing a systemic antibody response. Previous meta-analyses have reported that the overall HPV viral DNA prevalence among Chinese women ranged from 13.1% to 18.8%.[21] Targeted HPV vaccination and scale-up of cervical cancer screening for women are priorities in curbing the HPV epidemic in China.

Human herpesviruses

Herpesviruses are a large family of enveloped DNA viruses that are ubiquitous in humans and characteristically establish lifelong latent infection within host cells and subsequently reactivate under certain conditions. Eight herpesvirus types are known to infect humans (HHV-1 to HHV-8), and both primary infection and reactivation can lead to a wide spectrum of clinical manifestations, such as encephalitis, congenital defects and cancer. EBV is causally associated with various types of lymphoma and nasopharyngeal cancer, and has been classified as Group I human carcinogen by IARC.[22] This study shows that the age-standardized sero-prevalence of HHV-1 and CMV (HHV-5) is much higher in China (93.4% and 92.7%) than the UK (68.6% and 55.5%) or the US (48.1% and 50.4%), while HHV-2 is lower in China (8.3%) than the UK (16.8%) or the US (12.1%).[9] Similar ethnic differences were also observed within the UKB population,[9] with Asians (77.9% and 92.0%; n=236) having a higher sero-prevalence of HHV-1 and CMV than white participants (69.1% and 56.5%; n=9140). Moreover, we also observed 4-fold (4.3~17.8%) geographical differences for HHV-2 across the 10 areas in this study. Limited studies conducted in China have demonstrated HHV-2 sero-prevalence as high as 13.2% among 2141 rural residents

For peer review only - http://bmjopen.bmj.com/site/about/guidelines.xhtml

(5-60 years) of Zhejiang province in 2006,[23] and as low as 3.4% among a general population of 8074 residents (18-49 years) of Shandong province in 2016, with rates typically higher in women than men.[8] HHV-1, CMV and EBV are ubiquitous herpesviruses that usually are transmitted by contact with infected saliva or breastfeeding, whereas HHV-2 is almost always transmitted through sexual contacts.[24] Ethnic and geographical differences in sero-prevalences might be explained by differences in behaviour that facilitate transmission.[25] For example, a multi-ethnic population-based prospective cohort study of 4464 Dutch children reported that lifestyle factors including low family net household income, low maternal educational level and crowding could explain up to 48% of the ethnic differences in HHV-1 sero-prevalences, and up to 39% of the ethnic differences in EBV sero-prevalences.[25]

Human polyomaviruses

Polyomaviruses are potentially tumorigenic in humans. The observed sero-prevalences of BKV (91.1%), JCV (65.6%), and MCV (64.9%) in the present study was similar to those reported previously among Chinese using multiplex serology (206 controls with mean age of 58 years: BKV 91.3%, JCV 73.3% and MCV 67.0%; 5548 participants with mean age of 61 years: MCV 61.0%).[26 27] Limited data exist on the individual characteristics that relate to polyomaviruses sero-positivity in Chinese populations. An increasing sero-prevalence of JCV and MCV with age was found among Chinese or US populations, and may be explained by a combination of continuous transmission of the pathogen throughout life and persistent antibody responses.

H. pylori

For peer review only - http://bmjopen.bmj.com/site/about/guidelines.xhtml

Previous studies have reported a substantial regional variation in *H. pylori* prevalence globally with China having a relatively high prevalence. In a meta-analysis of 670,572 participants from 98 Chinese studies during 1983-2018, using different methods to measure *H. pylori*, 49.6% overall (ranging from 20.6% to 81.8%) were sero-positive for *H. pylori*.[28] The present study also observed a significantly higher age-standardized sero-prevalence in China than UK (50.5% vs. 34.1%) using multiplex serology (Supplementary Table 4). Within China, regional differences in *H. pylori* sero-prevalence have not been previously explored and we found substantial variation across the 10 study areas, which may be attributed partly to levels of socio-economic development and sanitation, as *H. pylori* is commonly transmitted person-to-person by saliva, bouts of gastroenteritis within households and/or spread by fecal contamination of food or water.[29 30] There is a good evidence that a combination of untreated water, crowded conditions, and poor hygiene contributes to higher *H. pylori* prevalence in developing countries.[28 31] However, we were not able to explore these factors comprehensively due to the limited information available, but, as in many other populations, we observed a declining or plateaued sero-prevalence of *H. pylori* by birth cohort.[32] Sero-positivity for *H. pylori* was less common in participants with a history of peptic ulcer, which may reflect reverse causation as individuals with peptic ulcer would be treated by antibiotics to eradicate *H. pylori*, leading to lower sero-prevalence.

C. trachomatis

C. trachomatis is the most commonly diagnosed bacterial sexually transmitted infection (STI) worldwide. Since *C. trachomatis* has not yet been included as a reportable STI in the national STI surveillance program in China, detailed

For peer review only - http://bmjopen.bmj.com/site/about/guidelines.xhtml

epidemiological data is lacking. Previous population-based studies reported a *C. trachomatis* current infection prevalence of 2.1-4.1% in Chinese,[6] while in the present study which measured current and past infection status prevalence was 47.6% overall, which was higher in older participants likely due to cumulative exposure of the infection. Although both HHV-2 and *C. trachomatis* are sexually transmitted, there was a 5-fold difference in sero-prevalence between these two pathogens in CKB (8.3% vs 44.1%), which was not evident in the UKB population (16.8% vs 23.1%). This is unexplained and warrants further investigation. Individuals with higher socioeconomic status were less likely to be infected, which was also reported in a national stratified probability sample of 3426 Chinese (aged 20 to 64 years, 49.3% women) in 1999-2000.[6]

T. gondii

T. gondii is a zoonotic parasite infecting all warm-blooded animals. Usually, infected humans are asymptomatic, but in pregnant women the infection may lead to abortion, stillbirth or other serious consequences in newborns. Based on two national serological surveys in 15 provinces in China, the ELISA-based sero-prevalence of *T. gondii* increased from 5.2% in 1988-1992 to 7.9% in 2001-2004.[33 34] A recent serological survey involving 2634 healthy individuals in China showed a 12.5% anti-*T. gondii* IgG positive rate,[35] which is comparable to our estimate (14.2%). Consistent with previous findings in China, we found the sero-prevalence was highest in Liuzhou (18.4%), Guangxi province, where the consumption of raw or undercooked meat is popular among local residents, which is an important factor in the prevalence of food-borne parasitic diseases.[36]

Strengths & Limitations

For peer review only

The main strength of the present study is the simultaneous assessment of antibodies against multiple pathogens, using a validated and custom designed multiplex serology platform. Moreover, the broad age range of participants also allowed assessment of differences in the pathogens' sero-prevalence across the different birth cohorts and/or age groups, in addition to associations with socioeconomic and lifestyle factors. Nevertheless, the study also has limitations. These findings may not be generalizable to general populations in China. Geographic variation in the sero-prevalence of pathogens was observed among the ten diverse regions of China that were included in the CKB study. However, with only ~200 participants from each region, the sero-prevalence in that area may not be accurately estimated. The multiplex serology measurements are designed to detect cumulative (i.e. past and present) exposure in a high-throughput setting and may partially explain the high sero-prevalence of several pathogens compared with previous estimates.[10-12] Furthermore, the information collected about possible determinants or correlates for certain infections was limited, particularly in relation to sexual behaviours. Finally, the number of participants tested did not allow us to explore prospectively the associations of the sero-prevalence of multiple pathogens with the incidence of cancers and other NCDs.

Conclusions

In summary, among Chinese adults, the burden of pathogen infections was high and varied by area, sex, birth cohort and certain socioeconomic and lifestyle factors. While some infections may represent past or transient infections with little or no major health consequences, others may represent chronic and persistent infections, which may lead to increased risk of certain cancers and many other NCDs. The

For peer review only - http://bmjopen.bmj.com/site/about/guidelines.xhtml

357 development of a custom designed multiplex panel and its application in CKB should
358 provide a reliable basis for future well-powered and prospective investigations of the
359 associations between chronic infections and consequent disease outcomes in the
360 Chinese population.

For peer review only

For peer review only - http://bmjopen.bmj.com/site/about/guidelines.xhtml

Contributors

PY, IM, CK, LY and ZC had full access to the data in the study and take responsibility for the integrity of the data and the accuracy of the data analysis. RJ, JB and TW conducted the assay measurement in the DKFZ Laboratory. Statistical analysis: PY. Drafting of the manuscript: PY. Acquisition, analysis, or interpretation of data: PY, IM, TW, LY and ZC. Critical revision of the manuscript for important intellectual content: PY, IM, CK, AM, RJ, JB, MP, CM, GC. Administrative, technical, or material support: NA, YG, YC, RW, JL, CY, and LL. Supervision: TW, LY and ZC. TW, LY and ZC contributed equally to this report.

Declaration of interests

We declare that we have no conflict of interest.

Data sharing

Anonymised baseline, resurvey, and cause-specific mortality and morbidity data are available for access through a formal application in CKB website (www.ckbiobank.org). The application will then be reviewed by a Data Access Committee. Further details about access policy and procedures can be found online at www.ckbiobank.org.

Funding statement

This work was supported by CRUK PRC-Project Award (C56488/A24504). The China Kadoorie Biobank (CKB) baseline survey was supported by the Kadoorie Charitable Foundation, Hong Kong. CKB long-term follow-up was supported by grants from the UK Wellcome Trust (212946/Z/18/Z, 202922/Z/16/Z, 104085/Z/14/Z, 088158/Z/09/Z), National Natural Science Foundation of China (91843302), and National Key Research and Development Program of China (2016YFC 0900500, 0900501, 0900504, 1303904), The UK Medical Research Council (MC_UU_00017/1, MC_UU_12026/2 MC_U137686851), Cancer Research UK (C16077/A29186; C500/A16896) and British Heart Foundation (CH/1996001/9454). The chief

For peer review only - http://bmjopen.bmj.com/site/about/guidelines.xhtml

For peer review only

acknowledgment is to the participants, the project staff, and the China National Centre for Disease Control and Prevention (CDC) and its regional offices for access to death and disease registries. The Chinese National Health Insurance scheme provides electronic linkage to all hospitalised individuals.

Ethics statement

Ethics approval was obtained from the Oxford University, the China National CDC, and also from institutional research boards at the local CDCs in the 10 areas. All participants provided written informed consent.

For peer review only

For peer review only - http://bmjopen.bmj.com/site/about/guidelines.xhtml

References

1. de Martel C, Georges D, Bray F, et al. Global burden of cancer attributable to infections in 2018: a worldwide incidence analysis. *Lancet Glob Health* 2020;8(2):e180-e90. doi: 10.1016/S2214-109X(19)30488-7
2. Smieja M, Gnarpe J, Lonn E, et al. Multiple infections and subsequent cardiovascular events in the Heart Outcomes Prevention Evaluation (HOPE) study. *Circulation* 2003;107(2):251-57. doi: 10.1161/01.CIR.0000044940.65226.1F
3. Giovannoni G, Cutter GR, Lunemann J, et al. Infectious causes of multiple sclerosis. *Lancet Neurol* 2006;5(10):887-94. doi: 10.1016/s1474-4422(06)70577-4
4. Readhead B, Haure-Mirande JV, Funk CC, et al. Multiscale analysis of independent Alzheimer's cohorts finds disruption of molecular, genetic, and clinical networks by Human Herpesvirus. *Neuron* 2018;99(1):64-82.e7. doi: 10.1016/j.neuron.2018.05.023
5. Rubicz R, Leach CT, Kraig E, et al. Seroprevalence of 13 common pathogens in a rapidly growing U.S. minority population: Mexican Americans from San Antonio, TX. *BMC Res Notes* 2011;4:433-33. doi: 10.1186/1756-0500-4-433
6. Parish WL, Laumann EO, Cohen MS, et al. Population-based study of Chlamydial infection in China: a hidden epidemic. *JAMA* 2003;289(10):1265-73. doi: 10.1001/jama.289.10.1265
7. Ji J, Sun H-K, Smith JS, et al. Seroprevalence of Human Papillomavirus types 6, 11, 16 and 18 in Chinese women. *BMC Infect Dis* 2012;12(1):137. doi: 10.1186/1471-2334-12-137
8. Huai P, Li F, Li Z, et al. Seroprevalence and associated factors of HSV-2 infection among general population in Shandong Province, China. *BMC Infect Dis* 2019;19(1):382. doi: 10.1186/s12879-019-3995-2
9. Mentzer AJ, Brenner N, Allen N, et al. Identification of host-pathogen-disease relationships using a scalable Multiplex Serology platform in UK Biobank. *medRxiv* 2019:19004960. doi: 10.1101/19004960
10. Xia G-L, Liu C-B, Cao H-L, et al. Prevalence of hepatitis B and C virus infections in the general Chinese population. Results from a nationwide cross-sectional seroepidemiologic study of hepatitis A, B, C, D, and E virus infections in China, 1992. *International Hepatology Communications* 1996;5(1):62-73. doi: https://doi.org/10.1016/S0928-4346(96)82012-3
11. Liang X, Bi S, Yang W, et al. Epidemiological serosurvey of Hepatitis B in China—Declining HBV prevalence due to Hepatitis B vaccination. *Vaccine* 2009;27(47):6550-57. doi: https://doi.org/10.1016/j.vaccine.2009.08.048
12. Liu J, Zhang S, Wang Q, et al. Seroepidemiology of hepatitis B virus infection in 2 million men aged 21–49 years in rural China: a population-based, cross-sectional study. *Lancet Infect Dis* 2016;16(1):80-86. doi: https://doi.org/10.1016/S1473-3099(15)00218-2
13. Chen Z, Chen J, Collins R, et al. China Kadoorie Biobank of 0.5 million people: survey methods, baseline characteristics and long-term follow-up. *Int J Epidemiol* 2011;40(6):1652-66. doi: 10.1093/ije/dyr120
14. Waterboer T, Sehr P, Michael KM, et al. Multiplex Human Papillomavirus serology based on in situ–purified glutathione S-transferase fusion proteins. *Clinical Chemistry* 2005;51(10):1845-53. doi: 10.1373/clinchem.2005.052381
15. Michel A, Waterboer T, Kist M, et al. Helicobacter pylori multiplex serology. *Helicobacter* 2009;14(6):525-35. doi: 10.1111/j.1523-5378.2009.00723.x
16. Migchelsen SJ, Martin DL, Southisombath K, et al. Defining Seropositivity Thresholds for Use in Trachoma Elimination Studies. *PLoS Negl Trop Dis* 2017;11(1):e0005230-e30. doi: 10.1371/journal.pntd.0005230
17. Bassig BA, Willhauck-Fleckenstein M, Shu X-O, et al. Serologic markers of viral infection and risk of non-Hodgkin lymphoma: A pooled study of three prospective cohorts in China and Singapore. *Int J Cancer* 2018;143(3):570-79. doi: 10.1002/ijc.31385

18. Arbyn M, Weiderpass E, Bruni L, et al. Estimates of incidence and mortality of cervical cancer in 2018: a worldwide analysis. *Lancet Glob Health* 2020;8(2):e191-e203. doi: 10.1016/S2214-109X(19)30482-6

19. Chen C-J, Viscidi RP, Chuang C-H, et al. Seroprevalence of human papillomavirus types 16 and 18 in the general population in Taiwan: Implication for optimal age of human papillomavirus vaccination. *J Clin Virol* 2007;38(2):126-30. doi: https://doi.org/10.1016/j.jcv.2006.12.004

20. Liu F, Deng Q, Zhang C, et al. Human papillomavirus DNA positivity and seropositivity in rural Chinese men and women: a population-based cross-sectional study. *Sci Rep* 2016;6(1):26343. doi: 10.1038/srep26343

21. Ma X, Wang Q, Ong JJ, et al. Prevalence of human papillomavirus by geographical regions, sexual orientation and HIV status in China: a systematic review and meta-analysis. *Sex Transm Infect* 2018;94(6):434-42. doi: 10.1136/sextrans-2017-053412

22. Niedobitek G. The Epstein-Barr virus: a group 1 carcinogen? *Virchows Arch* 1999;435(2):79-86. doi: 10.1007/s004280050402

23. Lin H, He N, Su M, et al. Herpes simplex virus infections among rural residents in eastern China. *BMC Infect Dis* 2011;11(1):69. doi: 10.1186/1471-2334-11-69

24. Grinde B. Herpesviruses: latency and reactivation - viral strategies and host response. *J Oral Microbiol* 2013;5:10.3402/jom.v5i0.22766. doi: 10.3402/jom.v5i0.22766

25. Jansen MAE, van den Heuvel D, Bouthoorn SH, et al. Determinants of ethnic differences in Cytomegalovirus, Epstein-Barr Virus, and Herpes Simplex Virus type 1 seroprevalence in childhood. *J Pediatr* 2016;170:126-34.e6. doi: https://doi.org/10.1016/j.jpeds.2015.11.014

26. Malhotra J, Waterboer T, Pawlita M, et al. Serum biomarkers of polyomavirus infection and risk of lung cancer in never smokers. *Br J Cancer* 2016;115(9):1131-39. doi: 10.1038/bjc.2016.285

27. Zhang C, Liu F, He Z, et al. Seroprevalence of Merkel Cell Polyomavirus in the general rural population of Anyang, China. *PLoS One* 2014;9(9):e106430. doi: 10.1371/journal.pone.0106430

28. Li M, Sun Y, Yang J, et al. Time trends and other sources of variation in Helicobacter pylori infection in mainland China: A systematic review and meta-analysis. *Helicobacter* 2020;25(5):e12729. doi: https://doi.org/10.1111/hel.12729

29. Klein PD, Opekun AR, Smith EO, et al. Water source as risk factor for Helicobacter pylori infection in Peruvian children. *The Lancet* 1991;337(8756):1503-06. doi: https://doi.org/10.1016/0140-6736(91)93196-G

30. Perry S, de la Luz Sanchez M, Yang S, et al. Gastroenteritis and transmission of Helicobacter pylori infection in households. *Emerg Infect Dis* 2006;12(11):1701-8. doi: 10.3201/eid1211.060086

31. Hooi JKY, Lai WY, Ng WK, et al. Global prevalence of Helicobacter pylori infection: systematic review and meta-analysis. *Gastroenterology* 2017;153(2):420-29. doi: 10.1053/j.gastro.2017.04.022

32. Nagy P, Johansson S, Molloy-Bland M. Systematic review of time trends in the prevalence of Helicobacter pylori infection in China and the USA. *Gut Pathog* 2016;8:8-8. doi: 10.1186/s13099-016-0091-7

33. Yu S, Xu L, Jiang Z, et al. Report on the first nationwide survey of the distribution of human parasites in China, regional distribution of parasite species. *Chinese Journal of Parasitology and Parasitic Diseases* 1994;12(4):241-47.

34. Zhou P, Chen N, Zhang R-L, et al. Food-borne parasitic zoonoses in China: perspective for control. *Trends Parasitol* 2008;24(4):190-96.

35. Xiao Y, Yin J, Jiang N, et al. Seroepidemiology of human Toxoplasma gondii infection in China. *BMC Infect Dis* 2010;10(1):4.

36. Diseases COotNSotIHP. A national survey on current status of the important parasitic diseases in human population. *Chinese Journal of Parasitology and Parasitic Diseases* 2005;23(5 Suppl):332.

For peer review only

Figure legends:

Figure 1. Sero-prevalence of multiple pathogens by year of birth

Dots and grey lines represent sero-prevalence estimates and 95% CI for every single birth year. The blue line represents estimates for these sero-prevalences derived from LOESS method, and the shaded areas show the 95% CI. Due to few participants that were born before 1932 or after 1971, those who were born between 1927-1931, or 1971-1976 were combined, separately.

Figure 2. Sero-prevalence of multiple pathogens across 10 study areas

The grey bar represents urban participants and white bar represents rural participants. The number of participants in each region was 198 (Qingdao), 295 (Harbin), 135 (Haikou), 194 (Suzhou), 174 (Liuzhou), 162 (Sichuan), 178 (Gansu), 208 (Henan), 245 (Zhejiang) and 197 (Hunan).

Figure 3. Distribution of number of infections for (a, b) any of 19 pathogens; and (c, d) oncogenic pathogens, overall and by year of birth

Numbers with different infections ([a] and [c]), and sero-prevalence for >10 pathogens (b), or >2 pathogens (d) by birth years were presented. Pathogens of HBV, HCV, HIV, HTLV, *H. pylori*, HPV (16, 18) and EBV are classified as Group I human carcinogen by the IARC. The dashed lines represent the mean numbers of seropositive pathogens among the study participants, which were 9.4 (SD 1.7) for any of 19 pathogens, and 2.1 (SD 0.8) for any of 8 oncogenic pathogens. Proportion of individuals with >10 infectious pathogens, or with >2 oncogenic pathogens was presented by birth cohort. Due to few participants that were born before 1932 or after 1971, those who were born between 1927-1931, or 1971-1976 were combined, separately. The blue line represents estimate for these sero-prevalence derived from LOESS method, and the shaded areas show the 95% CI.

For peer review only

Table 1. Baseline characteristics of the study subcohort and overall CKB cohort

Characteristics	Subcohort (n=1,986)	CKB (n=453,157)
**Demographic and lifestyle factors**		
Age, years	51.8 (10.8)	51.7 (10.6)
Birth year, %		
≤1939	12.0	11.0
1940-1949	20.0	20.6
1950-1959	33.2	31.9
1960-1969	29.2	30.8
≥1970	5.6	5.7
Female, %	61.7	60.2
Urban residents, %	50.3	45.4
Education ≥high school, %	21.9	21.5
Household income (>20000 yuan/year), %	42.4	43.2
Living alone, %	3.6	2.8
Ever regular smoker in men, %	73.9	74.0
Ever regular smoker in women, %	3.6	3.1
Ever regular drinker in men, %	38.4	36.9
Ever regular drinker in women, %	3.2	2.4
Physical activity, MET h/day	21.1 (14.2)	21.3 (13.9)
**Medical history and health status, %**		
Poor self-rated health	10.2	10.0
HBsAg positive [1]	3.2	3.1
Diabetes [2]	5.7	5.6
History of peptic ulcer	3.9	3.9
History of cirrhosis/chronic hepatitis	1.3	1.2
Family history of cancer	14.8	14.2
**Anthropometry, mean (SD)**		
BMI, kg/m^2	23.8 (3.5)	23.6 (3.4)
Waist circumference, cm	80.1 (9.9)	80.1 (9.7)
SBP, mmHg	130 (21)	130 (21)
Random plasma glucose, mmol/L	5.8 (2.3)	6.0 (2.3)

The results are presented as means (SD) or percentages. Abbreviations: MET-h, metabolic equivalent of task-hours; SBP, systolic blood pressure; HBsAg, Hepatitis B surface antigen;

[1] Hepatitis B surface antigen (HBsAg) was tested using an on-site rapid test strip at baseline

[2] Includes those with a (self-reported) previous medical diagnosis of diabetes and those detected by blood glucose tests at baseline

For peer review only

Table 2. Sero-prevalence estimates (95% CI) for infectious pathogens in men and women

Pathogens	Men (n=761)	Women (n=1225)	All (n=1986)
**Hepatitis viruses**			
HBV	46.1 (42.5-49.7)	42.4 (39.6-45.2)	43.8 (41.6-46.0)
HCV	0.5 (0.1-1.3)	0.8 (0.4-1.5)	0.7 (0.4-1.2)
**Human papillomaviruses (HPV)**			
HPV-16	**4.1 (2.8-5.7)**	**7.9 (6.5-9.6)**	6.4 (5.4-7.6)
HPV-18	2.8 (1.7-4.2)	3.8 (2.8-5.0)	3.4 (2.6-4.3)
**Human herpesviruses (HHV)**			
HHV-1	97.2 (95.8-98.3)	98.4 (97.6-99.1)	98.0 (97.3-98.6)
HHV-2	7.1 (5.4-9.2)	10.0 (8.3-11.8)	8.9 (7.6-10.2)
VZV	92.9 (90.8-94.6)	90.7 (88.9-92.3)	91.5 (90.2-92.7)
EBV	99.9 (99.3-100.0)	99.8 (99.4-100.0)	99.8 (99.6-100.0)
CMV	95.9 (94.3-97.2)	98.1 (97.2-98.8)	97.3 (96.5-98.0)
HHV-6	**52.3 (48.7-55.9)**	**68.0 (65.3-70.6)**	62.0 (59.8-64.1)
HHV-7	**79.5 (76.5-82.3)**	**89.3 (87.4-91.0)**	85.5 (83.9-87.1)
**Human polyomaviruses (HPyV)**			
BKV	90.8 (88.5-92.8)	91.3 (89.6-92.9)	91.1 (89.8-92.4)
JCV	68.5 (65.0-71.8)	63.8 (61.1-66.5)	65.6 (63.5-67.7)
MCV	64.7 (61.1-68.1)	65.1 (62.3-67.7)	64.9 (62.8-67.0)
**Other viruses**			
HIV	0	0.1 (0.0-0.5)	0.1 (0.0-0.3)
HTLV	0.1 (0.0-0.7)	0.1 (0.0-0.5)	0.1 (0.0-0.4)
**Bacteria/Parasite**			
H. pylori	54.3 (50.7-57.9)	53.0 (50.1-55.8)	53.5 (51.3-55.7)
C. trachomatis	45.7 (42.1-49.3)	48.8 (46.0-51.7)	47.6 (45.4-49.9)
T. gondii	12.4 (10.1-14.9)	15.3 (13.4-17.5)	14.2 (12.7-15.8)

Bold values denote statistical significance at the p < 0.05 level.

For peer review only

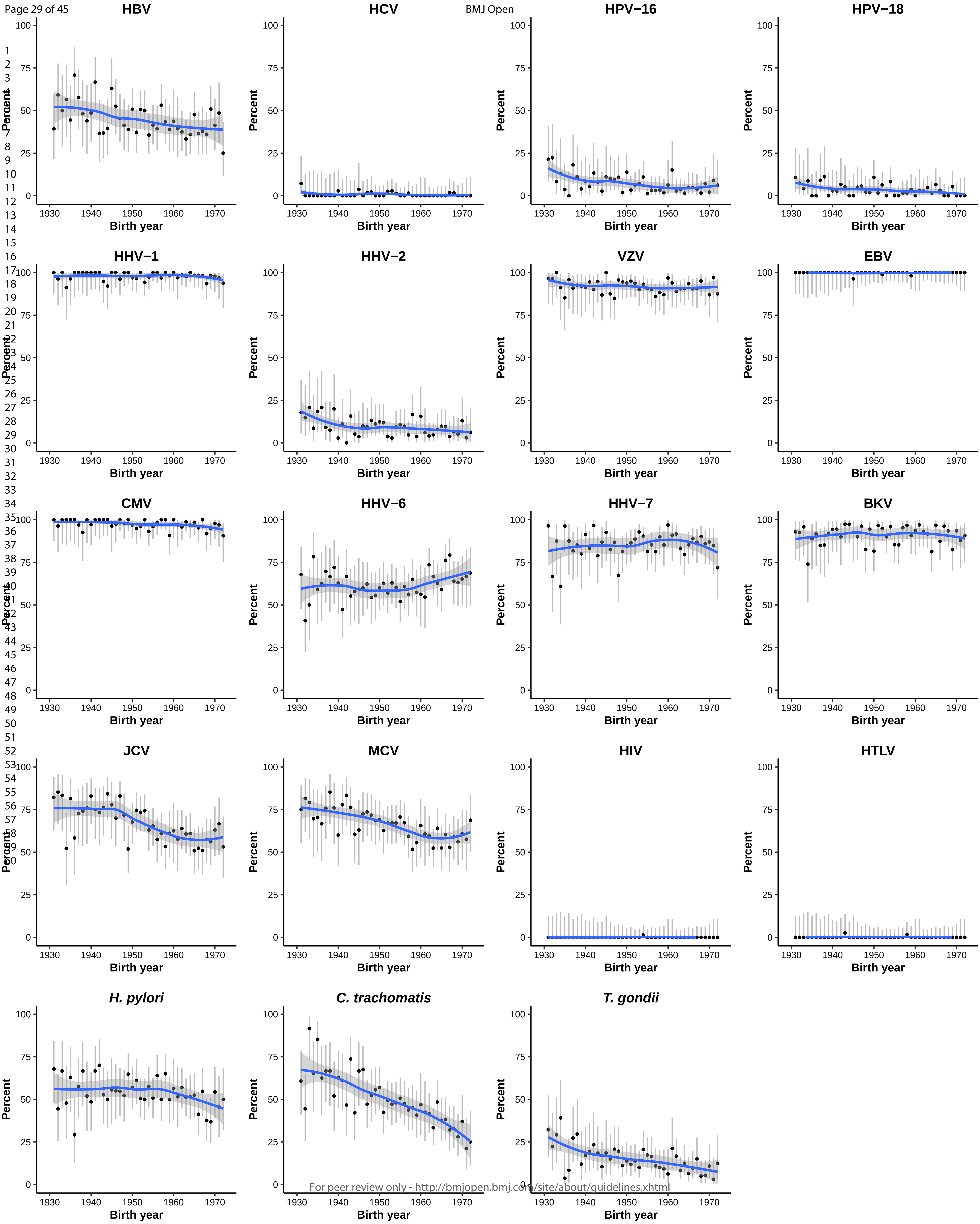
HBV
HCV
HPV−16
HPV−18
HHV−1
HHV−2
VZV
EBV
CMV
HHV−6
HHV−7
BKV
JCV
MCV
HIV
HTLV
H. pylori
C. trachomatis
T. gondii
Percent
Birth year

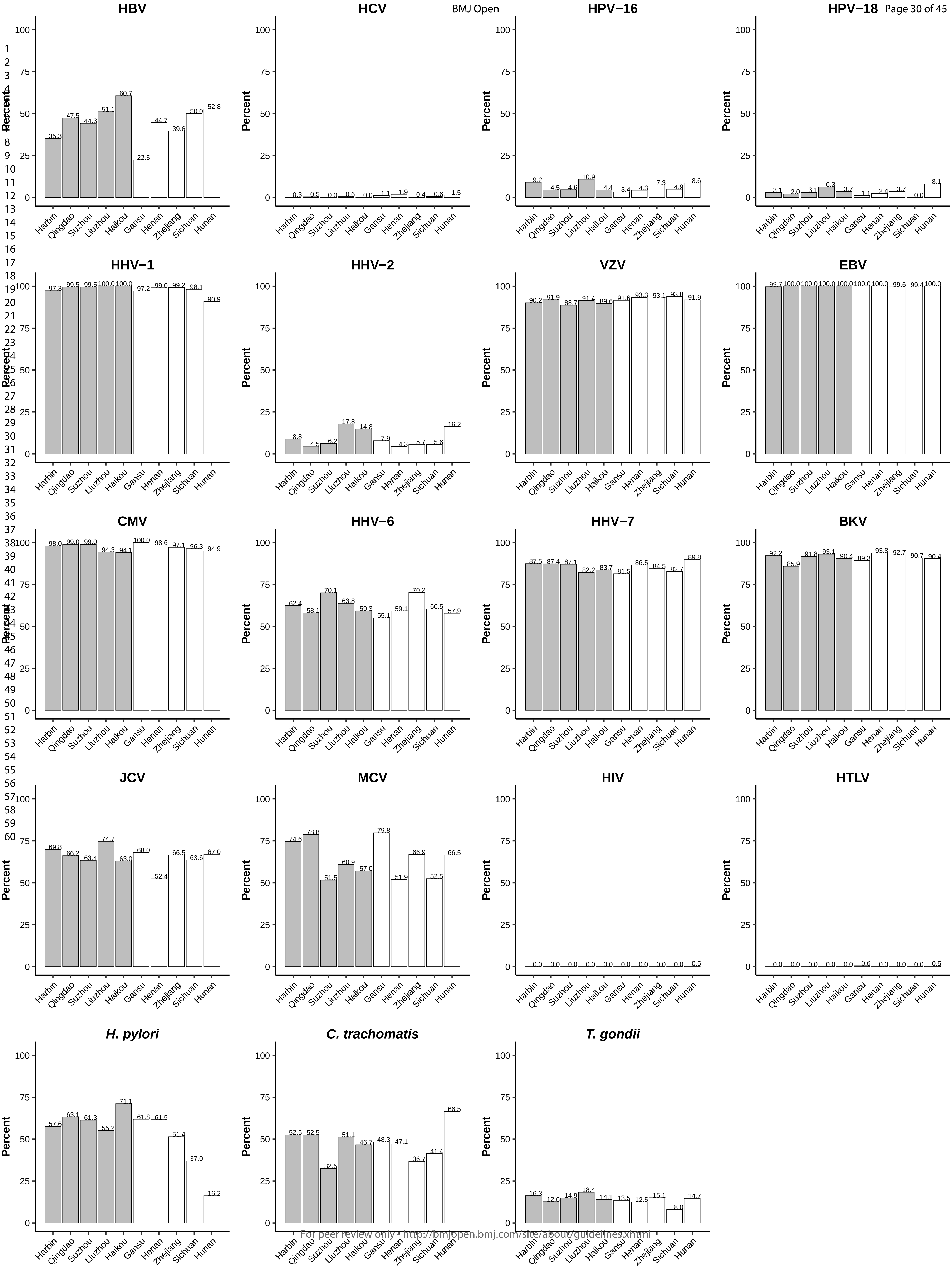
HBV
Percent
35.3 47.5 44.3 51.1 60.7 22.5 44.7 39.6 50.0 52.8
HCV
0.3 0.5 0.0 0.6 0.0 1.1 1.9 0.4 0.6 1.5
HPV−16
9.2 4.5 4.6 10.9 4.4 3.4 4.3 7.3 4.9 8.6
HPV−18
3.1 2.0 3.1 6.3 3.7 1.1 2.4 3.7 0.0 8.1
HHV−1
97.3 99.5 99.5 100.0 100.0 97.2 99.0 99.2 98.1 90.9
HHV−2
8.8 4.5 6.2 17.8 14.8 7.9 4.3 5.7 5.6 16.2
VZV
90.2 91.9 88.7 91.4 89.6 91.6 93.3 93.1 93.8 91.9
EBV
99.7 100.0 100.0 100.0 100.0 100.0 100.0 99.6 99.4 100.0
CMV
98.0 99.0 99.0 94.3 94.1 100.0 98.6 97.1 96.3 94.9
HHV−6
62.4 58.1 70.1 63.8 59.3 55.1 59.1 70.2 60.5 57.9
HHV−7
87.5 87.4 87.1 82.2 83.7 81.5 86.5 84.5 82.7 89.8
BKV
92.2 85.9 91.8 93.1 90.4 89.3 93.8 92.7 90.7 90.4
JCV
69.8 66.2 63.4 74.7 63.0 68.0 52.4 66.5 63.6 67.0
MCV
74.6 78.8 51.5 60.9 57.0 79.8 51.9 66.9 52.5 66.5
HIV
0.0 0.0 0.0 0.0 0.0 0.0 0.0 0.0 0.0 0.5
HTLV
0.0 0.0 0.0 0.0 0.0 0.6 0.0 0.0 0.0 0.5
H. pylori
57.6 63.1 61.3 55.2 71.1 61.8 61.5 51.4 37.0 16.2
C. trachomatis
52.5 52.5 32.5 51.1 46.7 48.3 47.1 36.7 41.4 66.5
T. gondii
16.3 12.6 14.9 18.4 14.1 13.5 12.5 15.1 8.0 14.7
Harbin Qingdao Suzhou Liuzhou Haikou Gansu Henan Zhejiang Sichuan Hunan
0 25 50 75 100

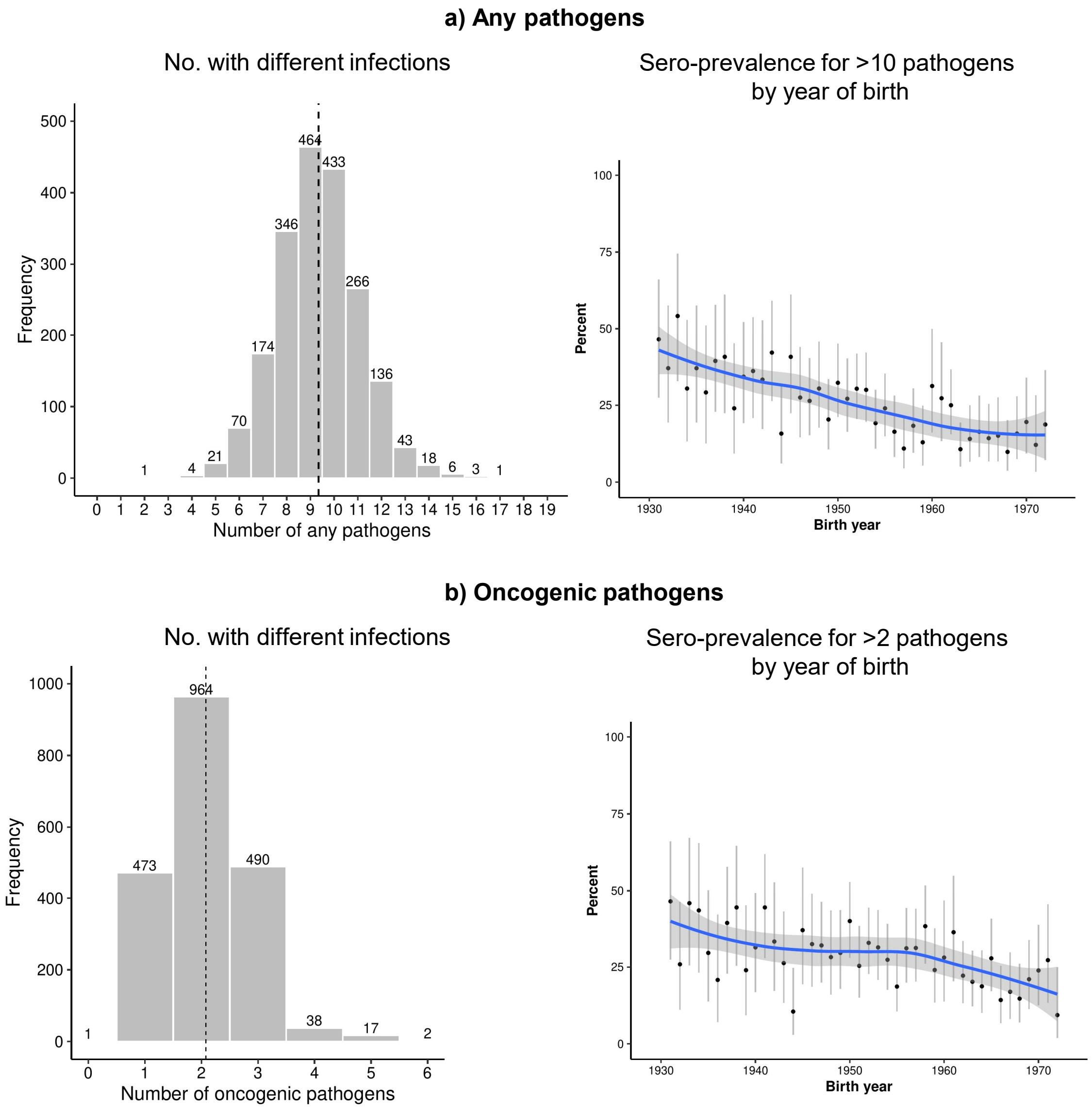
a) Any pathogens
No. with different infections
Frequency
Number of any pathogens
1
4
21
70
174
346
464
433
266
136
43
18
6
3
1
Sero-prevalence for >10 pathogens by year of birth
Percent
Birth year
b) Oncogenic pathogens
No. with different infections
Frequency
Number of oncogenic pathogens
1
473
964
490
38
17
2
Sero-prevalence for >2 pathogens by year of birth
Percent
Birth year

Sero-prevalence of 19 infectious pathogens and associated factors among middle-aged and elderly Chinese adults: a cross-sectional study

Supplementary Material

For peer review only

For peer review only - http://bmjopen.bmj.com/site/about/guidelines.xhtml

eAppendix

Study population

The study design was a prospective study. Participants who were aged 30 -79 years at enrolment, were selected for the China Kadoorie Biobank study of the causes of major chronic diseases that account for most deaths in the Chinese population. Overall, 515 681 people attended the baseline survey between June 2004 and July 2008, of whom 261 (0.05%) withdrew before completion, 2208 (0.4%) were found subsequently to have inadvertently attended the survey twice at different time points and 1 had major data errors. The estimated population response rate was ~30% (26–38% in the five rural areas and 16–50% in the five urban areas). All individuals who completed a questionnaire and provided consent for participants in the study were included in the present analyses. No participants with a prior history of cardiovascular disease or cancer were excluded.

Follow-up of CKB participants was through linkage to established mortality and morbidity registries, and to the nationwide health insurance system which records all hospitalised episodes. Ethics approval was obtained from the Oxford University, the China National CDC, and also from institutional research boards at the local CDCs in the 10 areas. All participants provided written informed consent.

While the China Kadoorie Biobank is not representative of the Chinese population, it collected data from a large number of participants from 5 rural and 5 urban areas that reflected a wide range of exposures and disease outcomes. The study participants did not receive any payment for their participation in the study.

For peer review only - http://bmjopen.bmj.com/site/about/guidelines.xhtml

Repeat measurements

For 100 (5%) of these participants, plasma samples collected at the two subsequent resurveys were also assayed to assess the consistency of sero-status (sero-conversion and sero-reversion rates) of measurements over time.

The agreement between multiple measurements of same individuals at three occasions (baseline, the 1st and the 2nd resurvey) of quantitative MFI antibody levels was evaluated using Intraclass Correlation Coefficients (ICCs).

For peer review only

For peer review only - http://bmjopen.bmj.com/site/about/guidelines.xhtml

Table S1. Characteristics of pathogens and pathogen-specific antigens included in the multiplex serology and suggested cut-offs to determine seropositivity

Pathogen	Transmissions [1]	Antigen	Full name, function	Criteria for seropositivity	Cut-off (MFI)*
**Hepatitis viruses**					
Hepatitis B virus (HBV)	Perinatal, blood, semen	HBVAc	core antigen	2 positives out of 2	1000
		HBVAe	soluble nucleocapsid associated antigen		1000
Hepatitis C virus (HCV)	blood, semen	1aCore	structual antigen	2 positives out of 2	**300**
		1aNS3	protease and RNA helicase activity		150
**Human papillomaviruses (HPV)**					
HPV-16	sexual contact	L1	major capsid protein		80
		E6	oncoproteins		**100**
		E7	oncoproteins		50
HPV-18	sexual contact	L1	major capsid protein		170
**Human herpesviruses (HHV)**					
HHV-1 (HSV-1/)	sores, saliva, and surface in or around mouth	gG	membrane glycoprotein		54
HHV-2 (HSV-2)	genital surfaces, skin, sores or fluids	mgG unique	membrane glycoprotein		180
Varicella zoster virus (VZV/HHV-3)	blisters, saliva or mucus	gE/gI	envelope glycoproteins		100
Epstein-Barr virus (EBV/HHV-4)	saliva, blood or semen	ZEBRA	replication activator	≥2 positives out of 4	74
		EA-D	replication (polymerase accessory subunit)		110
		VCAp18	capsid protein		2526
		EBNA (peptid)	replication, latent viral infection		411
Human cytomegalovirus (CMV/HHV-5)	saliva, urine, blood, tears, semen or breast milk	pp150 Nter	tegument protein	≥2 positives out of 3	655
		pp52	DNA binding protein		1101
		pp28	capsid protein		200
HHV-6	saliva	IE1A	potential transactivator	≥1 positive out of 2	300
		IE1B	potential transactivator		300
HHV-7	saliva	U14	potential tegument protein		**100**
**Human polyomaviruses (HPyV)**					
HPyV BK (BKV)	respiratory fluids or urine	VP1	major capsid protein		250
HPyV JC (JCV)	respiratory fluids or urine	VP1	major capsid protein		250
HPyV MCV (MCV)	respiratory fluids	VP1	major capsid protein		250
**Other viruses**					
Human immunodeficiency virus (HIV)	blood, semen, vaginal and rectal secretions, or breast milk	gag	structual antigen	2 positives out of 2	1000
		env	structual antigen		150
Human T-lymphotropic virus (HTLV)	breast milk, blood,	gag	structual antigen	2 positives out of 2	700
		nv	structual antigen		50
**Bacteria/Parasite**					
Helicobacter pylori (H. pylori)	saliva (oral-oral, gastro-oral and fecal-oral)	HyuA	hydantoin utilization protein A	≥4 positives out of 12	**280**
		GroEL	chaperonin GroEL		500
		UreA	urease alpha subunit		**300**
		CagA-N/C	cytotoxin-associated antigen A, pathogenesis		1500
		Catalase			**300**
		HP1564	hypothetical protein		150
		VacA-N/C	vacuolating cytotoxin, pathogenesis		385
		NapA	neutrophil-activating protein		120
		HP0305	hypothetical protein		**130**
		HpaA	neuraminyllactose-binding hemagglutinin homolog		200
		Cad	cinnamyl alcohol dehydrogenase		100
		HcpC	conserved hypothetical secreted protein		150
Chlamydia trachomatis (C. trachomatis)	sexual contact	pGP3	virulence factor		**300**
Toxoplasma gondii (T. gondii)	undercooked meat, blood	sag1	surface protein	≥1 positive out of 2	50
		p22	surface protein		50

MFI: Median Fluorescence Intensities

[1] The route of transmission of some pathogens is not completely understood, and the major route is listed

For peer review only

Table S2. Adjusted odds ratios of sero-positivity for selected pathogens by baseline characteristics

	HBV	HPV-16	HHV-2	VZV	HHV-6	HHV-7	BKV	JCV	MCV	*H. pylori*	*C. trachomatis*	*T. gondii*
Sex, female vs male	0.86 (0.72, 1.03)	**2.07 (1.36, 3.14)**	1.45 (1.04, 2.03)	0.76 (0.54, 1.06)	**1.92 (1.59, 2.31)**	**2.15 (1.67, 2.76)**	1.08 (0.78, 1.48)	0.82 (0.68, 1.00)	1.04 (0.85, 1.25)	0.93 (0.78, 1.12)	1.18 (0.98, 1.42)	1.31 (1.00, 1.71)
Age (reference group: ≤39 years)												
40-49	1.01 (0.77, 1.34)	0.73 (0.38, 1.41)	1.12 (0.66, 1.90)	0.84 (0.52, 1.37)	0.78 (0.58, 1.05)	0.99 (0.66, 1.48)	1.25 (0.78, 2.01)	1.03 (0.78, 1.36)	1.02 (0.77, 1.35)	1.21 (0.91, 1.59)	**1.43 (1.08, 1.91)**	1.33 (0.84, 2.11)
50-59	1.15 (0.87, 1.51)	1.51 (0.84, 2.73)	1.34 (0.80, 2.24)	1.08 (0.65, 1.78)	0.63 (0.47, 0.85)	0.92 (0.62, 1.37)	1.08 (0.68, 1.72)	**1.53 (1.15, 2.03)**	**1.54 (1.16, 2.04)**	**1.37 (1.04, 1.81)**	**2.18 (1.64, 2.90)**	**1.85 (1.18, 2.90)**
≥60	**1.50 (1.12, 2.00)**	**2.06 (1.14, 3.73)**	1.67 (0.99, 2.81)	1.09 (0.64, 1.85)	0.70 (0.52, 0.96)	0.94 (0.62, 1.42)	1.17 (0.71, 1.91)	**2.30 (1.69, 3.13)**	**2.01 (1.48, 2.72)**	1.29 (0.97, 1.73)	**3.51 (2.59, 4.74)**	**2.54 (1.62, 3.99)**
Region, urban vs rural	1.15 (0.96, 1.37)	1.12 (0.78, 1.62)	1.22 (0.89, 1.67)	0.74 (0.53, 1.02)	1.07 (0.89, 1.29)	1.04 (0.81, 1.34)	0.90 (0.66, 1.23)	1.17 (0.97, 1.41)	1.07 (0.89, 1.29)	**1.80 (1.50, 2.15)**	0.92 (0.77, 1.10)	1.14 (0.88, 1.47)
Education, ≥high school vs <high school	0.93 (0.72, 1.19)	**1.63 (1.01, 2.65)**	1.23 (0.81, 1.88)	0.87 (0.57, 1.32)	0.83 (0.64, 1.07)	1.09 (0.77, 1.54)	0.88 (0.58, 1.35)	0.9 (0.70, 1.17)	0.95 (0.73, 1.23)	1.09 (0.85, 1.41)	**0.70 (0.54, 0.90)**	1.16 (0.82, 1.65)
Household income, ≥20k vs <20k	1.03 (0.83, 1.27)	0.84 (0.56, 1.27)	0.89 (0.62, 1.28)	1.26 (0.87, 1.82)	0.89 (0.72, 1.11)	1.06 (0.78, 1.43)	0.95 (0.66, 1.37)	1.03 (0.82, 1.29)	0.98 (0.78, 1.23)	0.89 (0.71, 1.11)	**0.80 (0.64, 0.99)**	1.21 (0.90, 1.62)
Physical activity (high vs low)	1.27 (1.03, 1.56)	**0.50 (0.32, 0.78)**	0.82 (0.57, 1.19)	**0.65 (0.45, 0.94)**	0.91 (0.73, 1.12)	1.44 (1.08, 1.93)	0.88 (0.62, 1.26)	1.06 (0.86, 1.32)	0.99 (0.80, 1.23)	0.98 (0.79, 1.21)	0.88 (0.72, 1.09)	0.75 (0.56, 1.01)
Ever regular smoker (yes vs no)	1.15 (0.86, 1.55)	0.59 (0.31, 1.13)	0.88 (0.51, 1.51)	1.09 (0.63, 1.89)	1.00 (0.74, 1.34)	1.15 (0.79, 1.68)	0.93 (0.56, 1.55)	1.06 (0.77, 1.46)	**0.71 (0.51, 0.97)**	1.03 (0.76, 1.40)	0.92 (0.68, 1.25)	0.77 (0.50, 1.18)
Ever regular drinker (yes vs no)	1.08 (0.81, 1.42)	0.55 (0.27, 1.14)	0.84 (0.48, 1.47)	1.29 (0.75, 2.22)	1.01 (0.76, 1.33)	1.03 (0.72, 1.48)	1.10 (0.68, 1.78)	0.96 (0.72, 1.29)	1.06 (0.79, 1.43)	0.91 (0.69, 1.21)	1.01 (0.76, 1.34)	0.86 (0.56, 1.31)
Self-rated poor health (yes vs no)	0.90 (0.66, 1.23)	1.31 (0.75, 2.27)	1.11 (0.67, 1.82)	1.05 (0.61, 1.82)	0.88 (0.65, 1.19)	**0.60 (0.41, 0.88)**	0.97 (0.57, 1.63)	0.96 (0.69, 1.31)	0.96 (0.70, 1.32)	0.83 (0.61, 1.13)	0.98 (0.72, 1.34)	1.27 (0.85, 1.89)
Hepatitis B test (positive vs negative)	**11.51 (5.16, 25.7)**	0.57 (0.13, 2.40)	0.73 (0.26, 2.09)	0.90 (0.38, 2.13)	0.78 (0.46, 1.31)	1.97 (0.78, 5.02)	1.10 (0.43, 2.80)	1.11 (0.65, 1.90)	1.44 (0.83, 2.50)	1.14 (0.66, 1.97)	1.34 (0.79, 2.27)	0.77 (0.32, 1.82)
Diabetes (yes vs no)	1.20 (0.81, 1.77)	**2.69 (1.52, 4.77)**	1.31 (0.70, 2.45)	0.86 (0.44, 1.67)	0.86 (0.58, 1.28)	0.75 (0.45, 1.26)	1.25 (0.61, 2.55)	**0.52 (0.35, 0.78)**	1.22 (0.78, 1.91)	1.06 (0.71, 1.59)	**1.55 (1.03, 2.34)**	1.28 (0.78, 2.09)
History of peptic ulcer (yes vs no)	1.09 (0.68, 1.74)	0.19 (0.03, 1.38)	0.54 (0.19, 1.51)	1.23 (0.49, 3.11)	0.82 (0.51, 1.32)	1.87 (0.88, 3.98)	0.91 (0.41, 2.04)	0.89 (0.54, 1.47)	0.78 (0.48, 1.27)	**0.52 (0.32, 0.85)**	1.09 (0.67, 1.76)	0.80 (0.39, 1.64)
History of cirrhosis/chronic hepatitis (yes vs no)	**7.51 (2.55, 22.13)**	2.20 (0.62, 7.73)	0.46 (0.06, 3.49)	0.66 (0.19, 2.24)	0.86 (0.39, 1.92)	1.48 (0.44, 5.03)	2.21 (0.30, 16.47)	1.56 (0.64, 3.80)	0.88 (0.39, 1.97)	0.79 (0.35, 1.75)	0.57 (0.24, 1.37)	0.55 (0.13, 2.35)
Family history of cancer	1.13 (0.87, 1.47)	1.05 (0.62, 1.80)	0.92 (0.56, 1.51)	0.86 (0.56, 1.33)	0.94 (0.72, 1.23)	0.85 (0.59, 1.21)	1.38 (0.84, 2.26)	1.19 (0.91, 1.57)	0.99 (0.76, 1.31)	1.18 (0.91, 1.54)	0.82 (0.63, 1.07)	0.96 (0.66, 1.39)
BMI≥25 kg/m^2 (yes vs no)	1.09 (0.89, 1.32)	**1.51 (1.03, 2.22)**	1.05 (0.75, 1.49)	1.03 (0.73, 1.46)	0.93 (0.76, 1.14)	0.80 (0.61, 1.05)	1.06 (0.75, 1.48)	1.00 (0.82, 1.23)	1.12 (0.91, 1.37)	0.93 (0.76, 1.14)	1.01 (0.82, 1.23)	1.02 (0.77, 1.34)
Central obesity (yes vs no)1	1.03 (0.83, 1.27)	1.29 (0.86, 1.93)	1.00 (0.70, 1.45)	1.01 (0.70, 1.45)	0.98 (0.79, 1.22)	0.93 (0.68, 1.27)	1.02 (0.71, 1.47)	**0.78 (0.62, 0.97)**	1.24 (0.99, 1.56)	0.98 (0.79, 1.22)	0.94 (0.75, 1.16)	0.95 (0.71, 1.28)

Adjust for age, sex and region (where appropriate). Bold values denote statistical significance at the $p < 0.05$ level.
1 Central obesity is defined as a waist circumference greater or equal to 94 cm in men and 80 cm in women.

For peer review only

For peer review only - http://bmjopen.bmj.com/site/about/guidelines.xhtml

Table S3. Sero-conversion and sero-reversion rates estimated from 100 individuals with samples available at baseline, 1st resurvey and 2nd resurvey

Pathogen	Baseline - 1st resurvey		Baseline - 2nd resurvey		1st resurvey - 2nd resurvey	
	Sero-conversion (%)	Sero-reversion (%)	Sero-conversion (%)	Sero-reversion (%)	Sero-conversion (%)	Sero-reversion (%)
**Hepatitis viruses**						
HBV	2 (2.1)	2 (2.1)	2 (2.0)	2 (2.0)	4 (4.1)	4 (4.1)
HCV	0 (0)	1 (1.0)	0 (0)	1 (1.0)	0 (0)	0 (0)
**Human papillomaviruses (HPV)**						
HPV-16	1 (1.0)	4 (4.1)	1 (1.0)	4 (4.1)	3 (3.1)	3 (3.1)
HPV-18	0 (0)	2 (2.1)	1 (1.0)	1 (1.0)	1 (1.0)	1 (1.0)
**Human herpesviruses (HHV)**						
HHV-1	0 (0)	0 (0)	0 (0)	0 (0)	0 (0)	0 (0)
HHV-2	3 (3.1)	0 (0)	4 (4.1)	0 (0)	1 (1.0)	0 (0)
VZV	5 (5.2)	4 (4.1)	3 (3.1)	2 (2.0)	3 (3.1)	3 (3.1)
EBV	0 (0)	0 (0)	0 (0)	0 (0)	0 (0)	0 (0)
CMV	1 (1.0)	0 (0)	1 (1.0)	0 (0)	0 (0)	0 (0)
HHV-6	11 (11.3)	11 (11.3)	8 (8.2)	12 (12.2)	8 (8.2)	11 (11.2)
HHV-7	7 (7.2)	10 (10.3)	8 (8.2)	5 (5.1)	11 (11.2)	4 (4.1)
**Human polyomaviruses (HPyV)**						
BKV	2 (2.1)	7 (7.2)	4 (4.1)	4 (4.1)	8 (8.2)	2 (2.0)
JCV	2 (2.1)	3 (3.1)	2 (2.0)	3 (3.1)	3 (3.1)	3 (3.1)
MCV	2 (2.1)	5 (5.2)	3 (3.1)	6 (6.1)	2 (2.0)	3 (3.1)
**Other viruses**						
HIV	0 (0)	0 (0)	0 (0)	0 (0)	0 (0)	0 (0)
HTLV	0 (0)	0 (0)	0 (0)	0 (0)	0 (0)	0 (0)
**Bacteria/Parasite**						
H. pylori	2 (2.1)	4 (4.1)	3 (3.1)	12 (12.2)	3 (3.1)	10 (10.2)
C. trachomatis	3 (3.1)	2 (2.1)	4 (4.1)	1 (1.0)	2 (2.0)	0 (0)
T. gondii	4 (4.1)	8 (8.2)	3 (3.1)	6 (6.1)	7 (7.1)	6 (6.1)

Sero-conversion referred to the process of having "become pathogen positive", and sero-reversion is the opposite of seroconversion

For peer review only

Table S4. Age-standardized sero-prevalence (%) for infectious pathogens in CKB and UKB

Pathogens	CKB	UKB
**Hepatitis virus**		
HBV[1]	41.4	2.6
HCV[1]	0.6	0.3
**Human Papillomavirus (HPV)**		
HPV-16[1]	5.8	4.9
HPV-18[1]	3.2	3.0
**Human Herpesvirus (HHV)**		
HHV-1	93.4	68.6
HHV-2	8.3	16.8
VZV	87.1	92.2
EBV[1]	95.1	94.8
CMV	92.7	55.5
HHV-6	59.7	90.8
HHV-7	81.6	95.0
**Human Polyomavirus (HPyV)**		
BKV	86.9	95.9
JCV	61.5	62.4
MCV	60.9	67.3
**Other viruses**		
HIV[1]	0.04	0.2
HTLV[1]	0.1	1.4
**Bacteria/Parasite**		
H. pylori[1]	50.5	34.1
C. trachomatis	44.1	23.1
T. gondii	13.1	26.7
**Number of any pathogen +**	**9.3 (1.7)**	**8.4 (1.9)**
**Number of oncogenic pathogens +**	**2.1 (0.8)**	**1.4 (0.7)**

Sero-prevalence for each infectious pathogen in CKB and UKB was directly standardized to WHO World Standard Population (available from https://seer.cancer.gov/stdpopulations/world.who.html)
[1] Oncogenic pathogen

For peer review only
For peer review only - http://bmjopen.bmj.com/site/about/guidelines.xhtml

Figure S1. Locations of the 10 survey sites and number recruited.

Open circles indicate rural areas and solid circles indicate urban areas. Number recruited at baseline in each area is shown in brackets.

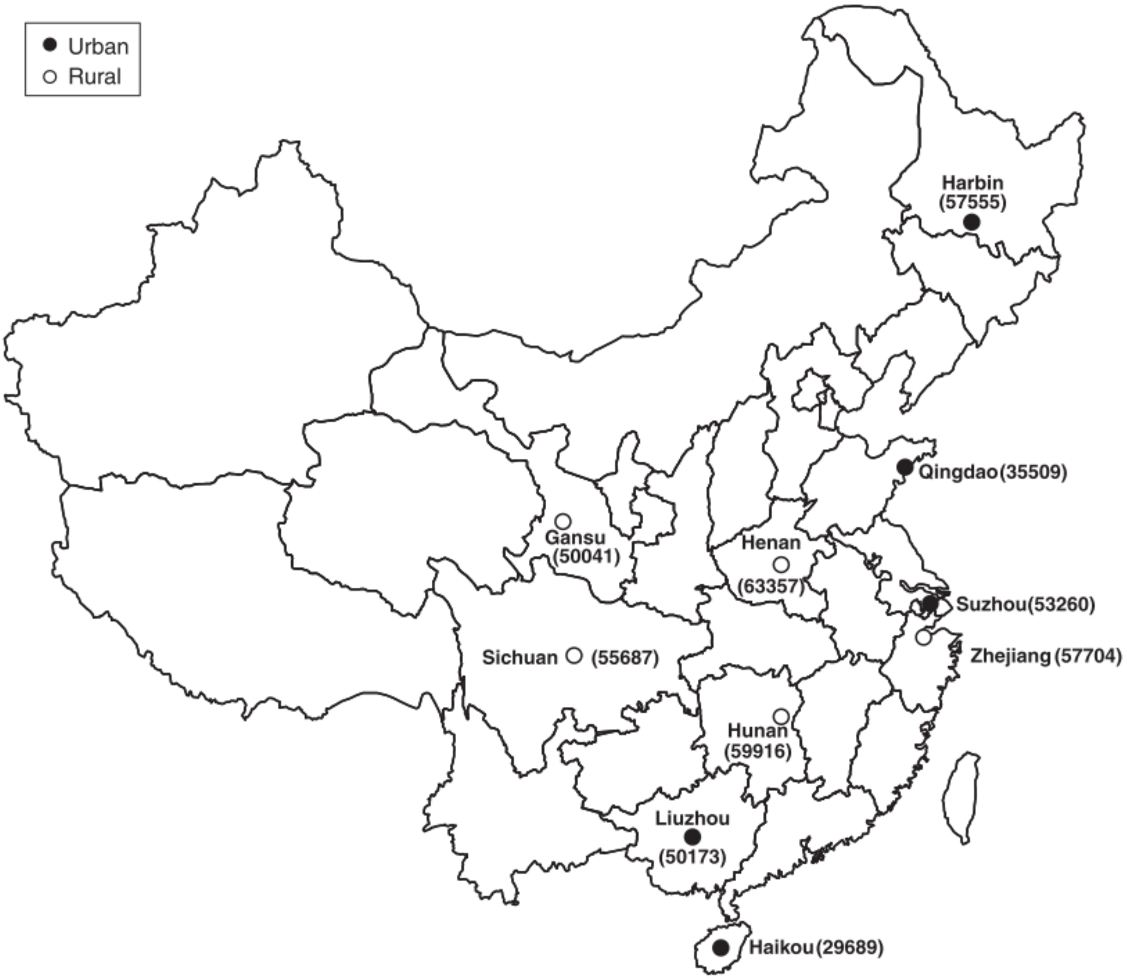

only

For peer review only - http://bmjopen.bmj.com/site/about/guidelines.xhtml

Figure S2. Distribution of median fluorescence intensity of 43 antigens

For each antigen, orange and blue represent sero-status of positive and negative, respectively.

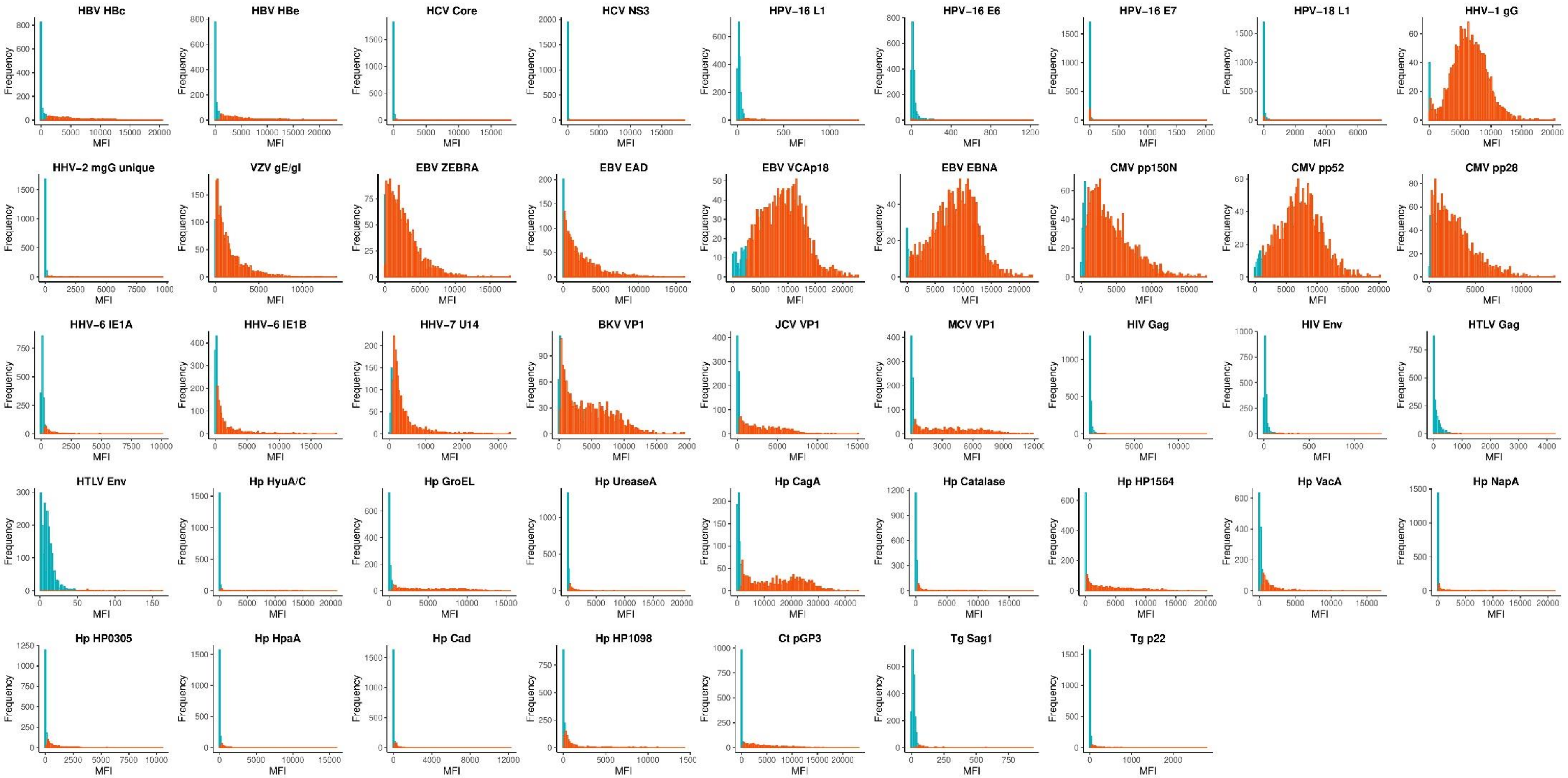

Figure S3. Correlation matrix of pathogens

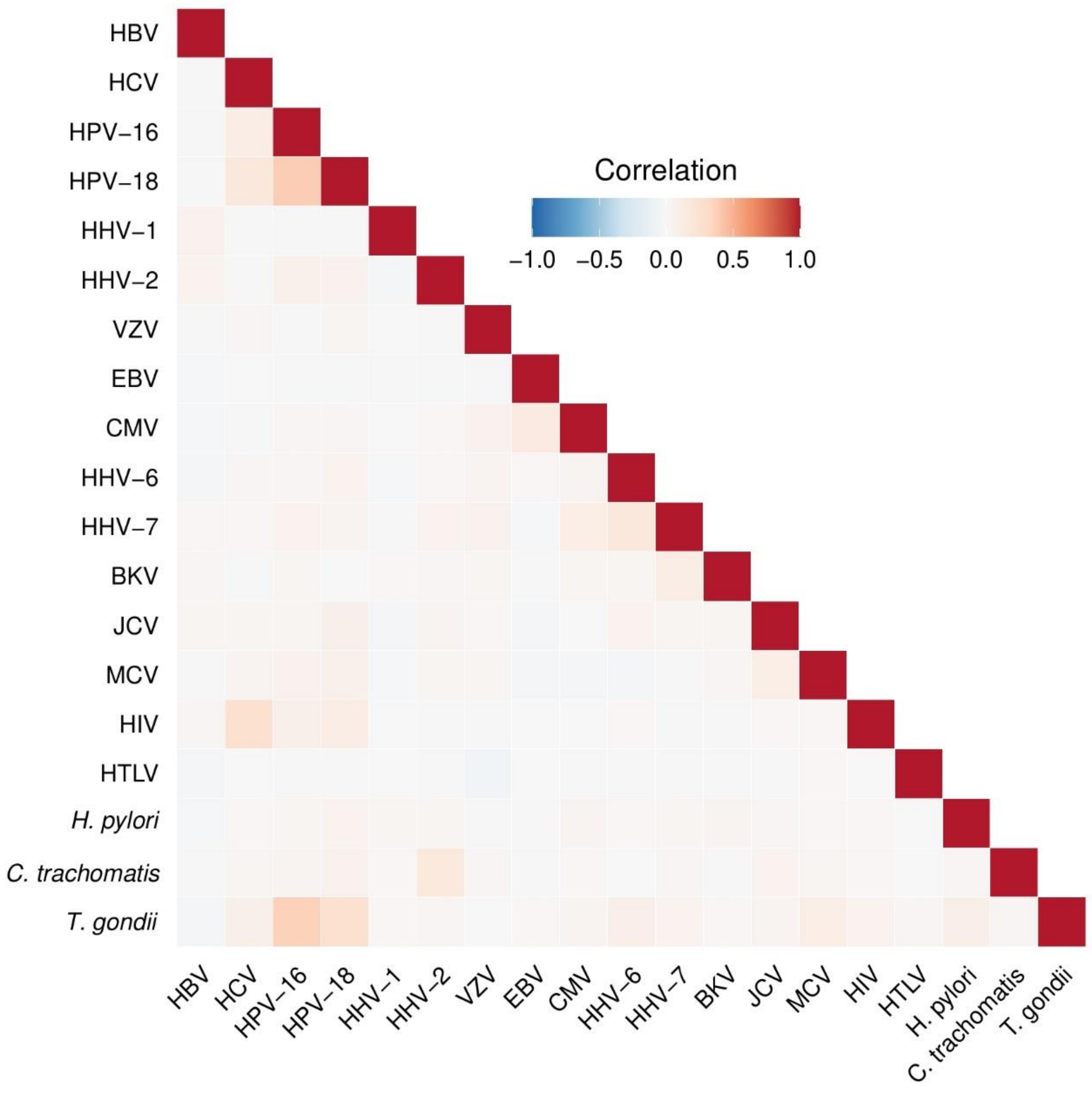

Figure S4. Correlation matrix of 43 antigens

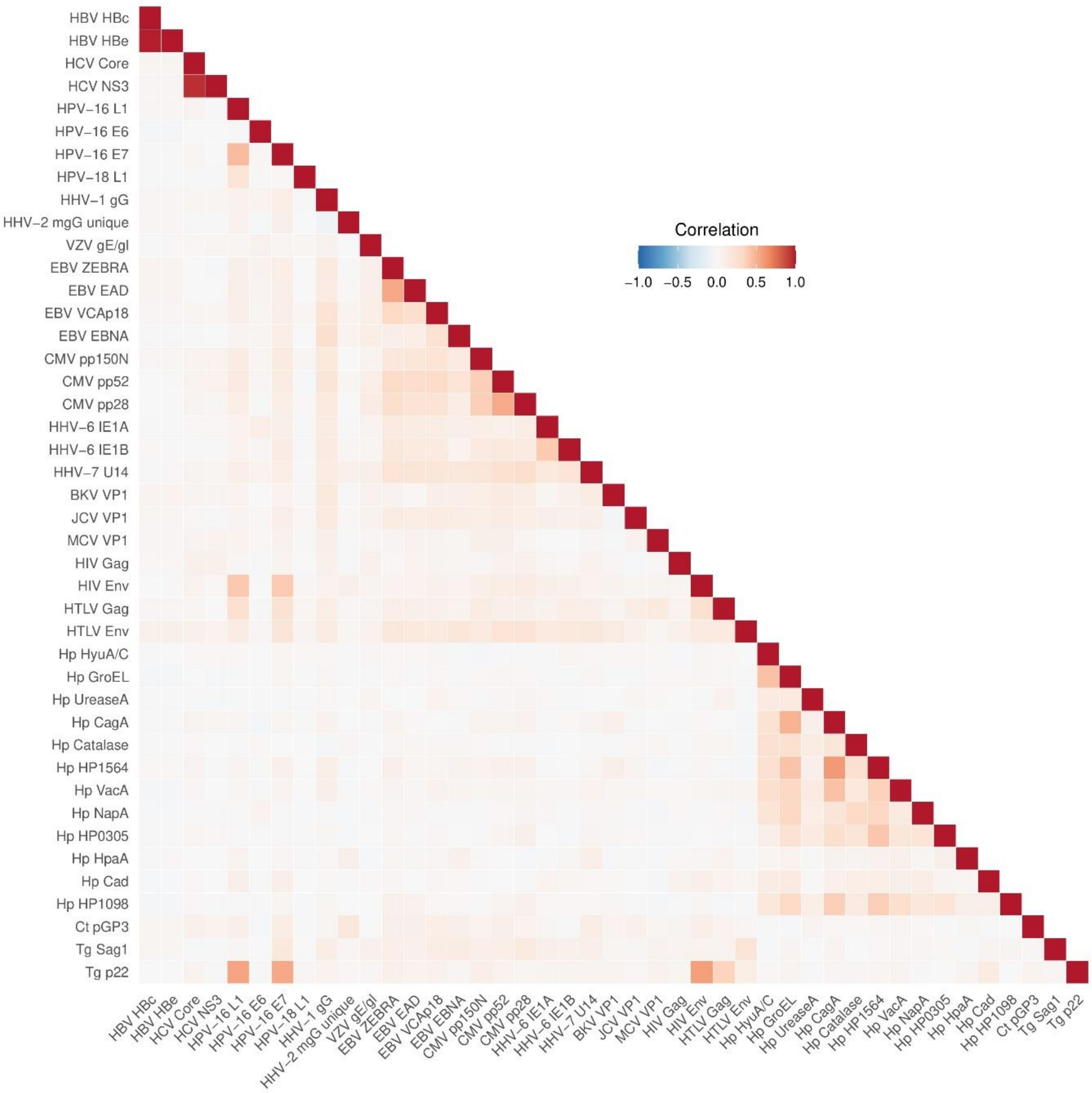

Figure S5. Intraclass correlation coefficient for MFI of 43 antigens between baseline and the 1st resurvey number of infections

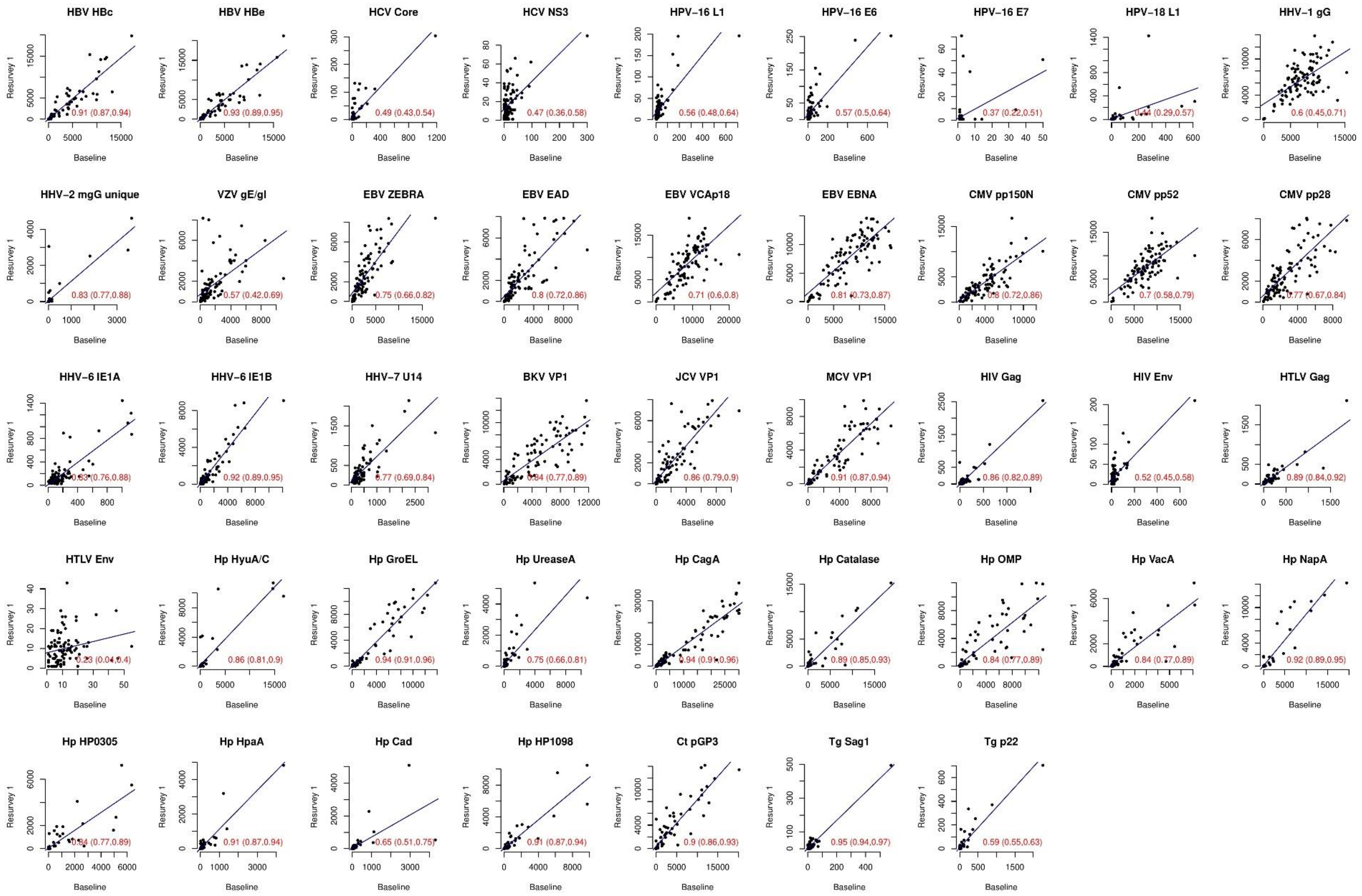

Figure S6. Intraclass correlation coefficient for MFI of 43 antigens between baseline and the 2nd resurvey

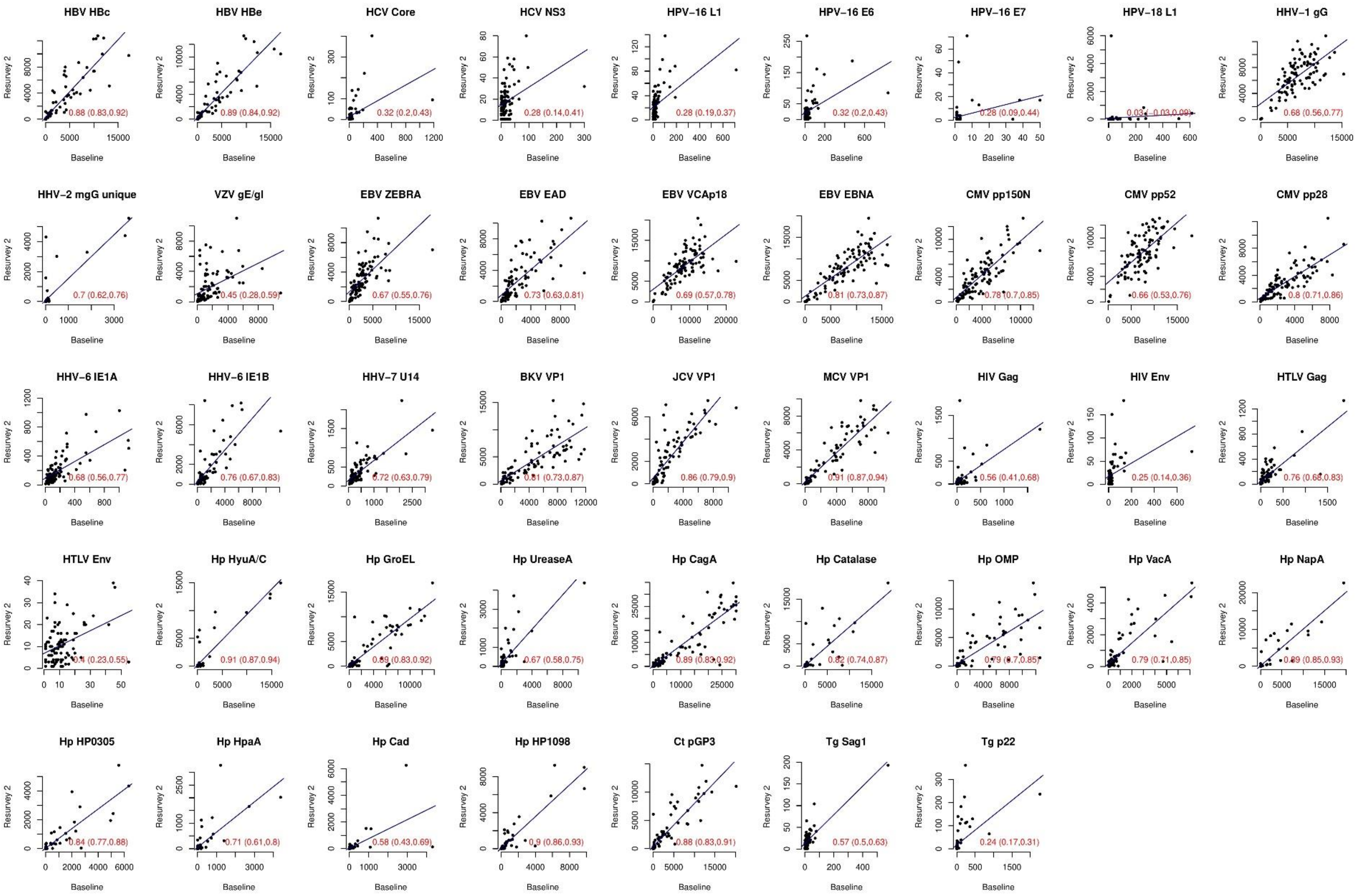

Figure S7. Intraclass correlation coefficient for MFI of 43 antigens between the 1st and the 2nd resurvey

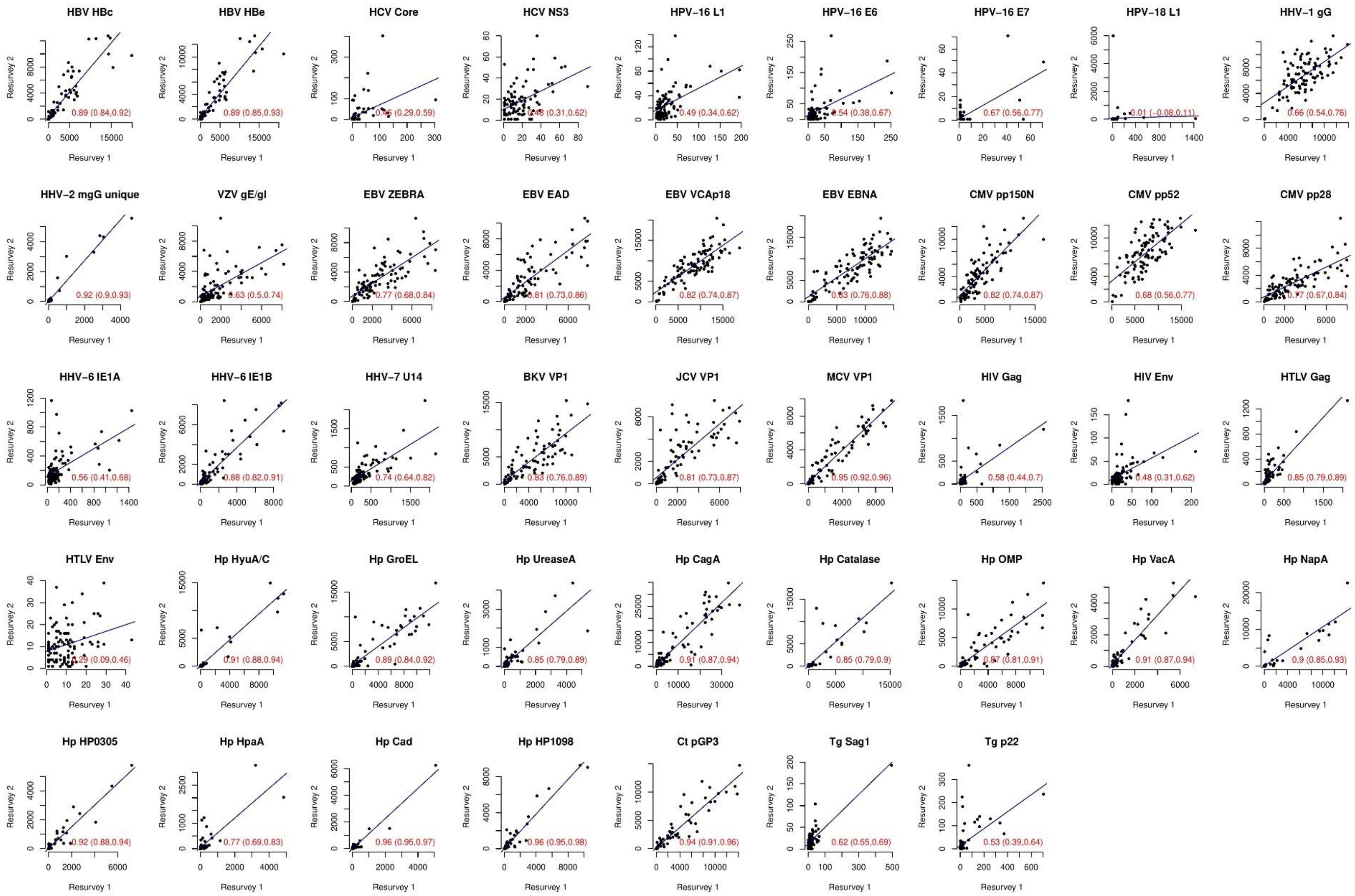

STROBE Statement—Checklist of items that should be included in reports of ***cross-sectional studies***

	Item No	Recommendation	Page No
**Title and abstract**	1	(*a*) Indicate the study's design with a commonly used term in the title or the abstract	1
		(*b*) Provide in the abstract an informative and balanced summary of what was done and what was found	2
**Introduction**			
Background/rationale	2	Explain the scientific background and rationale for the investigation being reported	4-5
Objectives	3	State specific objectives, including any prespecified hypotheses	5
**Methods**			
Study design	4	Present key elements of study design early in the paper	5
Setting	5	Describe the setting, locations, and relevant dates, including periods of recruitment, exposure, follow-up, and data collection	5
Participants	6	(*a*) Give the eligibility criteria, and the sources and methods of selection of participants	5
Variables	7	Clearly define all outcomes, exposures, predictors, potential confounders, and effect modifiers. Give diagnostic criteria, if applicable	5
Data sources/ measurement	8*	For each variable of interest, give sources of data and details of methods of assessment (measurement). Describe comparability of assessment methods if there is more than one group	5-6
Bias	9	Describe any efforts to address potential sources of bias	6-8
Study size	10	Explain how the study size was arrived at	5-6
Quantitative variables	11	Explain how quantitative variables were handled in the analyses. If applicable, describe which groupings were chosen and why	6-7
Statistical methods	12	(*a*) Describe all statistical methods, including those used to control for confounding	7-8
		(*b*) Describe any methods used to examine subgroups and interactions	7-8
		(*c*) Explain how missing data were addressed	NA
		(*d*) If applicable, describe analytical methods taking account of sampling strategy	5-6
		(*e*) Describe any sensitivity analyses	NA
**Results**			
Participants	13*	(a) Report numbers of individuals at each stage of study—eg numbers potentially eligible, examined for eligibility, confirmed eligible, included in the study, completing follow-up, and analysed	5
		(b) Give reasons for non-participation at each stage	5, S2
		(c) Consider use of a flow diagram	NA
Descriptive data	14*	(a) Give characteristics of study participants (eg demographic, clinical, social) and information on exposures and potential confounders	5
		(b) Indicate number of participants with missing data for each variable of interest	NA
Outcome data	15*	Report numbers of outcome events or summary measures	8-10
Main results	16	(*a*) Give unadjusted estimates and, if applicable, confounder-adjusted estimates and their precision (eg, 95% confidence interval). Make clear which confounders were adjusted for and why they were included	10

For peer review only - http://bmjopen.bmj.com/site/about/guidelines.xhtml

		(*b*) Report category boundaries when continuous variables were categorized	7
		(*c*) If relevant, consider translating estimates of relative risk into absolute risk for a meaningful time period	NA
Other analyses	17	Report other analyses done—eg analyses of subgroups and interactions, and sensitivity analyses	7-8
**Discussion**			
Key results	18	Summarise key results with reference to study objectives	11
Limitations	19	Discuss limitations of the study, taking into account sources of potential bias or imprecision. Discuss both direction and magnitude of any potential bias	18
Interpretation	20	Give a cautious overall interpretation of results considering objectives, limitations, multiplicity of analyses, results from similar studies, and other relevant evidence	11-18
Generalisability	21	Discuss the generalisability (external validity) of the study results	11-18
**Other information**			
Funding	22	Give the source of funding and the role of the funders for the present study and, if applicable, for the original study on which the present article is based	19

*Give information separately for exposed and unexposed groups.

Note: An Explanation and Elaboration article discusses each checklist item and gives methodological background and published examples of transparent reporting. The STROBE checklist is best used in conjunction with this article (freely available on the Web sites of PLoS Medicine at http://www.plosmedicine.org/, Annals of Internal Medicine at http://www.annals.org/, and Epidemiology at http://www.epidem.com/). Information on the STROBE Initiative is available at www.strobe-statement.org.

For peer review only
